# Atomistic Insights into Conformations and Solvation Dynamics of Amylose, Dextran, and Pullulan Using Three Force Fields

**DOI:** 10.1021/acs.jpcb.5c08146

**Published:** 2026-04-01

**Authors:** Parisa Farzeen, Hu Young Yoon, Isabela Trindade Coutinho, Maren Roman, Robert Moore, Sanket A. Deshmukh

**Affiliations:** † Department of Chemical Engineering, 1757 Virginia Tech, Blacksburg, Virginia 24061, United States; ‡ Macromolecules Innovation Institute, Virginia Tech, Blacksburg, Virginia 24061, United States; § Department of Chemistry, Virginia Tech, Blacksburg, Virginia 24061, United States; ∥ Department of Sustainable Biomaterials, Virginia Tech, Blacksburg, Virginia 24061, United States

## Abstract

Molecular dynamics (MD) simulations of three α-glucans—amylose, dextran, and pullulan—were performed in explicit solvent to investigate how differences in their glycosidic linkages influence molecular conformation and solvation dynamics, and to assess the performance of three major force fields (FFs): CHARMM36, GLYCAM06, and OPLS-AA. Structural analysis revealed that amylose adopts a more extended and constrained conformation owing to its α-(1 → 4) linkages, whereas dextran and pullulan exhibit more collapsed structures. Differences in linkage chemistry also influenced the organization of surrounding water, with this effect more pronounced in CHARMM than in GLYCAM or OPLS. Dextran formed more stable hydrogen bonds with water than amylose or pullulan. Although glucan-water nonbonded interactions were energetically more favorable in GLYCAM and OPLS, these FFs also predicted stronger nonpolar interactions that promoted more compact glucan conformations. Overall, the results indicate that glucan–glucan interactions contribute comparably to glucan-water interactions in determining polysaccharide structure. These insights clarify molecular determinants of glucan solubility and hydration and provide a basis for designing glucan-based materials with tunable properties.

## Graphical abstract

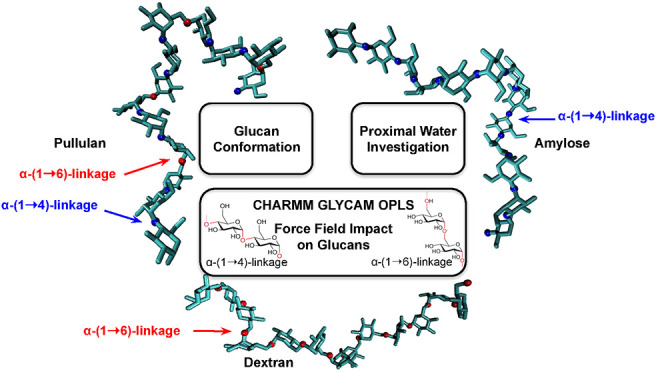

## 1 Introduction

Polysaccharides and related materials have gained widespread popularity across diverse fields, including biomedical, food, cosmetics, printing, plastics, and pharmaceuticals, owing to their biocompatibility, biodegradability, low toxicity, and ease of modification.^1–4^ Among these materials, α-glucans stand out for their versatile structural configurations and functional properties. Naturally occurring α-glucans, such as dextran, amylose, and pullulan, share the same monomeric unit, d-glucose, but are distinguished by their unique glycosidic linkages. Dextran is primarily characterized by α-(1 → 6) linkages in its main chain, amylose by α-(1 → 4) bonds, and pullulan by a sequence of α-(1 → 6)-linked α-(1 → 4)-d-triglucosides (Figure 1).^2,5^ These distinct glycosidic linkages result in unique molecular structures and properties, including water solubility. Dextran (MW <500 kDa) is highly water-soluble,^6,7^ amylose is poorly water-soluble,^4,8,9^ and pullulan readily dissolves in water to form a viscous solution.^10^ These differences in water solubility are largely determined by the presence of intra- and interchain hydrogen bonds,^5,11^ as well as intrachain hydrophobic interactions,^4,12–14^ ultimately influencing swelling, gelation, and dissolution behavior. As a result, α-glucans find broad applications in areas such as drug delivery systems (controlled release hydrogels),^3,15^ food stabilizers (thickening and gelling agents),^16,17^ and many more.

**Figure 1:**
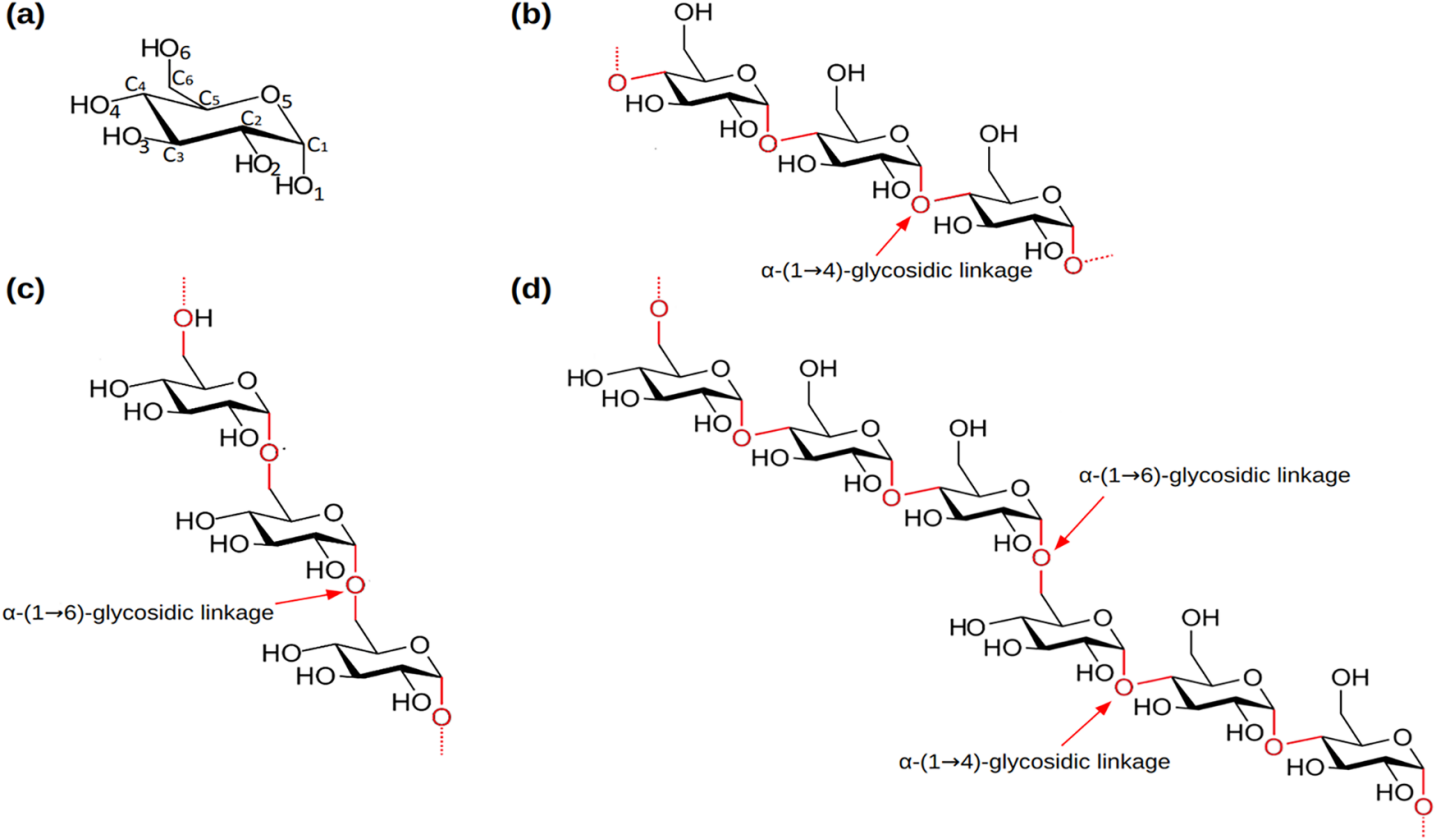
Chemical structure of (a) α-d-glucopyranose, (b) amylose, (c) dextran, and (d) pullulan.

While previous studies have provided valuable macroscopic insights,^4,18,19^ a deeper molecular-level understanding of the solvation dynamics of dextran, pullulan, and amylose remains limited. A key gap lies in understanding how differences in glycosidic linkages govern the diverse solubility patterns observed in these glucans. Addressing this gap is critical for designing new glycomaterials with tailored solubility and functionality. To bridge this gap, we raise several fundamental questions: (1) How do different glycosidic linkages influence glucans’ molecular conformations and flexibility or rigidity in an aqueous environment? (2) What is the structure of proximal water at the glucan-water interface? (3) Finally, how do inter- and intramolecular nonbonded interactions, such as hydrogen bonds and hydrophobic interactions, shape their conformations?

We employ all-atom (AA) molecular dynamics (MD) simulations to answer these questions.^18,20,21^ Experimental studies have shown that differences in glycosidic linkages among linear amylose, dextran, and pullulan lead to variations in molecular flexibility or rigidity, which in turn affect nonbonded interactions and solubility. In this study, we provide molecular insights into these effects and elucidate their origins using MD simulations. Note that in this study, we limited our scope to α-linked glucans, which provide a representative framework for assessing force field (FF) performance and linkage-dependent conformational and solvation behavior.

In recent years, AA MD simulations have been widely employed to study glucan conformation by comparing simulation results with experimental data on physical, dynamic, structural, and thermodynamic properties.^18,22–30^ Given that the accuracy of MD simulations heavily depends on the choice of FF parameters, and with growing interest in polysaccharides, several studies have evaluated the performance of FFs against experimental benchmarks. For example, a comparison of CHARMM36-TIP3P, GROMOS56A6_CARBO_-SPC, GLYCAM06h-TIP3P, and GLYCAM06h-TIP5P revealed significant differences in the diffusion coefficient, density, and hydration free energy for hemicellulose building blocks.^24^ Among these, GLYCAM06h-TIP5P showed the best agreement with the experimental data, attributed to the enhanced solute–solvent affinity from stronger electrostatic interactions. Koneru et al.^25^ assessed the ability of CHARMM36, GLYCAM06, OPLS-AA, and GROMOS53A6_CARBO__R to model the conformational landscape of a 12-mer amylose single-strand. Their simulations showed that GLYCAM06 and CHARMM36 predicted a pronounced helix–coil transition, while the remaining two FFs favored an extended conformation.^25^ Lazar et. al.^26^ studied L-rhamnose-rich polysaccharides using GLYCAM06 and CHARMM36 FFs and observed a conformational collapse with GLYCAM06 that was absent in CHARMM36 simulations. They attributed this behavior to the lack of partial charges on the aliphatic hydrogens in GLYCAM06, a feature present in CHARMM36.^26^ Balogh et. al.^27^ compared GAFF1, GLYCAM06, and CHARMM in reproducing the conformational ensembles of idraparinux, an anticoagulant, and found that CHARMM provided the closest match to experimental data. Similarly, an MD simulations study on heparin sulfate using CHARMM36 and GLYCAM06 reported generally comparable results, with minor but important differences in torsion angles, hydrogen-bonding patterns, and water bridge dynamics.^29^ Additionally, CHARMM, AMBER94, and AMBER-GLYCAM04 FFs have been used to investigate temperature-dependent conformational transitions of glucans.^30^ Overall, these studies have provided critical insights into glucan behavior, while also emphasizing the importance of selecting appropriate FFs, each of which is parametrized using distinct strategies to capture specific properties.

In this study, we not only address the fundamental aspects of glucan solubility but also investigate the impact of FF choice on glucan conformation and solubility characteristics. Specifically, we employ three widely used atomistic carbohydrate FFs: CHARMM36,^31^ GLYCAM06j,^32^ and OPLS all-atom (OPLS).^33^ The CHARMM36 FF is known for its accuracy in reproducing crystal lattice parameters, intramolecular geometries, aqueous densities, and NMR coupling constants associated with glycosidic linkages.^31^ The GLYCAM06 FF, with its single parameter set for both α- and β-anomers across all monosaccharide ring sizes and conformations, effectively reproduces gas- and condensed-phase rotamer populations, crystal packing, and vibrational spectra.^32^ The OPLS-AA was parametrized to capture the energies and structures of multiple conformers, accurately reproduce ab initio energy profiles, α and β-anomers ratios, and relative energies of isomeric hexopyranoses.^33^ Although all three FFs have been used in prior studies of glucans, to the best of our knowledge, this work represents the first systematic investigation of the solution behavior of linear dextran, amylose, and pullulan chains in explicit water while concurrently evaluating the performance of all three FFs.

This paper is organized as follows: In Section 2, we describe the simulation setup for the 30-mer chains of dextran, amylose, and pullulan in explicit water, and the experimental method for obtaining *R*_g_ and *R*_h_ of dextran. Section 3 presents a detailed discussion of our findings that directly addresses the three aforementioned questions. Specifically, to address Question 1, we analyzed glucan conformations by examining the radius of gyration (*R*_g_), the hydrodynamic radius (*R*_h_), dihedral angle distributions, and vibrational spectra of the glycosidic linkages. Question 2 is addressed by investigating the structure and organization of proximal water via solvent accessible surface area (SASA), radial distribution functions (RDFs), and the number of proximal water molecules in the hydration shell. Subsequently, to address Question 3, we performed a detailed hydrogen bond analysis and investigated both glucan–glucan and glucan-water nonbonded interactions, including hydrophobic and polar contributions, by computing interaction energies between polar and nonpolar groups of glucans and proximal water. A comprehensive comparison with available experimental data is performed to assess the accuracy and reliability of these three FFs in capturing structural and solvation behavior. Finally, Section 4 summarizes the conclusions of this study.

## 2 Materials and Methods

### 2.1 MD Simulations Details

AA MD simulations of three glucans were performed using CHARMM36-TIP3P, GLYCAM06j-TIP3P, and OPLS-SPC/E, which are commonly used for studying oligosaccharides, polymers, and related biomaterials.^24–26,34–36^ All FF parameters for these water models are listed in Table S1 of the SI.^37^ Note that the choice of water model can influence both solvation behavior and glucan conformations.^37–39^ Because each FF is parametrized and validated in conjunction with a specific water model, applying a single water model across different FFs without reparameterization would disrupt the intended interaction balance and may introduce artifacts in both glucan conformations and the structure of the surrounding hydration shell. Therefore, using the compatible water model is critical.

MD simulations were performed using the GROMACS 2020.4 program.^40^ Initial stretched glucan chains of 30 monomers (30-mers) were generated with CHARMM-GUI^41–43^ and the doGlycans tools.^44^ This chain length is consistent with previous studies examining single chains of polysaccharides and polymers in aqueous solutions to analyze their conformations and the structure of proximal water.^9,25,37^ Similar to several reported studies on short-chain oligomers and polymers in different solvents, we employed three independent configurations of each glucan to minimize statistical errors and improve the reliability of the simulation results.^24,45–47^

Initially, we performed 5 ps NVT simulations in a cubic box with dimensions of 145 Å × 145 Å × 145 Å at 298 K in vacuum, using the stretched conformations. This allowed the glucan chains to relax and sample conformational space. From this vacuum trajectory, we selected three distinct random conformations based on differences in their end-to-end distances and visual inspection (Figures S1–S9) and used them as starting conformations to conduct three independent runs. These structures were solvated with ∼71,500 water molecules in a cubic box with dimensions of 130 Å × 130 Å × 130 Å. Because the glucan chains had already relaxed from their initially stretched conformations during the vacuum simulations, this solvation box size was sufficient to prevent interactions with its periodic images while allowing investigation of single-chain conformational dynamics, glucan-water interactions, and the structure of proximal water at the glucan-water interface, without introducing crowding effects. Use of AA MD simulations also allowed us to probe this glucan-water interface and the hydrogen-bonding interactions between glucans and water.

Periodic boundary conditions (PBC) with a 12 Å cutoff for van der Waals (vdW) and electrostatic interactions were applied, and the Particle-Mesh Ewald (PME)^48^ method was used for long-range electrostatics.^49^ Note that the studies that conduct such comparisons of these three FFs have used similar cutoff values.^25,26^ The LINCS^50^ algorithm was used to constrain bond lengths involving hydrogen atoms of glycans, and energy minimization was conducted using the steepest descent algorithm.^51^ The O–H bonds and the intramolecular H–H distance in both TIP3P and SPC/E water models were fixed using the SETTLE algorithm.^52^ The use of this algorithm to keep H–H distance fixed allowed us to run MD simulations with a 2 fs time step and preserved the internal geometry of water.^53^

Initially, the system was equilibrated for 100 ps in the NVT ensemble. This was followed by a 500 ns simulation in the NPT ensemble.^18,54^ The last 200 ns were chosen for ensemble averages and considered as equilibrated configurations to analyze the glucan conformations. Temperature was controlled at 298 K using the V-rescale thermostat with a 0.1 ps coupling constant, while pressure was maintained at 1 bar using the Parrinello–Rahman barostat with a 2 ps coupling constant.^55,56^ The atomic trajectories, including positions, velocities, and forces, were recorded every 1 ps and visualized using visual molecular dynamics (VMD).^57^ The trajectories from all three starting configurations were used to perform structural and dynamical analysis of polysaccharides and water systems, with results averaged over these three simulations.

For vibrational spectra analysis, the final configuration of the glucans from each run was used to initiate 50 ps MD simulations with a 0.5 fs time step in the NPT ensemble.^58^ Note that to ensure that the rigid bonds do not influence the vibrational spectra calculations, we conducted MD simulations without the SETTLE algorithm. Velocities were recorded at each time step to calculate the velocity autocorrelation function (VAF) over the 50 ps,^59^ and the vibrational spectra were obtained by Fourier transforming the VAF. The GROMACS tool, “*gmx velacc*”, was utilized to calculate the vibrational spectra.^40^ The trajectories from three starting configurations were used to calculate the average vibrational spectra for the glucans.

### 2.2 Experimental *R*_h_ and *R*_g_ Determination

To validate the *R*_g_ and *R*_h_ predictions of the three selected FFs, we performed experiments to measure the *R*_g_ and *R*_h_ of dextran with a nominal molecular weight of 5000 g/mol, consistent with the simulated system. Specifically, *R*_h_ was measured using dynamic light scattering, while *R*_g_ was determined using small-angle and midangle X-ray scattering (SAXS and MAXS). More details on sample preparation and these measurements are provided in Section S1 of the SI.

## 3 Results and Discussion

### 3.1 Structural Analysis of Glucans

#### 3.1.1 Glucan Conformations and Size

To address how differences in glycosidic linkage topology influence glucan conformations and flexibility in aqueous solutions, we first quantified the overall size and shape of the glucan chains using the *R*_g_ and *R*_h_. These quantities provide complementary measures of polymer compactness and solvent-exposed conformations, and their ratio (*R*_g_/*R*_h_) is commonly used to distinguish between Gaussian coil-like, extended, and compact chain conformations.^20,60,60^ Here, a general power law relationship of the form presented by Fetters et al.^62^ was used to determine 30-mer glucan *R*_g_ and *R*_h_ values using experimental data obtained from the literature to compare simulation results to experimental data. Details and results of this can be found in Section S2, Table S5, and Table S8. In addition, we experimentally measured *R*_g_ and *R*_h_ for ∼30-mer dextran (5000 g/mol) using the approach discussed in Section S1, obtaining 14.0 ± 1.0 and 13.1 ± 1.2 Å, respectively. Table 1 shows results for <*R*_g_>, <*R*_h_>, and *R*_g_/*R*_h_ values obtained from the last 200 ns simulations, experimental data for ∼5000 g/mol dextran from this work, and experimental fitting. Figures S1–S9 and Movies M1, M2, M3, M4, M5, M6, M7, M8 and M9 show the dynamic evolution of all three glucans during the 500 ns simulation, conducted with three different FFs for one of three independent runs. Tables S3 and S6 present the ensemble-averaged values of *R*_g_ and *R*_h_, respectively, at different timesteps, along with RSE, and Tables S4 and S7 display the block-average *R*_g_ and *R*_h_ for each run, respectively.

**Table 1:** <*R*_g_> and <*R*_h_> Values for Different Glucan-Water Systems, over the Last 200 ns

		analysis over 300–500 ns		
force field	glucan	*R*_g_ (Å)	*R*_h_ (Å)	^a^ *R*_g_/*R*_h_
	Amylose	18.1 ± 1.1	15.5 ± 0.6	1.2
CHARMM	Dextran	17.4 ± 0.5	15.5 ± 0.3	1.1
	Pullulan	18.2 ± 1.1	15.6 ± 0.6	1.2
	Amylose	16.4 ± 4.3	14.0 ± 1.9	1.2
GLYCAM	Dextran	10.1 ± 0.3	11.1 ± 0.2	0.9
	Pullulan	9.7 ± 0.4	10.7 ± 0.3	0.9
	Amylose	19.9 ± 7.7	15.5 ± 3.5	1.3
OPLS	Dextran	10.2 ± 0.1	10.9 ± 0.0	0.9
	Pullulan	9.6 ± 0.1	10.6 ± 0.1	0.9
predicted using power law	Amylose	26.7		
	Dextran	22.0	23.0	1.0
	Pullulan	22.4	19.3	1.2
experiments this work	Dextran	14.0 ± 1.0	13.1 ± 1.2	1.1

a For reference, an *R*_g_/*R*_h_ value of 0.77 corresponds to a solid sphere, and 1.17 represents a Gaussian coil.^63^

##### 3.1.1.1 Amylose *R*_g_ and *R*_h_

Amylose consistently exhibited a high *R*_g_ and *R*_h_ with three FFs compared to dextran and pullulan. This trend is consistent with experimental reports showing a larger *R*_g_ value for amylose than dextran and pullulan at comparable molecular weights over a wide molecular weight range.^61,64–67^ Given amylose’s^8^ poor water solubility compared to dextran and pullulan, these higher *R*_g_ and *R*_h_ values contradict most water-soluble polymers, which typically adopt an extended or coil-like conformation only in favorable solvents.^60,68^ This consistently higher *R*_g_ and *R*_g_/*R*_h_ values observed for amylose across three FFs indicate a more rigid, extended coil-like conformation in water, likely arising from the restricted rotational freedom imposed by α-(1 → 4) glycosidic linkages.^18,69^ Among the three FFs, the OPLS and CHARMM results showed slightly better agreement with the experimentally fitted trends.

##### 3.1.1.2 Dextran *R*_g_ and *R*_h_

Dextran adopted a more expanded, coil-like conformation when modeled with the CHARMM FF, whereas more compact conformations were observed with GLYCAM and OPLS FFs. This increased compactness observed with GLYCAM and OPLS suggests FF dependent differences in the treatment of α-(1 → 6) glycosidic linkages relative to CHARMM FF, which modulates the effective chain flexibility observed in the simulations.^70^ Comparison with experimental power-law estimates (Table 1) shows that the predicted dextran dimensions overestimate experimentally measured values by approximately 24%. Despite predicting larger absolute *R*_g_ and *R*_h_ values, the CHARMM FF yielded *R*_g_/*R*_h_ ratios that were closer to experimental values than those from OPLS or GLYCAM.

##### 3.1.1.3 Pullulan *R*_g_ and *R*_h_

In the case of CHARMM FF, pullulan adopted a more expanded coil-like conformation, with *R*_g_ and *R*_h_ values comparable to those of amylose and dextran. In contrast, GLYCAM and OPLS predicted more compact pullulan conformations, indicating FF-dependent differences in the representation of α-(1 → 6) glycosidic linkages that alter local torsional sampling and, consequently, the balance between extended and compact chain conformations.^70^ The CHARMM FF showed better agreement with experimentally fitted data for pullulan chain dimensions compared to GLYCAM and OPLS.

Taken together, the *R*_g_ and *R*_h_ analyses address an important aspect of the first question posed in the Introduction, showing that glycosidic linkage topology strongly influences glucan chain size and flexibility, especially in GLYCAM and OPLS FFs. Glucans dominated by α-(1 → 4) linkages adopt more extended, rigid coil-like conformations, whereas α-(1 → 6)-linked glucans are more flexible and prone to compact conformations.

#### 3.1.2 Dihedral Angle Distribution of Glucans

To further elucidate the molecular origins of the differences in chain dimensions observed from the *R*_g_ and *R*_h_ analysis, we next examined the distributions of glycosidic linkage dihedral angles, which directly probe local torsional sampling and conformational preferences along the glucan backbone. Details of the glycosidic-linkage dihedral definitions, calculation procedures, and the corresponding dihedral angle distribution results for individual runs are provided in Section S2.2 of SI. Table 2 shows the comparison of the most probable glycosidic-linkage dihedral angle values for the three glucans for three FFs with available experimental data. The reported angle values represent the averaged response, along with standard deviations, from three independent trajectories.

**Table 2:** Most Probable Angle Values of the Glycosidic Linkage Torsion Angles^d^ ^,^ ^e^

		ϕ		ψ			ω		
force field	glucan link	ϕ_exo_	anti-ϕ	ψ_180_	ψ_90_	ψ_–90_	gg	gt	tg
CHARMM	Amylose 1–4	89.6 ± 1.2 (100)	-	-	88.6 ± 2.1 (100)	-	-	-	-
	Dextran 1–6	69.0 ± 0.0 (100)	-	-	96.6 ± 4.0 (26)	–18.0 ± 2.0 (74)	–8.0 ± 1.4 (58), 19.5 ± 9.2 (100)^a^	15.0 ± 9.8 (42)	-
	Pullulan 1–4	90.3 ± 0.6 (100)	-	-	90.0 ± 4.0 (100)	-	-	-	-
	Pullulan 1–6	70.0 ± 0.0 (100)	-	–168 ± 0.6 (5)	17.3 ± 0.6, 90.0 ± 2.0 (32)	–19.0 ± 0.0, –93.7 ± 0.3 (63)	–20 (8)^a^, –8.0 ± 0.0 (77)	6 (92)^a^, 12.0 ± 9.8 (23)	-
GLYCAM	Amylose 1–4	80.0 ± 2.6 (100)	-	-	84.7 ± 7.1 (100)	-	-	-	-
	Dextran 1–6	63.6 ± 0.6 (100)	-	-	45.0 ± 3.6 (2)	–85.0 ± 2.6, –61.7 ± 2.9 (98)	-	32.0 ± 3.6 (100)	-
	Pullulan 1–4	76.0 ± 2.6 (100)	-	-	74.6 ± 3.5 (100)	-	-	-	-
	Pullulan 1–6	73.3 ± 2.1 (100)	-	–156 ± 8.5 (17)	20 (7)^a^	–59.7 ± 11.6, –100 ± 14.8 (80)	–30 (3)^a^	59.3 ± 11.9 (98)	-
OPLS	Amylose 1–4	77.5 ± 5.6 (100)	-	-	87.3 ± 4.7 (100)	-	-	-	-
	Dextran 1–6	71.6 ± 0.6 (100)	-	-	-	–69.3 ± 2.1, –46.3 ± 1.5 (100)	-	47.0 ± 4.4 (100)	-
	Pullulan 1–4	78.0 ± 2.0 (100)	-	-	54, 82.3 ± 11.5 (100)	-	-	-	-
	Pullulan 1–6	73.0 ± 1.0 (100)	-	–153 ± 5.0 (14)	43.5 ± 17.6 (6)	–80.0 ± 3.5, –46.7 ± 2.8 (80)	-	84.0 ± 12.3 (100)	-
EXP	Amylose (maltose) 1–4	88.5 (NMR), 96.8 (X-ray)^b^			95.5 (NMR), 105.2 (X-ray)^b^		-	-	-
	Dextran 1–6	62.9^c^		–174.2(X-ray)^b^	68^c^		–53.8^c^		
		75.9^b^							

a observed for one of the three independent runs.

b Ref 70.

c Ref 71.

d ϕ_exo_: 0° < ϕ < 120°, ψ_180_: 120° < ψ < 180° and −180° < ψ < −120°, ψ_90_: 0° < ψ < 120°, ψ_–90_: −120° < ψ < 0°, ω_gg_: −120° < ω < 0°, ω_gt_: 0° < ω < 120°, ω_tg_: −180° < ω < −120° and 120° < ω < 180°.

e The percentage population of angles observed in the given range is shown in parentheses. Angles were obtained in the interval from −180° to 180°.

##### 3.1.2.1 Amylose Dihedral Angle Distribution

Across all three FFs, amylose consistently favored exoanomeric (0° < ϕ_exo_ < 120°) conformations for the ϕ dihedral angle, in agreement with the well-established exoanomeric effect reported in both experimental and computational studies.^70,72–74^ This preference places polar hydroxyl groups in an axial orientation relative to the glucan ring and reflects strong local conformational constraints imposed by α-(1 → 4) glycosidic linkages. In addition, the ψ dihedral angle exhibited a strong preference for a single dominant conformation across all FFs, further restricting backbone torsional sampling.^18,70^ Together, these constrained ϕ and ψ torsional states are consistent with the more extended, coil-like conformations observed in the *R*_g_ and *R*_h_ analysis and highlight the inherently rigid nature of amylose chains in aqueous environments.

##### 3.1.2.2 Dextran Dihedral Angle Distribution

The ϕ dihedral in dextran exclusively adopts exoanomeric conformations (0° < ϕ < 120°), consistent with NMR and X-ray data and similar to amylose, indicating linkage-independent stabilization of these states.^70,73,74^ In contrast, all three FFs strongly sample high-energy ψ conformations (ψ ≈ ±90°), in disagreement with experimental observations that report dominant antiperiplanar populations (ψ ≈ 180°).^54,70,71^ This systematic bias in ψ sampling may reflect weaker torsional constraints on α-(1 → 6) linkages in the FFs, resulting in increased local rotational freedom and FF-dependent ω responses, where ω prefers gt rotamer when ψ is at high-energy conformations.^54^ CHARMM samples both gg and gt rotamers for ω, whereas GLYCAM and OPLS exclusively favor the gt state.^54,75^ While reduced torsional constraints allow access to more compact chain conformations, the persistence of high-energy ψ populations indicates the FF limitation rather than physically accurate ψ-dihedral energetics.

##### 3.1.2.3 Pullulan Dihedral Angle Distribution

For pullulan, all three FFs predict exoanomeric ϕ conformations with 100% population for both α-(1 → 6) and α-(1 → 4) linkages, consistent with previous CHARMM-based computational studies and similar to dextran and amylose.^18,54^ In contrast, the ψ dihedral for pullulan predominantly samples high-energy conformations across all three FFs, deviating from the experimentally preferred antiperiplanar state. CHARMM favors ψ ≈ 90° for α-(1 → 4) linkages and both ψ ≈ ±90° for α-(1 → 6) linkages, with GLYCAM and OPLS showing a strong preference for ψ ≈ −90° for α-(1 → 6) linkages. CHARMM retains a higher gg population for ω relative to GLYCAM and OPLS, which show near-exclusive (98–100%) gt preference.^18^ The increased sampling of high-energy torsional states, arising from reduced torsional constraints in the FFs, may lead to enhanced local flexibility of α-(1 → 6) linkages and is consistent with the compact chain conformations observed in size-based metrics.

Taken together, the dihedral angle analysis clarifies how glycosidic linkage chemistry and FF torsional energetics jointly govern the apparent flexibility of glucans. The α-(1 → 4)-linked amylose is characterized by well-defined low-energy dihedral states that restrict chain bending and lead to relatively rigid conformations. In contrast, α-(1 → 6)-linked dextran and pullulan exhibit increased apparent flexibility because the FFs may insufficiently penalize high-energy ψ and ω conformations, allowing these states to be sampled more frequently than suggested by experiment. This reduced torsional constraint may manifest as enhanced chain flexibility and more compact conformations in size-based metrics. Differences among FFs primarily reflect the extent of stabilization of these high-energy torsional states, with GLYCAM and OPLS favoring more flexible, collapsed conformations compared to CHARMM.

#### 3.1.3 Vibrational Spectra of Atoms in Glycosidic Linkages

To further probe how glycosidic linkage stereochemistry and intramolecular constraints shape conformational preferences, we computed vibrational spectra of atoms involved in the glycosidic linkages. Specifically, vibrational frequencies associated with C–O–C and C–O stretching modes are highly sensitive to local bonding environments, chain order, and conformational constraints, and therefore provide a molecular-level signature of linkage geometry and flexibility.^76,77^ By comparing the simulated vibrational spectra with available experimental data, this analysis enables assessment of which atomistic FFs accurately capture experimentally observed, linkage-specific vibrational features. Details on the vibrational spectra analysis method can be found in Section S2.3 of the SI. The reported peak positions, which correspond to different frequencies, are listed in Tables 3, 4, and 5, and vibrational spectra of each run and the averaged results are shown in Figures S16–S18.

**Table 3:** Observed Vibrational Peak Positions for Amylose^a^ ^,^ ^b^

		C_1_ (cm^–1^)			C_4_ (cm^–1^)		
experimental peaks (cm^–1^)	peak assignment based on reported experimental data	CHARMM	GLYCAM	OPLS	CHARMM	GLYCAM	OPLS
863	C–O–C linkage between residues and C–H^78,79^	-	844 ± 1.2	845 ± 2.2	-	-	845 ± 0.0 (s)
926	C–O–C band in α-1,4 glycosidic linkages^77,80^	-	926 ± 0.0	-	-	914 ± 0.0	915 ± 0.4
940	C–O–C band in α-1,4 glycosidic linkages.^81^	934 ± 0.7	-	930 ± 5.5	943 ± 4.0	-	-
982	C–O–C band in α-1,4 glycosidic linkages.^81^	-	-	-	-	-	-
992	Combination of C–C and C–O bonds.^77,82^	998 ± 1.2 (s)	998 ± 1.5	1000 ± 3.3	1000 ± 1.0	1003 ± 4.5 (s)	1005 ± 1.8 (s)
1022	C–O stretching in amorphous structures^76,80,83^	1027 ± 6.8	-	-	-	1019 ± 2.1	1019 ± 2.5
1047	C–O stretching in crystalline structure^76,80,84^	1074 ± 2.3	1050 ± 0.3	1052 ± 0.5	1040 ± 2.3	1039 ± 5.8 (s)	1034 ± 4.7 (s)
1107	C–O–C asymmetric stretching^76,85,86^	1110 ± 1.2	1120 ± 0.4	1115 ± 2.7	1085 ± 7.5	1118 ± 1.5	1115 ± 2.2
1151	Combination of C–C and C–O stretching vibrations^77,82,84,87–89^	1146 ± 0.5 (s)	-	-	1155 ± 3.1 (s)	1153 ± 2.1	-
1165	C–O–C stretching vibration^77,90^	1172 ± 0.6	1172 ± 2.1	1176 ± 1.1	1180 ± 10.1 (s)	1172 ± 1.1 (s)	1165 ± 6.2

a Experimental bands, simulated bands from different FFs, and literature assignments of each band are shown.

b (s) shoulder.

**Table 4:** Observed Vibrational Peak Positions for Dextran^a^ ^,^ ^b^

		C_1_ (cm^–1^)			C_6_ (cm^–1^)		
experimental peaks (cm^–1^)	peak assignment based on reported experimental data	CHARMM	GLYCAM	OPLS	CHARMM	GLYCAM	OPLS
823	C–H vibration^79^	-	-	-	-	-	-
868	Anomeric C–H group vibration^79^	862 ± 0.6 (s)	862 ± 6.1 (s)	865 ± 1.8 (s)	872 ± 2.3 (s)	860 ± 2.5 (s)	-
924	C–O–C band in α-1,6 glycosidic linkages^79^	923 ± 2.1	927 ± 2.3	930 ± 3.3	940 ± 4.5	926 ± 5.1	925 ± 1.4
1018	C–O stretching in amorphous structures^76^	-	1005 ± 0.2	1000 ± 2.2	985 ± 2.1	-	1017 ± 0.7 (s)
1035	crystalline phase^76^	1040 ± 4.2	1053 ± 0.6 (s)	1037 ± 5.5 (s)	1050 ± 2.0	1028 ± 4.6	1028 ± 2.3
1080	complex vibrations due to C6–O6 bond stretching^76^	-	-	-	1087 ± 1.1	-	-
1111 (s)	vibrations of the C–O bond^79^	1130 ± 2.0	1113 ± 2.8	1113 ± 0.6	1136 ± 3.3	1127 ± 1.6	1126 ± 0.6
1162	Antisymmetric stretching vibrations due to the C–O–C glycosidic bridge^76^	1171 ± 1.6	1166 ± 1.1	1169 ± 0.3	-	1175 ± 1.3 (s)	1181 ± 0.3 (s)
1218 (s)	C–O stretching band^79^	1233 ± 0.4	-	-	1219 ± 0.5	-	-

a Experimental bands, simulated bands from different FFs, and literature assignments of each band are shown.

b (s) shoulder.

**Table 5:** Observed Vibrational Peak Positions for Pullulan (C, G, O Indicates CHARMM, GLYCAM, and OPLS FFs, Respectively)^a^ ^,^ ^b^

		C_1_ (cm^–1^)			C_4_ (cm^–1^)			C_6_ (cm^–1^)		
experimental peaks (cm^–1^)	peak assignment based on reported experimental data	C	G	O	C	G	O	C	G	O
850	C–H vibration^79^	-	848 ± 1.0	845 ± 2.1	875 ± 1.5 (s)	845 ± 0.2 (s)	-	884 ± 4.0	867 ± 0.1	-
930	C–O–C band in α-1,4 glycosidic linkages^77,80^	946 ± 3.6	926 ± 5.6	929 ± 3.1	948 ± 1.2	918 ± 3.5	911 ± 3.6	947 ± 2.1	924 ± 5.1	926 ± 4.6
992	Combination of C–C and C–O bonds.^77,82^	-	-	-	-	-	-	-	-	-
996	C–OH bending vibrations at the C6 position^76^	-	1002 ± 3.1	1000 ± 1.5	1004 ± 3.1 (s)	-	-	991 ± 0.5	-	-
1024	C–O stretching in amorphous structures^76,80,83^	1018 ± 2.0 (s)	-	-	-	1022 ± 2.2	1029 ± 2.5	-	1027 ± 5.5	1026 ± 3.8
1043	C–O stretching in crystalline structure^76,80,84^	1037 ± 2.5	1051 ± 0.2	1050 ± 2.2	1040 ± 5.1	1032 ± 1.8 (s)	-	-	1068 ± 2.3 (s)	-
1080	C6–O6 bond stretching^76^	-	-	-	1072 ± 8.2	-	-	1080 ± 3.2	-	1075 ± 2.1 (s)
1107	C–O bond vibrations at C4 ^76^	1120 ± 0.6	1120 ± 2.2	1117 ± 0.8	1106 ± 2.6 (s)	1119 ± 4.0	1117 ± 1.8	-	1121 ± 2.2	1120 ± 3.5
1155	Antisymmetric C–O–C stretching^77^	1163 ± 1.7	1170 ± 2.1	1173 ± 0.8	1148 ± 2.1 (s)	1171 ± 2.6 (s)	1156 ± 5.2 (s)	1144 ± 1.0	1172 ± 1.4	1176 ± 0.4 (s)
1210	C–O stretching band^79^	1230 ± 4.1	-	-	1202 ± 2.6	-	-	1209 ± 1.5	-	-

a Experimental bands, simulated bands from different FFs, and literature assignments of each band are shown.

b (s) shoulder.

##### 3.1.3.1 Vibrational Spectra of Amylose Atoms in Glycosidic Linkages

Table 3 and Figure S16 show the averaged vibrational spectra for the C_1_ and C_4_ carbon atoms of amylose. A band near 1022 cm^–1^, commonly associated with amorphous glucan environments,^76,80,83^ was observed across all FFs, suggesting the presence of locally disordered bonding environments. In contrast, a C–O stretching mode near 1047 cm^–1^, which has been linked to crystalline amylose environments,^76,80,84^ appeared at the C_4_ site for all three FFs. At C_1_, GLYCAM and OPLS reproduced this crystalline-associated mode near its experimentally reported position, whereas CHARMM exhibited a systematic shift to a higher frequency (∼1074 cm^–1^). This blue shift reflects stronger energetic constraints on local α-(1 → 4) glycosidic geometries in CHARMM. Overall, while all three FFs capture both amorphous and crystalline vibrational signatures of amylose, GLYCAM and OPLS show closer agreement with experimental peak positions, whereas CHARMM exhibits systematically higher-frequency crystalline-associated modes.

##### 3.1.3.2 Vibrational Spectra of Dextran Atoms in Glycosidic Linkages

Table 4 and Figure S17 present the vibrational spectra for the C_1_ and C_6_ atoms of dextran, which are directly involved in α-(1 → 6) glycosidic linkages and therefore probe both linkage-specific and exocyclic group vibrations. Bands near 1018 cm^–1^, which have been associated with amorphous polysaccharide environments,^76^ were more pronounced for GLYCAM and OPLS, indicating a higher population of locally disordered α-(1 → 6) bonding environments in these models. A band near 1035 cm^–1^, which has been attributed to crystalline or locally ordered dextran environments,^76^ shows clear FF dependence. This band was most intense at the C_1_ site for CHARMM, whereas GLYCAM and OPLS exhibit only weak shoulders. This reduced intensity for GLYCAM and OPLS may cause dextran to obtain a collapsed conformation with these two FFs. At higher frequencies, only CHARMM exhibits a distinct band near 1080 cm^–1^, attributed to C_6_–O_6_ bond stretching.^76^ The presence of this mode indicates stronger energetic constraints on exocyclic bond motions and is consistent with a more pronounced crystalline-like local environment around the α-(1 → 6) linkage. The absence of this feature in GLYCAM and OPLS reflects broader distributions of local C_6_–O_6_ bonding environments. Overall, while all three FFs reproduced the dominant experimental vibrational features of dextran, CHARMM showed stronger signatures of locally ordered α-(1 → 6) glycosidic environments, whereas GLYCAM and OPLS preferentially reflect amorphous local bonding motifs.

##### 3.1.3.3 Vibrational Spectra of Pullulan Atoms in Glycosidic Linkages

Table 5 and Figure S18 show the vibrational spectra for the C_1_, C_4_, and C_6_ atoms of pullulan, which contain alternating α-(1 → 4) and α-(1 → 6) glycosidic linkages. As a result, the vibrational spectra of pullulan reflect a superposition of features characteristic of both amylose- and dextran-like local environments. An amorphous-associated vibrational band near 1024 cm^–1^ is observed with CHARMM at the C_1_ site, whereas GLYCAM and OPLS show this feature predominantly at the C_4_ and C_6_ positions. This site dependence indicates differences in how amorphous local bonding environments are distributed across α-(1 → 4) and α-(1 → 6) segments in pullulan. The presence of this band across FFs suggests that locally disordered conformational motifs are sampled at multiple linkage positions. All three FFs also exhibit a peak near 1043 cm^–1^, which has been associated with crystalline or locally ordered polysaccharide environments.^76,80,84^ Differences in the relative intensities and site dependence of these bands reflect variations in the populations of locally ordered versus disordered linkage environments sampled by each FF. At higher frequencies, CHARMM shows a distinct band near 1080 cm^–1^ ^76^ attributed to C_6_–O_6_ bond stretching, while OPLS displays a weak shoulder near 1075 cm^–1^, and this feature is absent in GLYCAM. The clearer manifestation of this exocyclic stretching mode in CHARMM indicates stronger energetic constraints on C_6_–O_6_ bond motions and a higher population of locally ordered α-(1→6) environments. In contrast, the attenuation or absence of this in GLYCAM and OPLS reflects disordered C_6_–O_6_ environments.

Overall, all three FFs reproduced the primary amorphous and crystalline vibrational signatures of pullulan. However, CHARMM more consistently preserves vibrational features associated with locally ordered glycosidic environments, whereas GLYCAM and OPLS preferentially emphasize amorphous local bonding motifs. These results highlight how mixed α-(1 → 4)/α-(1 → 6) linkage chemistry leads to coexisting local conformational motifs that are differentially captured by atomistic FFs.

### 3.2 Structure of Interfacial Solvent

Several computational and experimental studies have suggested that solvent plays an important role in determining the conformations of glycosidic linkages, ultimately impacting the overall conformations of glycans.^91,92^ Despite this, most prior work, to the best of our knowledge, has focused on glucan conformations themselves rather than explicitly characterizing the structure of solvent at the glucan-water interface. To address how water is organized in the immediate vicinity of glucan chains, we analyzed the structure of proximal water using multiple complementary metrics derived from MD simulations.

#### 3.2.1 Radial Distribution Function

To directly probe the molecular structure of water at the glucan-water interface, we first analyzed radial distribution functions (RDFs) between glucan oxygen atoms and water oxygen (OW) atoms. RDFs provide spatially resolved information on the organization, density, and packing of water molecules around specific functional groups and therefore offer a direct means of characterizing the structure of the proximal solvent.^20,93,91,92^ By comparing hydration patterns around linkage-specific oxygen atoms across different glucans, this analysis directly addresses how glycosidic linkage chemistry influences interfacial water structure. The average RDF was obtained from three independent trajectories (last 200 ns). Figure 2 shows the RDF between glycosidic-linked oxygen and water, and Figure S19 shows the mean results with standard deviations.

**Figure 2:**
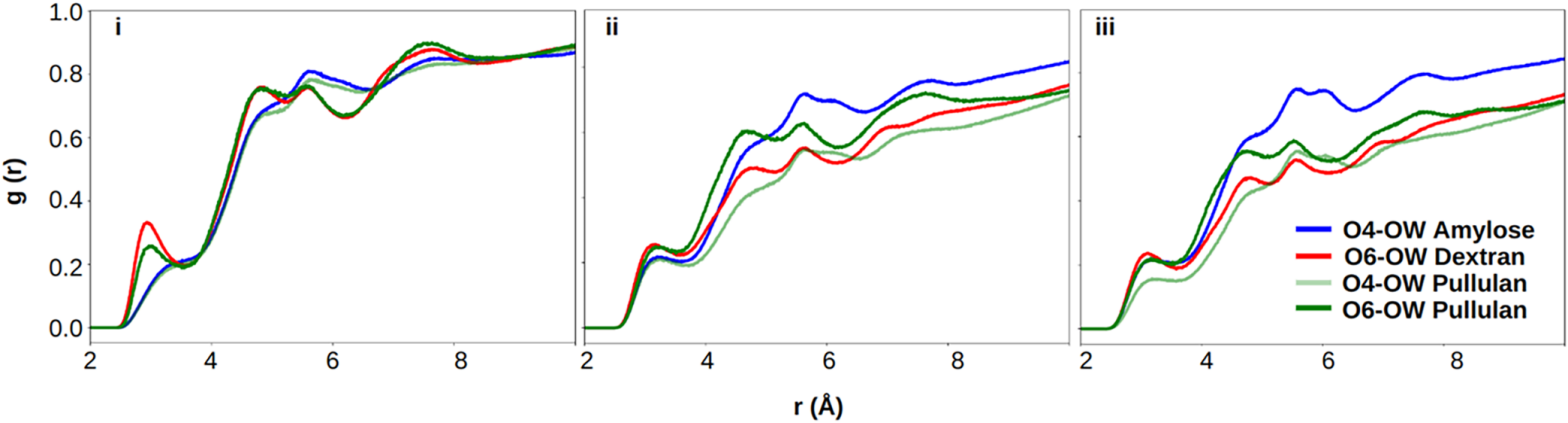
RDF between water-oxygens (OW) and glycosidic oxygens O_4_ in α-(1 → 4) linked amylose (blue), O_6_ in α-(1 → 6) linked dextran (red), O_4_ in α-(1 → 4) linked pullulan (light green), O_6_ in α-(1 → 6) linked pullulan (dark green) for (i) CHARMM, (ii) GLYCAM, and (iii) OPLS FF.

The RDF of dextran’s O_6_ in CHARMM displayed a higher peak intensity compared to GLYCAM and OPLS, suggesting enhanced hydration and more packed water molecules. This could contribute to the more extended conformation observed for dextran in CHARMM, as noted in Table 1. The broader and shifted hydration peaks observed for pullulan reflect the competing steric and conformational constraints imposed by its mixed α-(1 → 4) and α-(1 → 6) linkage architecture, which may disrupt efficient water packing relative to uniform linkage glucans. This observation aligns with that for amylose, where the first and second hydration shells exhibited higher peak intensities for O_4_ compared to pullulan’s O_4_ across all three FFs. This enhanced and well-defined hydration shell around amylose O_4_ is consistent with its more rigid backbone and sustained solvent exposure.

CHARMM appears to capture the influence of glycosidic linkage type on hydration more distinctly, suggesting stronger differentiation of linkage-dependent hydration patterns, while GLYCAM and OPLS exhibit comparatively more generalized hydration behavior. However, this distinction likely reflects differences in nonbonded parametrization in addition to conformational sampling, rather than an intrinsic superiority of any single FF. This highlights differences in each FF’s ability to capture the structure of proximal water molecules, with CHARMM capturing these linkage-dependent differences in proximal water structure more distinctly than GLYCAM and OPLS.

Figure 3 shows the RDFs between oxygen atoms in −OH groups and OW (solid line), as well as between ring oxygens and OW (dashed lines) for all three FFs. As expected, across all three FFs and for all glucans, the ring oxygens are less hydrated compared to −OH groups because the ring oxygens are part of the pyranose rings and might be shielded by neighboring groups. The distinction between hydration of −OH oxygens and ring oxygens further highlights the role of the local chemical environment in structuring interfacial water.

**Figure 3:**
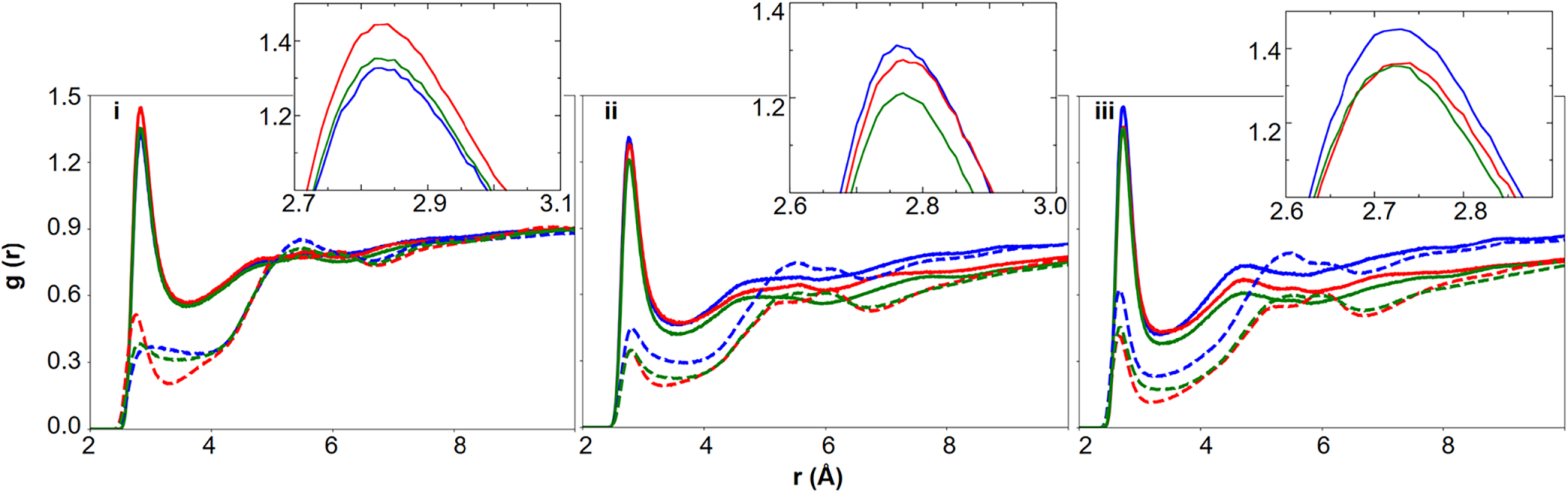
RDF between water-oxygen (OW) and −OH (solid) groups and ring-oxygen (O_5_) (dashed) of the glucans of 30-mer (blue) amylose, (red) dextran, and (green) pullulan using (i) CHARMM, (ii) GLYCAM, and (iii) OPLS.

CHARMM FF captured clear linkage-dependent shifts in ring-oxygen hydration distances, suggesting greater sensitivity to subtle conformational and steric effects imposed by glycosidic architecture. Both GLYCAM and OPLS suggest that the −OH groups in amylose are more hydrated compared to those in dextran and pullulan. Additionally, CHARMM exhibits broader peaks for both −OH and ring oxygens with water oxygen, indicating less structural correlation between glucan oxygen and surrounding water molecules. This can be attributed to both the influence of linkage geometry as well as FF specific glucan-water interaction parameters. The RDFs between ring oxygens for amylose and pullulan and OW show much broader peaks in CHARMM, which suggests that constrained conformations due to α-(1→4) linkages in amylose and sterically hindered conformations due to mixed α-(1 → 4) and α-(1→6) linkages in pullulan can be captured by CHARMM. This linkage may reduce the distance between adjacent monomer units, leading to reduced space for water to pack effectively, and thus the structural correlation between water and monomer.

To characterize the glucan-water interface and to quantify the extent and temporal stability of glucan hydration, we calculated the solvent accessible surface area (SASA) and number of water molecules near glucan chains, which showed overall agreement with the RDF data. The method of analysis and detailed discussion, along with results, can be found in Section S3.1 and S3.2, respectively.

### 3.3 Hydrogen Bond Analysis

Hydrogen bonding (H-bond) plays a central role in determining glucan conformation, solubility, and interactions with surrounding solvent, and can provide insights into the strength and stability of glucan-water and glucan–glucan interactions.^12,14^ To address how nonbonded interactions shape glucan conformations and how water interacts dynamically with glucan surfaces, we analyzed both intermolecular (glucan-water) and intramolecular (glucan–glucan) H-bond lifetimes using autocorrelation functions. Discussion on the calculation method can be found in Section S4.

#### 3.3.1 H-Bonds between Glycosidic-Linked Oxygens and Water

As shown in Figure 4 and Table S15, despite exhibiting the highest degree of hydration, amylose formed more transient H-bonds with water at its glycosidic oxygens, indicating that solvent accessibility does not necessarily imply stable glucan-water interactions. Conversely, dextran showed more stable H-bonds between glycosidic oxygen and water hydrogen compared to amylose and pullulan across all three FFs. This suggests that α-(1 → 6) linkage may facilitate and stabilize these H-bonds by promoting conformations that favor glucan-water H-bonds. FF-dependent differences further reveal that CHARMM favors more dynamic water interactions, whereas OPLS stabilizes glycosidic oxygen-water H-bonds. Figure S23 and Table S18 show data for each run.

**Figure 4:**
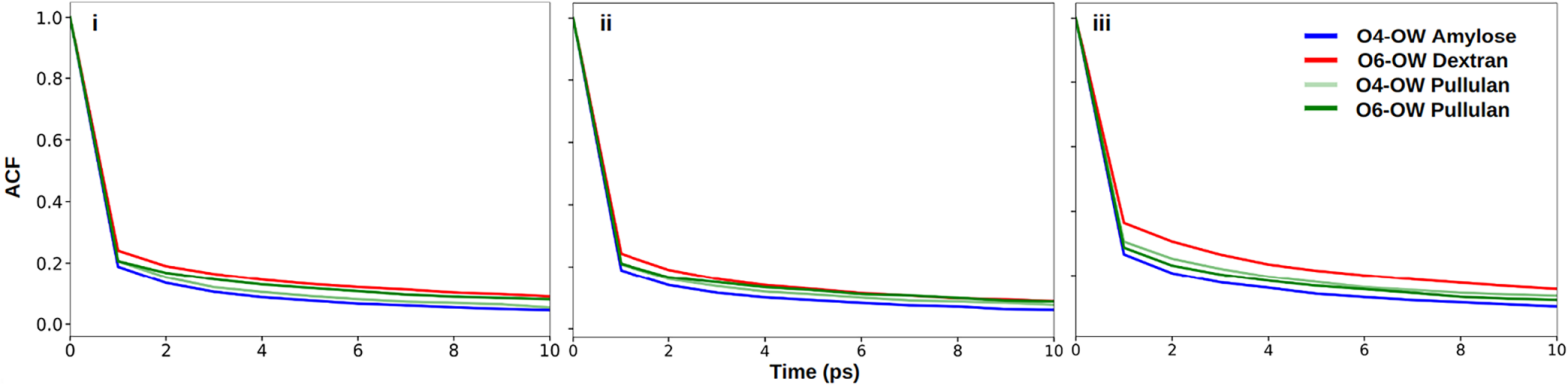
Autocorrelation functions for H-bonds between water and glycosidic oxygens O_4_ in α-(1 → 4) linked amylose (blue), O_6_ in α-(1 → 6) linked dextran (red), O_4_ in α-(1 → 4) linked pullulan (light green), O_6_ in α-(1 → 6) linked pullulan (dark green) using (i) CHARMM (ii) GLYCAM (iii) OPLS FFs.

#### 3.3.2 H-Bonds between Hydroxyl Groups of Glucans and Water

Figure 5A and Table S16 demonstrate that hydroxyl-mediated hydration is highly sensitive to both glycosidic linkage type and FF parametrization. Here, −OH groups are considered both acceptors and donors. Dextran consistently forms more stable H-bonds with water than amylose, reflecting enhanced rotational freedom and water accessibility. CHARMM predicts more dynamic H-bonding overall, while GLYCAM and OPLS stabilize hydroxyl-water interactions to a greater extent, particularly in mixed-linkage pullulan. Figure S24 and Table S19 show data for each run. These FF-dependent differences align with the enhanced intramolecular H-bond stabilization and compact conformations observed in GLYCAM and OPLS simulations.

**Figure 5:**
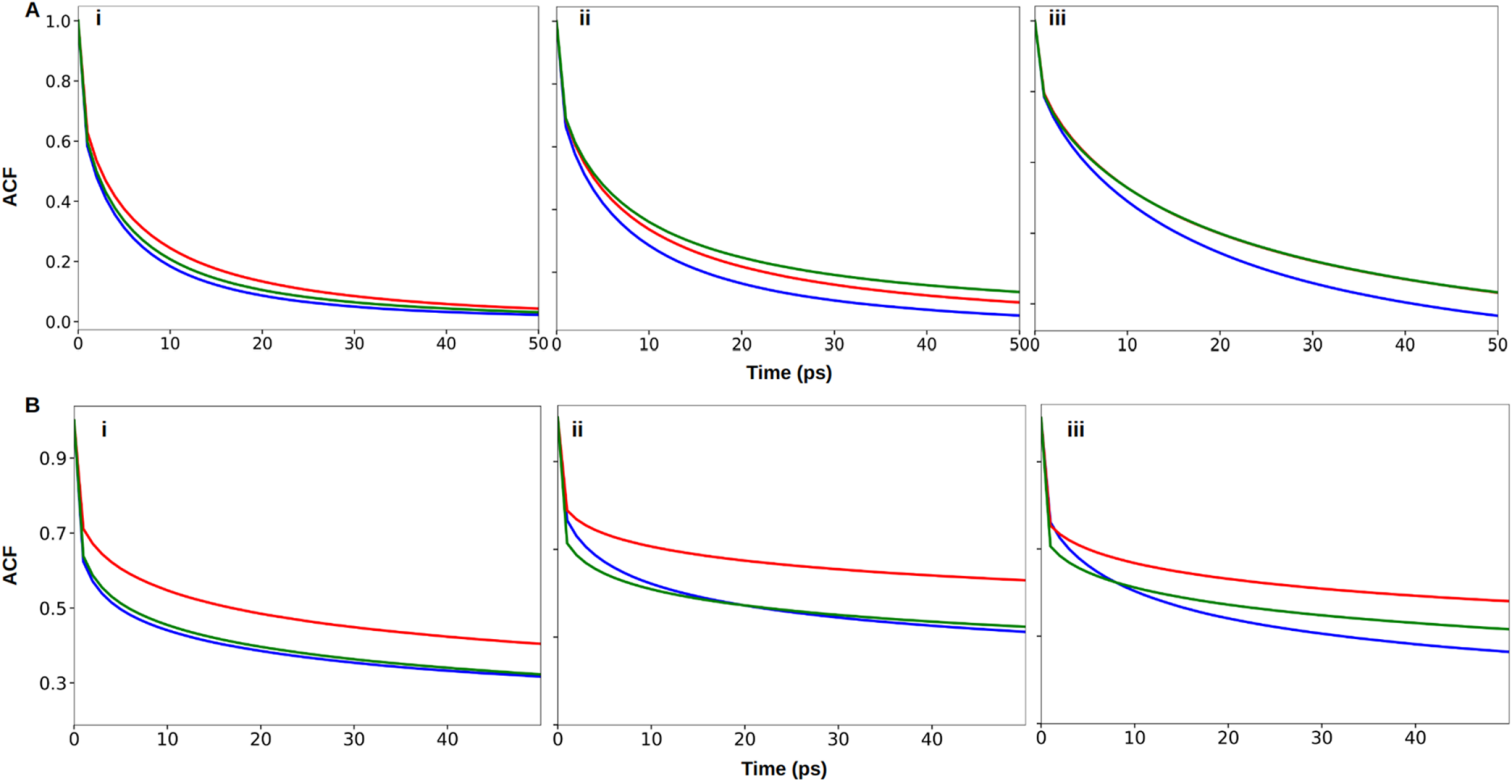
Autocorrelation functions for (A) H-bonds between water and −OH groups of the glucans and (B) H-bonds formed within glucan chains, amylose (blue), dextran (red), and pullulan (green) using (i) CHARMM, (ii) GLYCAM, (iii) OPLS FFs.

#### 3.3.3 Intramolecular H-Bonds

To better understand the interactions that stabilize glucan structures and conformations, we analyzed the intramolecular H-bonds formed within the glucan chains.^94,95^ Intramolecular H-bonds compete directly with glucan-water interactions and play a key role in stabilizing chain conformations and driving intramolecular collapse.^5,70,96^ The enhanced stability of intramolecular H-bonds in dextran and pullulan (Figure 5B and Table S17), particularly in GLYCAM and OPLS, explains their tendency toward more compact conformations observed in *R*_g_, SASA, and hydration analyses. The flexibility of α-(1 → 6) linkages may enable conformations that favor these stronger and more stable intramolecular H-bonds. In contrast, the more transient intramolecular H-bonds in amylose reflect the conformational constraints imposed by α-(1→4) linkages, which limit favorable intrachain contacts and promote extended chain conformations. Figure S25 and Table S20 show data for each run. Overall, H-bond analysis provided a mechanistic framework linking glycosidic linkage chemistry, FF-dependent conformational sampling, and interfacial solvent dynamics, thereby providing insights into the dynamic picture of glucan solvation behavior.

### 3.4 Nonbonded Interaction Energies

While structural and H-bond analyses reveal how glucan conformations and hydration differ at the molecular level, nonbonded (van der Waals forces and electrostatic) interaction energies provide a quantitative measure of the forces driving these behaviors.^19,97,98^ To directly assess how glucan-water and glucan–glucan interactions compete to determine chain conformation and solvation, we analyzed normalized nonbonded interaction energies from MD trajectories using the “*gmx energy*” tool.^40^ The normalized nonbonded interaction energies were obtained by dividing each energy value by the corresponding number of atoms in the glucan involved in the type of interaction.

#### 3.4.1 Glucan-Water Interaction Energies

We first examined glucan-water interaction energies to quantify the energetic favorability of hydration at specific chemical moieties and to rationalize the solvent accessibility and hydration patterns. Tables 6, 7, and 8 present the ensemble-averaged nonbonded interaction energies, along with RSE, between glycosidic oxygens and water (O_4_-water and O_6_-water interactions), between nonpolar atoms and water (nonpolar-water interactions), and between polar atoms and water (polar-water interactions), respectively. Tables S21–S23 show the data for each run, respectively. The data in here were normalized by the number of respective glucan atoms. Figure S26 in the SI shows the time evolution of polar-water and nonpolar water interaction energies.

**Table 6:** Ensemble-Averaged Values of Normalized Nonbonded Interaction Energies between Glucan Atoms (Glycosidic-Linked Oxygens) and Water^a^

			energy (kcal/mol) at different timesteps (ns)			
force field	Glucans	#of atoms used for normalization	301–350	351–400	401–450	451–500
CHARMM	Amylose O4-Water	29	–2.17 ± 0.05	–2.13 ± 0.09	–2.10 ± 0.07	–2.15 ± 0.06
			1.30	2.34	2.04	1.57
	Dextran O6-Water	29	–4.43 ± 0.14	–4.48 ± 0.10	–4.33 ± 0.13	–4.57 ± 0.02
			1.81	1.31	1.73	0.21
	Pullulan O4-Water	20	–2.30 ± 0.07	–2.23 ± 0.09	–2.22 ± 0.06	–2.27 ± 0.02
			1.77	2.44	1.62	0.60
	Pullulan O6-Water	9	–4.00 ± 0.16	–3.96 ± 0.14	–4.00 ± 0.09	–4.00 ± 0.16
			2.27	2.02	1.31	2.27
GLYCAM	Amylose O4-Water	29	–4.22 ± 0.29	–4.05 ± 0.38	–3.97 ± 0.28	–3.94 ± 0.28
			3.95	5.45	4.10	4.13
	Dextran O6-Water	29	–4.44 ± 0.19	–4.33 ± 0.14	–4.54 ± 0.06	–4.47 ± 0.35
			2.49	1.89	0.75	4.52
	Pullulan O4-Water	20	–3.27 ± 0.22	–3.12 ± 0.35	–3.15 ± 0.22	–3.17 ± 0.21
			3.97	6.52	3.96	3.82
	Pullulan O6-Water	9	–4.30 ± 0.19	–4.26 ± 0.23	–4.30 ± 0.34	–4.41 ± 0.28
			2.54	3.09	4.62	3.63
OPLS	Amylose O4-Water	29	–4.90 ± 0.55	–4.74 ± 0.61	–4.75 ± 0.58	–4.69 ± 0.64
			6.48	7.49	7.00	7.93
	Dextran O6-Water	29	–4.93 ± 0.22	–4.85 ± 0.14	–4.78 ± 0.24	–4.57 ± 0.24
			2.62	1.65	2.89	2.98
	Pullulan O4-Water	20	–3.52 ± 0.16	–3.43 ± 0.21	–3.45 ± 0.27	–3.42 ± 0.14
			2.71	3.52	4.48	2.42
	Pullulan O6-Water	9	–4.93 ± 0.21	–4.67 ± 0.24	–4.78 ± 0.24	–4.74 ± 0.28
			2.46	2.97	2.90	3.38

a The data is normalized by dividing the total energy by the number of respective atoms. The RSE is expressed as a percentage below the mean values.

**Table 7:** Ensemble-Averaged Values of Normalized Nonbonded Interaction Energies between Glucan Nonpolar Atoms and Water^a^

		energy (kcal/mol) at different timesteps (ns)			
force field	glucans	301–350	351–400	401–450	451–500
CHARMM	Amylose	0.13 ± 0.00	0.13 ± 0.00	0.13 ± 0.01	0.13 ± 0.01
		1.42	1.39	2.77	2.45
	Dextran	0.33 ± 0.01	0.34 ± 0.01	0.32 ± 0.01	0.34 ± 0.00
		2.50	2.20	1.84	0.20
	Pullulan	0.20 ± 0.00	0.19 ± 0.01	0.19 ± 0.01	0.19 ± 0.01
		0.94	3.00	1.61	2.82
GLYCAM	Amylose	0.56 ± 0.04	0.54 ± 0.05	0.53 ± 0.04	0.52 ± 0.03
		3.59	5.33	3.89	3.05
	Dextran	0.47 ± 0.02	0.46 ± 0.01	0.49 ± 0.01	0.48 ± 0.03
		2.47	0.92	0.72	3.59
	Pullulan	0.43 ± 0.03	0.43 ± 0.04	0.43 ± 0.03	0.44 ± 0.02
		4.38	5.99	4.38	3.01
OPLS	Amylose	0.86 ± 0.09	0.84 ± 0.10	0.84 ± 0.09	0.83 ± 0.11
		6.34	7.12	6.43	7.65
	Dextran	0.71 ± 0.02	0.69 ± 0.02	0.68 ± 0.02	0.66 ± 0.03
		2.02	1.69	1.91	2.70
	Pullulan	0.66 ± 0.03	0.64 ± 0.05	0.64 ± 0.05	0.63 ± 0.02
		3.04	4.85	4.76	2.27

a The data is normalized by dividing the total energy by the number of respective atoms (390). The RSE is expressed as a percentage below the mean values.

**Table 8:** Ensemble-Averaged Values of Normalized Nonbonded Interaction Energies between Glucan Polar Atoms and Water^a^

		energy (kcal/mol) at different timesteps (ns)			
force field	glucans	301–350	351–400	401–450	451–500
CHARMM	Amylose	–4.89 ± 0.11	–4.73 ± 0.14	–4.69 ± 0.16	–4.81 ± 0.11
		1.30	1.77	2.00	1.31
	Dextran	–5.56 ± 0.09	–5.58 ± 0.09	–5.43 ± 0.14	–5.67 ± 0.03
		0.97	0.94	1.45	0.36
	Pullulan	–5.02 ± 0.12	–4.96 ± 0.09	–4.98 ± 0.07	–5.04 ± 0.09
		1.39	1.04	0.86	1.06
GLYCAM	Amylose	–6.02 ± 0.37	–5.77 ± 0.43	–5.65 ± 0.35	–5.55 ± 0.29
		3.53	4.30	3.53	3.03
	Dextran	–5.03 ± 0.04	–5.02 ± 0.06	–5.18 ± 0.07	–5.08 ± 0.13
		0.43	0.67	0.83	1.46
	Pullulan	–4.80 ± 0.19	–4.72 ± 0.28	–4.78 ± 0.16	–4.84 ± 0.17
		2.29	3.40	1.98	2.05
OPLS	Amylose	–6.94 ± 0.65	–6.83 ± 0.69	–6.83 ± 0.65	–6.78 ± 0.75
		5.39	5.85	5.48	6.36
	Dextran	–5.87 ± 0.18	–5.77 ± 0.07	–5.73 ± 0.07	–5.62 ± 0.15
		1.76	0.70	0.66	1.52
	Pullulan	–5.55 ± 0.19	–5.44 ± 0.35	–5.45 ± 0.35	–5.47 ± 0.12
		2.00	3.71	3.67	1.26

a The data is normalized by dividing the total energy by the number of respective atoms (243). The RSE is expressed as a percentage below the mean values.

Tables 6 and S21 indicate that O_6_ atoms associated with α-(1 → 6) linkages engage more favorably with water than O_4_ atoms in α-(1 → 4) linkages, consistent with their enhanced flexibility and solvent accessibility. However, the magnitude of these differences varies across FFs, suggesting that both linkage geometry and FF-specific electrostatic parameters contribute to the observed trends. The poorer O_4_-water interaction observed for pullulan with GLYCAM and OPLS FFs, compared to CHARMM, could be due to the collapsed conformation observed with these FFs. Overall, glycosidic-linked O_4_-water interactions of pullulan were least favorable, which agrees with the RDF data shown in Figure 3, where this O_4_ of pullulans was least hydrated.

Nonpolar-water interactions were consistently unfavorable across all three force fields for all glucans, as indicated by their positive values. These consistently unfavorable nonpolar-water interactions indicate the dominant role of polar and H-bond-mediated interactions in determining glucan hydration and that conformational preferences arise from a balance between favorable polar hydration and intramolecular stabilization energies.

The stronger polar-water interactions in GLYCAM and OPLS for amylose, shown in Table 8, may explain the observed coil-like conformations, as seen in the higher *R*_g_ values. However, despite these stronger glucan-water interactions, GLYCAM and OPLS still promote more compact conformations for dextran and pullulan, indicating that intramolecular glucan–glucan interactions compete effectively with hydration in determining overall chain structure.^4^ This suggests that in addition to the rigid α-(1 → 4) linkages that dictate amylose’s constrained structure, its interactions with water may also play a significant role in determining its overall conformation.^99^

#### 3.4.2 Glucan–Glucan Interaction Energies

To determine whether intramolecular interactions contribute to the chain conformations, we next analyzed normalized intramolecular nonbonded interaction energies between polar and nonpolar glucan atoms.^4,19^ Tables 9 and 10 present the averaged, normalized nonbonded interaction energies for nonpolar-nonpolar and polar–polar groups of the three glucans across CHARMM, GLYCAM, and OPLS. Tables S24 and S25 show the data for individual runs. The values are normalized by the number of respective glucan atoms. Figure S27 in the SI shows the time evolution of these energies.

**Table 9:** Ensemble-Averaged Values of Normalized Nonbonded Intramolecular Interaction Energies between Nonpolar-Nonpolar Atoms Of Glucans^a^

		energy (kcal/mol) at different timesteps (ns)			
force field	glucans	301–350	351–400	401–450	451–500
CHARMM	Amylose	–4.95 ± 0.04	–4.89 ± 0.05	–4.89 ± 0.04	–4.92 ± 0.04
		0.47	0.58	0.52	0.43
	Dextran	–4.81 ± 0.04	–4.82 ± 0.03	–4.77 ± 0.05	–4.85 ± 0.02
		0.46	0.41	0.58	0.20
	Pullulan	–4.90 ± 0.03	–4.88 ± 0.02	–4.89 ± 0.02	–4.90 ± 0.03
		0.38	0.26	0.19	0.34
GLYCAM	Amylose	–12.91 ± 0.12	–12.85 ± 0.13	–12.81 ± 0.12	–12.77 ± 0.10
		0.55	0.57	0.53	0.44
	Dextran	–11.85 ± 0.04	–11.86 ± 0.03	–11.91 ± 0.03	–11.88 ± 0.10
		0.18	0.15	0.13	0.48
	Pullulan	–12.30 ± 0.06	–12.29 ± 0.10	–12.30 ± 0.06	–12.32 ± 0.07
		0.30	0.48	0.29	0.30
OPLS	Amylose	–12.91 ± 0.18	–12.86 ± 0.20	–12.88 ± 0.18	–12.86 ± 0.20
		0.80	0.90	0.79	0.88
	Dextran	–11.84 ± 0.04	–11.80 ± 0.03	–11.80 ± 0.04	–11.79 ± 0.06
		0.18	0.16	0.20	0.28
	Pullulan	–12.31 ± 0.04	–12.27 ± 0.09	–12.27 ± 0.09	–12.29 ± 0.03
		0.17	0.41	0.41	0.15

a The data is normalized by dividing the total energy by the number of respective atoms (390). The RSE is expressed as a percentage below the mean values.

**Table 10:** Ensemble-Averaged Values of Normalized Nonbonded Intramolecular Interaction Energies between Polar–Polar Atoms of Glucans^a^

		energy (kcal/mol) at different timesteps (ns)			
force field	glucans	301–350	351–400	401–450	451–500
CHARMM	Amylose	–1.06 ± 0.02	–1.03 ± 0.03	–1.03 ± 0.03	–1.04 ± 0.03
		1.11	1.54	1.60	1.58
	Dextran	–0.55 ± 0.04	–0.56 ± 0.03	–0.54 ± 0.03	–0.58 ± 0.02
		4.62	2.59	3.27	2.35
	Pullulan	–0.91 ± 0.03	–0.91 ± 0.02	–0.92 ± 0.00	–0.91 ± 0.02
		1.90	1.13	0.24	1.05
GLYCAM	Amylose	–1.81 ± 0.14	–1.80 ± 0.09	–1.78 ± 0.09	–1.75 ± 0.08
		4.34	2.76	2.97	2.53
	Dextran	–1.58 ± 0.10	–1.62 ± 0.12	–1.65 ± 0.09	–1.64 ± 0.22
		3.68	4.29	3.27	7.58
	Pullulan	–1.57 ± 0.06	–1.59 ± 0.09	–1.57 ± 0.05	–1.59 ± 0.07
		2.29	3.36	1.85	2.46
OPLS	Amylose	–1.60 ± 0.10	–1.52 ± 0.15	–1.58 ± 0.10	–1.57 ± 0.09
		3.71	5.72	3.48	3.40
	Dextran	–1.37 ± 0.06	–1.32 ± 0.12	–1.33 ± 0.15	–1.40 ± 0.15
		2.41	5.11	6.71	6.25
	Pullulan	–1.40 ± 0.05	–1.38 ± 0.07	–1.35 ± 0.10	–1.42 ± 0.04
		1.86	2.76	4.09	1.82

a The data is normalized by dividing the total energy by the number of respective atoms (243). The RSE is expressed as a percentage below the mean values.

GLYCAM and OPLS showed significantly stronger intramolecular nonpolar-nonpolar interactions compared to CHARMM for all glucans. The strongest (most negative) interactions were observed for amylose. This was surprising as amylose for three FFs showed expanded coil-like conformation. This apparent contradiction highlights that intramolecular nonpolar interactions alone do not dictate global chain collapse. Rather, overall conformation emerges from the combined contributions of polar–polar, nonpolar-nonpolar, and glucan-water interactions, modulated by the geometric constraints imposed by glycosidic linkage chemistry. Figure S27 and Table 9 suggest that the glucans show more hydrophobic (nonpolar) characteristics with GLYCAM and OPLS compared to CHARMM.

GLYCAM and OPLS consistently exhibited stronger intramolecular polar–polar interactions (more negative values) compared to CHARMM. The significantly stronger intramolecular polar–polar and nonpolar-nonpolar interaction energies predicted by GLYCAM and OPLS provide a clear energetic explanation for the more compact conformations of dextran and pullulan observed in these FFs.^14,21^ In contrast, the weaker intramolecular interactions in CHARMM reduce the energetic drive for chain collapse, allowing glucans to remain more extended and solvent-accessible. This analysis highlights that glucan–glucan interactions are as crucial as glucan-water interactions in shaping their conformations and solvation dynamics. Overall, the energetic decomposition reveals that in GLYCAM and OPLS, enhanced intramolecular stabilization outweighs the energetic benefit of solvent exposure for α-(1→6)-containing glucans, thereby promoting compaction, whereas in CHARMM, the weaker intramolecular stabilization preserves more extended conformations.

## 4 Conclusion

In this work, we employed extensive all-atom molecular dynamics (MD) simulations to elucidate how glycosidic linkage chemistry governs the structure, flexibility, and solvation of linear 30-mer α-glucans, dextran, amylose, and pullulan, in aqueous environments, through systematic comparison of the CHARMM, GLYCAM, and OPLS force fields (FFs). By integrating structural, vibrational, hydration, hydrogen-bonding, and energetic analyses, we resolved three central questions concerning linkage-dependent conformational behavior, proximal water organization, and the competition between glucan-water and intramolecular interactions.

We found that linkage topology strongly dictates accessible conformational space. The α-(1 → 4)-linked amylose adopted more extended conformations, reflected in higher *R*_g_ and *R*_h_ values and constrained dihedral distributions governed by the exoanomeric effect. In contrast, α-(1 → 6)-containing dextran and pullulan exhibited greater torsional flexibility. In GLYCAM and OPLS, this enhanced sampling enabled conformations that favored persistent intramolecular contacts, promoting chain compaction. These results demonstrate that subtle variations in glycosidic architecture significantly modulate glucan flexibility and structural persistence.

Hydration analyses (RDF, SASA, hydration-shell populations, and H-bond lifetimes) revealed that interfacial water structure is tightly coupled to chain conformation. Amylose maintained a solvent-exposed interface with dense but dynamic hydration, whereas dextran and pullulan exhibited reduced accessibility upon compaction. These linkage-dependent differences in hydration structure arise from the combined effects of glycosidic geometry and FF-dependent conformational sampling.

Energetic decomposition further clarifies this balance. GLYCAM and OPLS predicted systematically stronger intramolecular nonbonded stabilization, particularly for dextran and pullulan, which offsets favorable glucan-water interactions and drives compaction. In contrast, CHARMM exhibited weaker intramolecular stabilization and more dynamic H-bonding, preserving extended and solvent-accessible conformations. These results show that FF-dependent differences in intramolecular stabilization, rather than hydration strength alone, control whether α-(1 → 6)-containing glucans adopt compact or extended conformations under aqueous conditions.

For aqueous single-chain conformational and hydration-structure predictions of linear α-glucans under the present simulation conditions, our results favor CHARMM-TIP3P, as it preserves solvent-accessible conformations and differentiates linkage-dependent hydration without excessively stabilizing intramolecular contacts. In contrast, GLYCAM-TIP3P and OPLS-SPC/E predict systematically stronger intramolecular stabilization and consequently bias α-(1 → 6)-containing glucans toward more compact states. Overall, these findings guide FF selection and establish a mechanistic framework linking glycosidic architecture, conformational flexibility, hydration structure, and energetic competition, thereby informing the rational design of glycomaterials with tailored solubility and interfacial behavior.

## Supplementary Material





















## Data Availability

GROMACS topology and parameter files used to conduct all-atom molecular dynamics simulations, as well as several analysis codes used in this paper, are available to access using the following GitHub link: https://github.com/Deshmukh-Group/AA-FF-comparison-three-glucans. Any additional data from the current study are available from the corresponding author upon reasonable request.

## Notes

Tables of water model force field parameters and simulation data (*R*_g_, *R*_h_, dihedral angles, SASA, hydration, and hydrogen-bond), supporting figures (polymer conformations, structural evolution, dihedral angles, vibrational spectra, evolution of water at interface, hydrogen-bond autocorrelation function, and interaction energies), experimental methods and analysis for *R*_g_ and *R*_h_ measurements, detailed description of structural, dihedral angle, and interfacial solvent analyses (PDF)
Dynamic evolution of charmm-amylose (MP4)
Dynamic evolution of charmm-dextran (MP4)
Dynamic evolution of charmm-pullulan­(MP4)
Dynamic evolution of glycam-amylose (MP4)
Dynamic evolution of glycam-dextran (MP4)
Dynamic evolution of glycam-pullulan (MP4)
Dynamic evolution of opls-amylose (MP4)
Dynamic evolution of opls-dextran (MP4)
Dynamic evolution of opls-pullulan (MP4)
