# Supplementary material for: Atomistic Insights into Conformations and Solvation Dynamics of Amylose, Dextran, and Pullulan Using Three Force Fields
Source: J Phys Chem B. 2026 Apr 1;130(15):4207–23. doi: 10.1021/acs.jpcb.5c08146 (PMC13093480; doi:10.1021/acs.jpcb.5c08146)
Supplement: Supplementary file 1 [file jp5c08146_si_001.pdf]

Supporting Information

Atomistic Insights into Conformations and Solvation Dynamics of Amylose, Dextran, and Pullulan Using Three Force Fields

Parisa Farzeen[1], Hu Young Yoon[2], Isabela Trindade Coutinho[2], Maren Roman[2,4], Robert Moore[2,3], and Sanket A. Deshmukh[1]*

[1] Department of Chemical Engineering, Virginia Tech, Blacksburg, VA 24061, United States

[2] Macromolecules Innovation Institute, Virginia Tech, Blacksburg, VA 24061, United States

[3] Department of Chemistry, Virginia Tech, Blacksburg, VA 24061, United States

[4] Department of Sustainable Biomaterials, Virginia Tech, Blacksburg, VA 24061, United States

*Corresponding author

E-mail address: sanketad@vt.edu

Table S1: Parameters for different atomistic water models along with the carbohydrate FFs. Adapted with permission from ref 32. Copyright 2018 Taylor & Francis.

Parameter	Unit	TIP3P/CHARMM	TIP3P/GLYCAM	SPC/E/OPLS
r_{OH}	Å	0.9572	0.9572	1.0000
$\theta_{H\text{-}O\text{-}H}$	°	104.5200	-	109.4700
q_O	eV	-0.8340	-0.8340	-0.8476
q_H	eV	0.4170	0.4170	0.4238
$\sigma_{O\text{-}O}$	Å	3.1507	3.1507	3.1660
$\varepsilon_{O\text{-}O}$	kcal/mol	0.1521	0.1521	0.1553
$\sigma_{H\text{-}H}$	Å	0.4000	0.0000	0.0000
$\varepsilon_{H\text{-}H}$	kcal/mol	0.0460	0.0000	0.0000

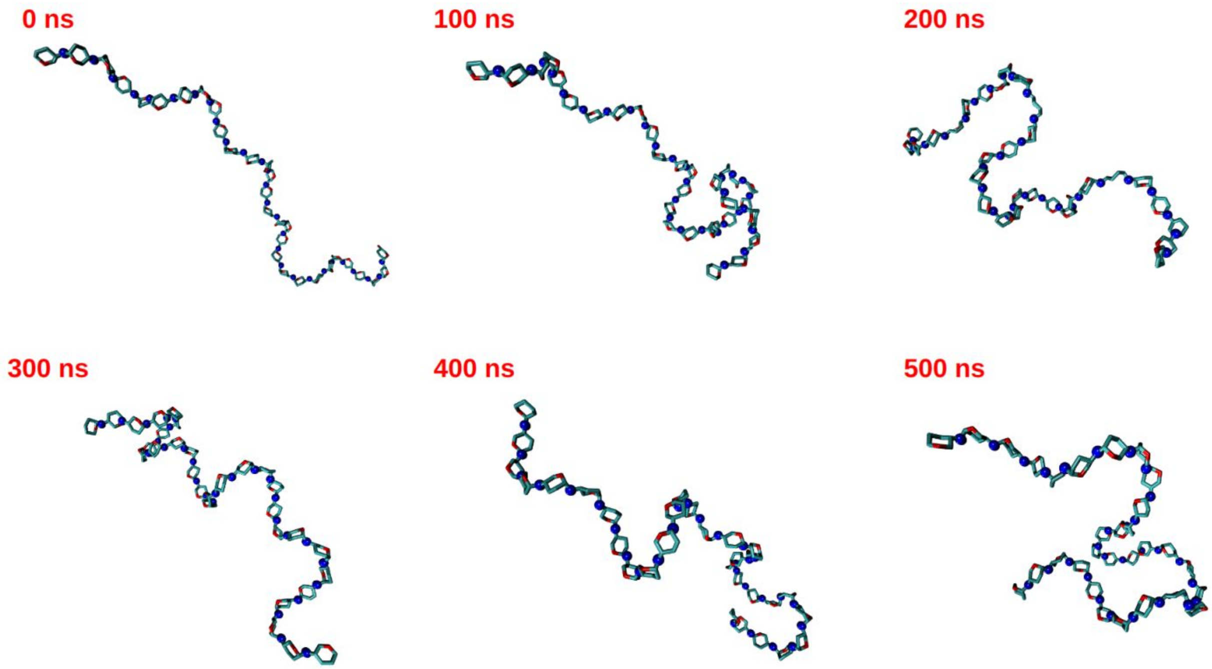

Figure S1: Simulation snapshots illustrating amylose conformations at the end of different timesteps (shown in red) performed with CHARMM FF. The carbon and oxygen atoms of the pyranose rings are shown in cyan and red, respectively, and glycosidic-linked oxygens are shown in blue, using the vdW style. Water and hydrogen atoms are not shown for clarity.

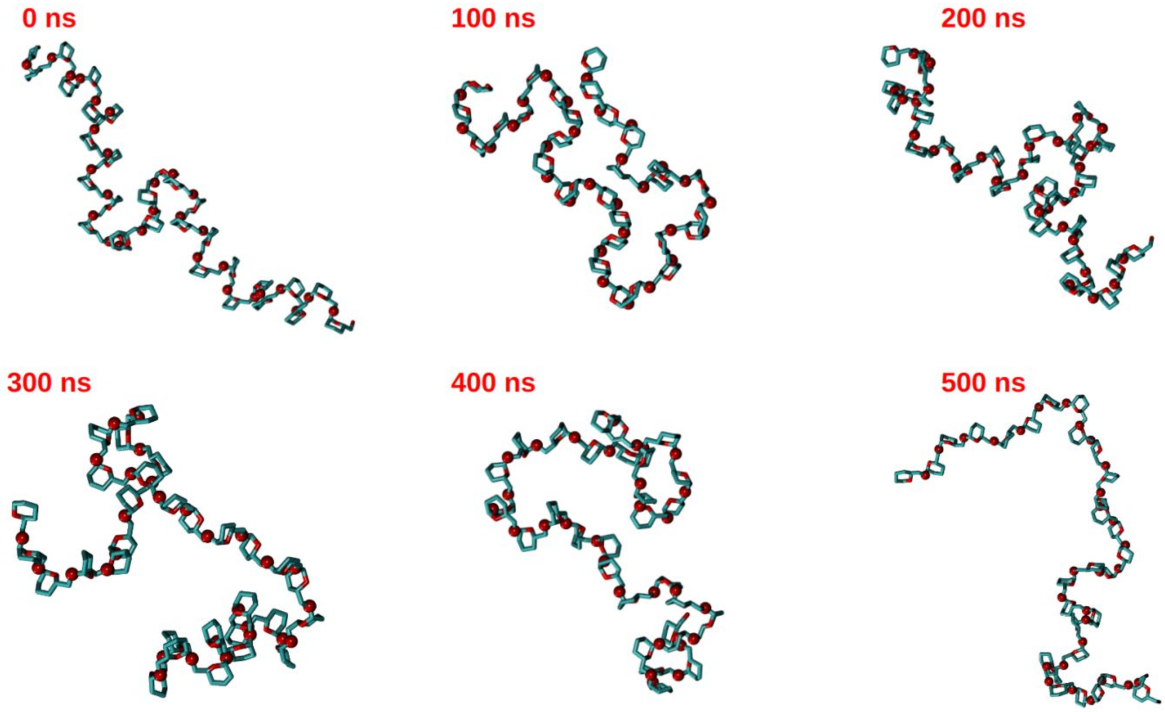

Figure S2: Simulation snapshots illustrating dextran conformations at the end of different timesteps (shown in red) performed with CHARMM FF. The carbon and oxygen atoms of the pyranose rings are shown in cyan and red, respectively, and glycosidic-linked oxygens are shown in red, using the vdW style. Water and hydrogen atoms are not shown for clarity.

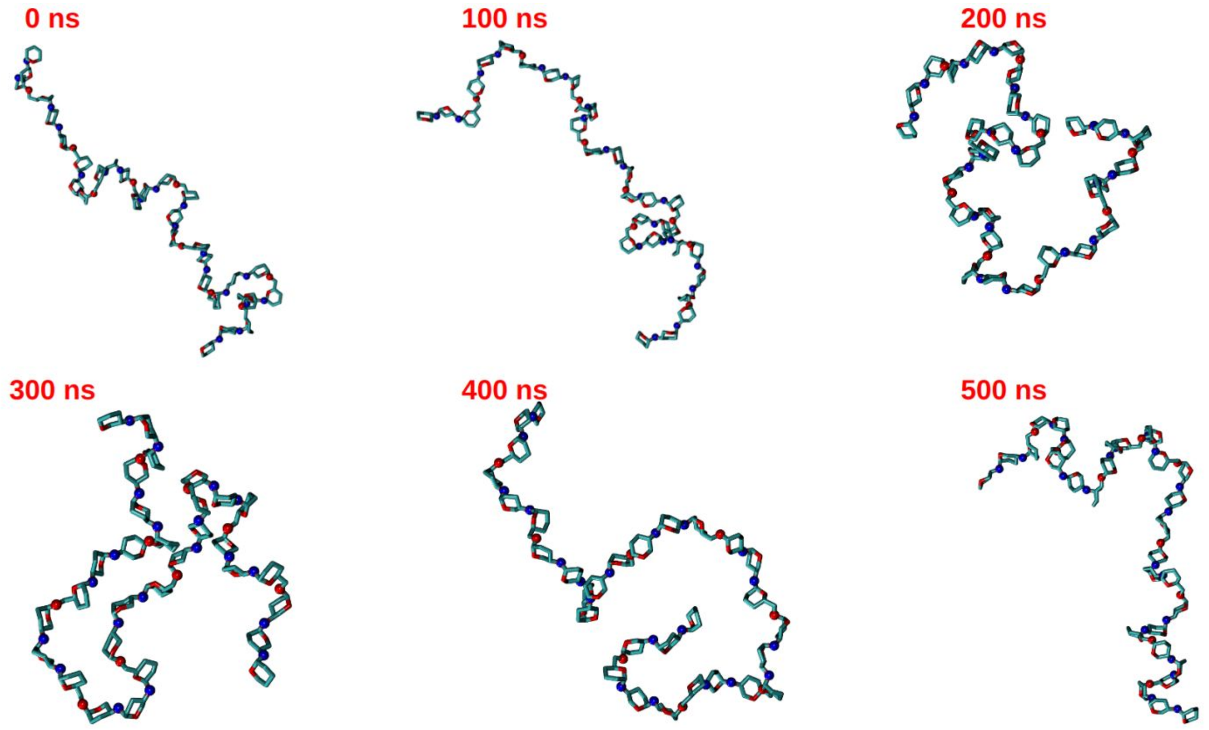

Figure S3: Simulation snapshots illustrating pullulan conformations at the end of different timesteps (shown in red) performed with CHARMM FF. The carbon and oxygen atoms of the pyranose rings are shown in cyan and red, respectively, and glycosidic-linked O4 and O6 atoms are shown in blue and red, respectively, using the vdW style. Water and hydrogen atoms are not shown for clarity.

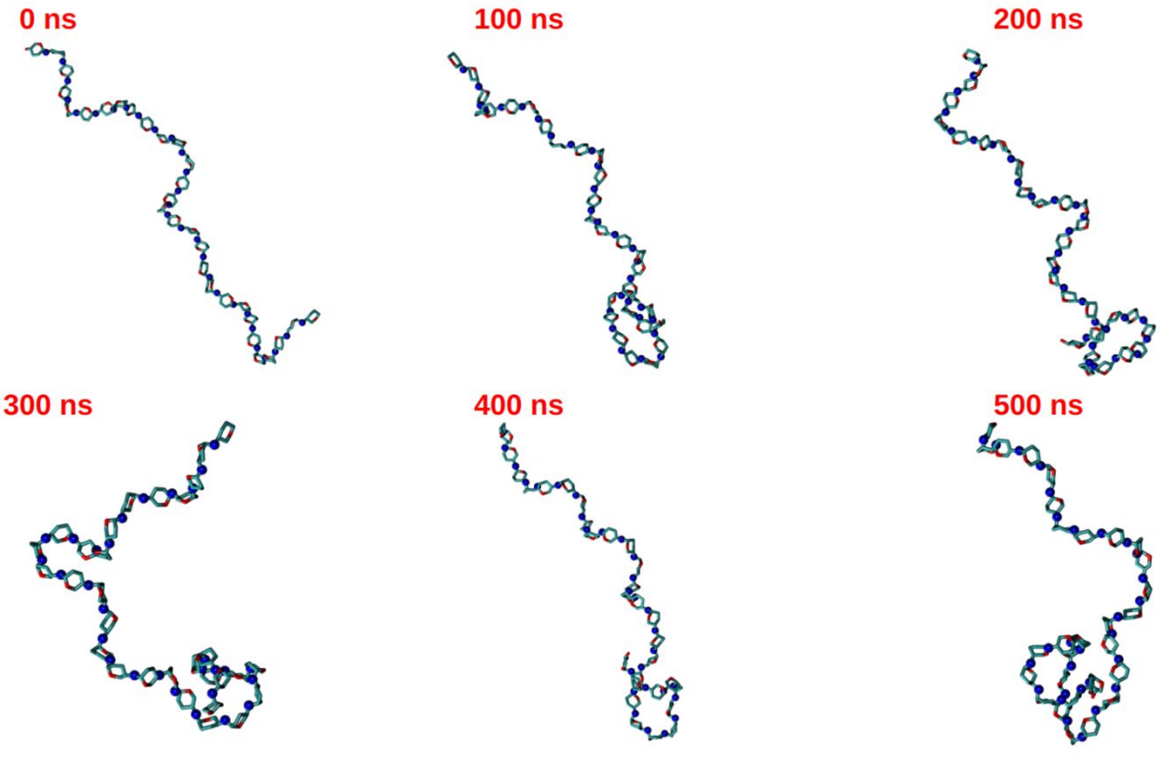

Figure S4: Simulation snapshots illustrating amylose conformations at the end of different timesteps (shown in red) performed with GLYCAM FF. The carbon and oxygen atoms of the pyranose rings are shown in cyan and red, respectively, and glycosidic-linked oxygens are shown in blue, using the vdW style. Water and hydrogen atoms are not shown for clarity.

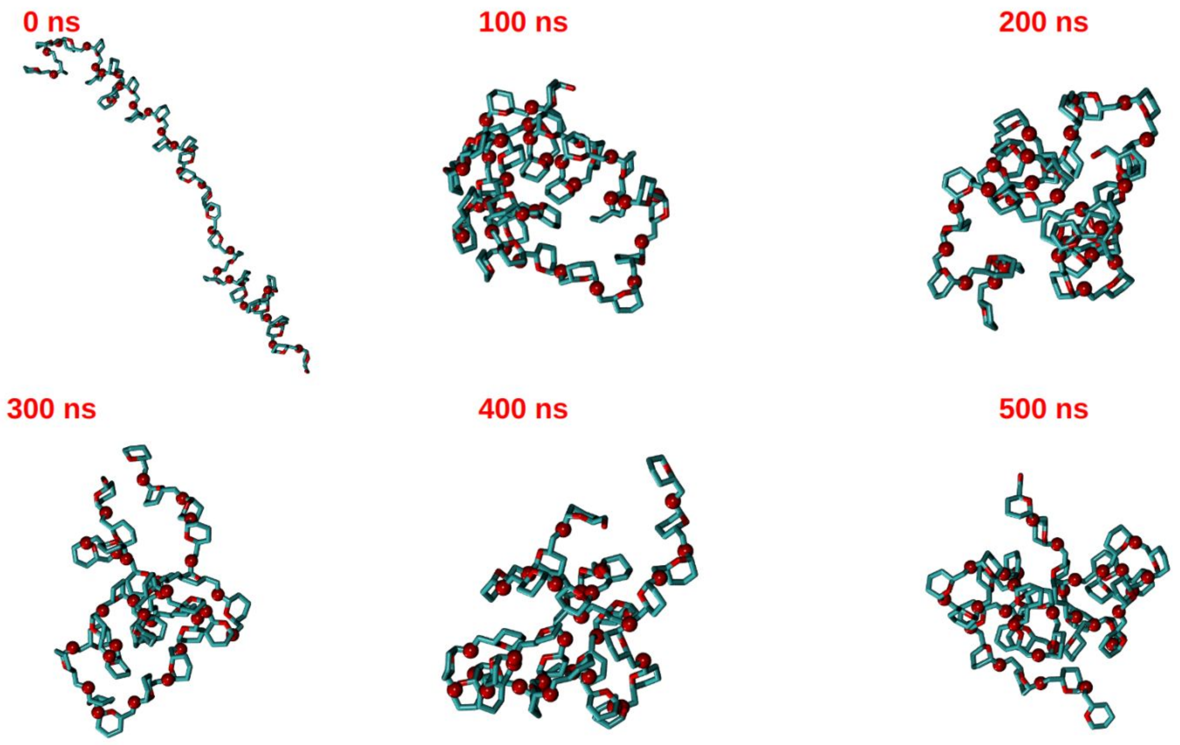

Figure S5: Simulation snapshots illustrating dextran conformations at the end of different timesteps (shown in red) performed with GLYCAM FF. The carbon and oxygen atoms of the pyranose rings are shown in cyan and red, respectively, and glycosidic-linked oxygens are shown in red, using the vdW style. Water and hydrogen atoms are not shown for clarity.

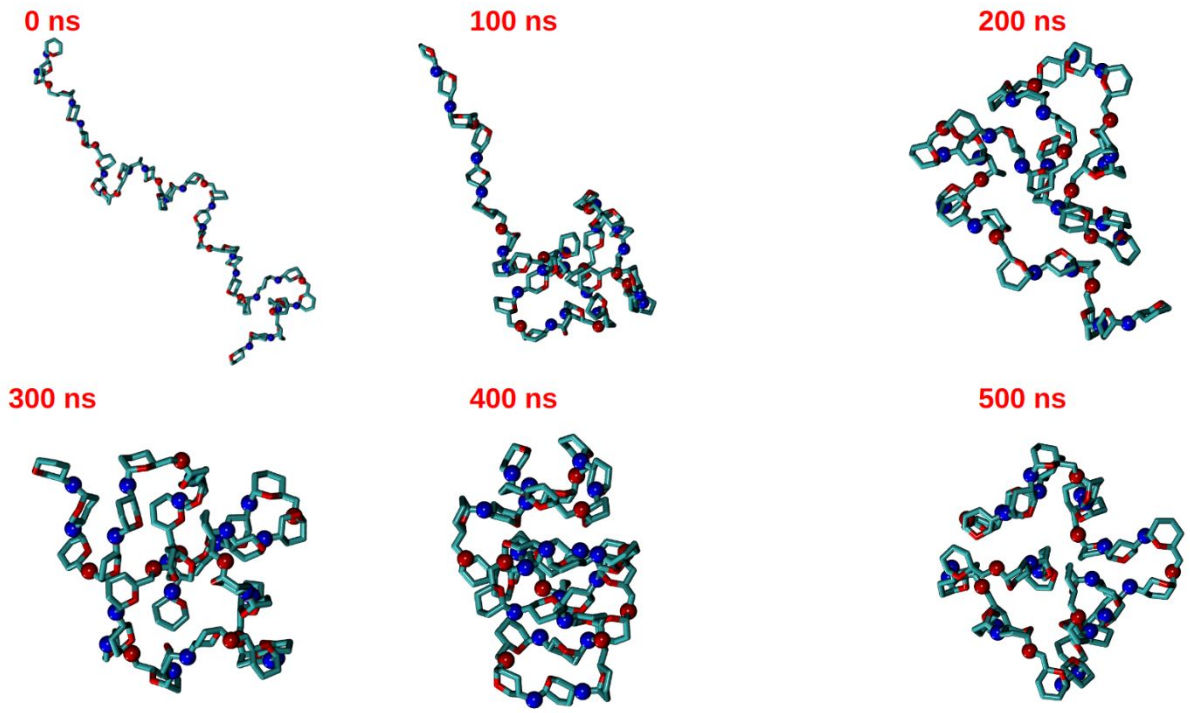

Figure S6: Simulation snapshots illustrating pullulan conformations at the end of different timesteps (shown in red) performed with GLYCAM FF. The carbon and oxygen atoms of the pyranose rings are shown in cyan and red, respectively, and glycosidic-linked O4 and O6 atoms are shown in blue and red, respectively, using the vdW style. Water and hydrogen atoms are not shown for clarity.

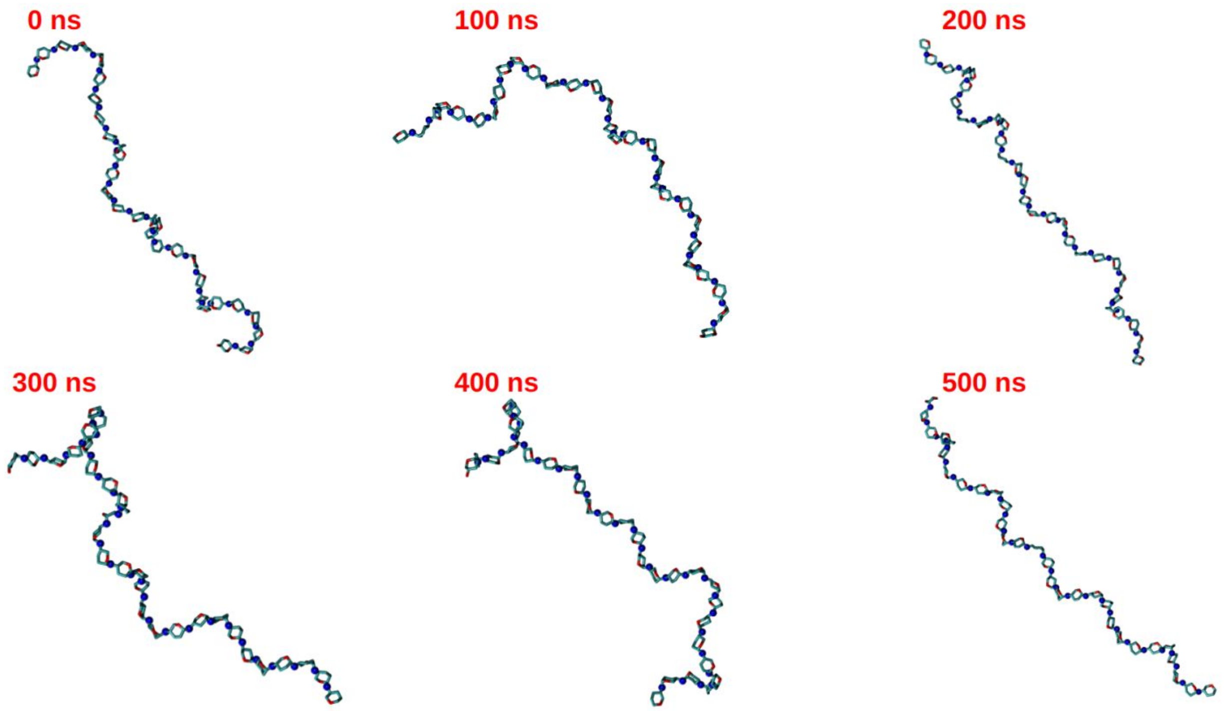

Figure S7: Simulation snapshots illustrating amylose conformations at the end of different timesteps (shown in red) performed with OPLS FF. The carbon and oxygen atoms of the pyranose rings are shown in cyan and red, respectively, and glycosidic-linked oxygens are shown in blue, using the vdW style. Water and hydrogen atoms are not shown for clarity.

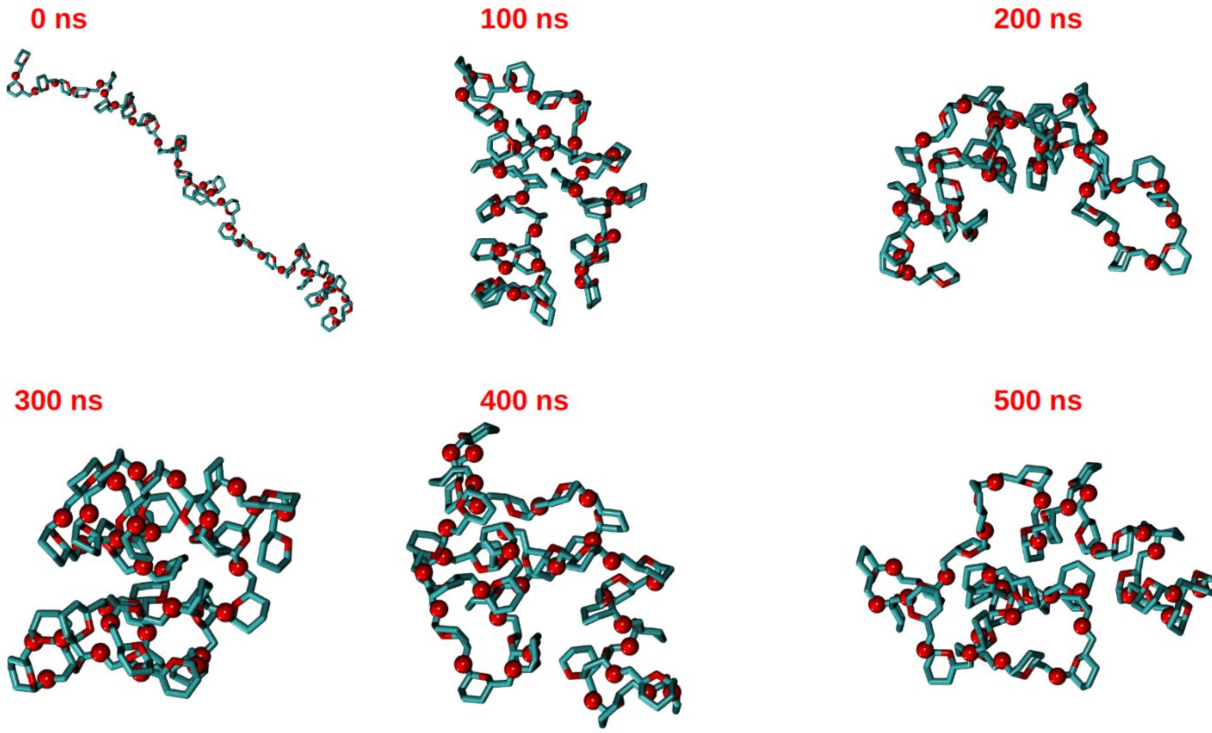

Figure S8: Simulation snapshots illustrating dextran conformations at the end of different timesteps (shown in red) performed with OPLS FF. The carbon and oxygen atoms of the pyranose rings are shown in cyan and red, respectively, and glycosidic-linked oxygens are shown in red, using the vdW style. Water and hydrogen atoms are not shown for clarity.

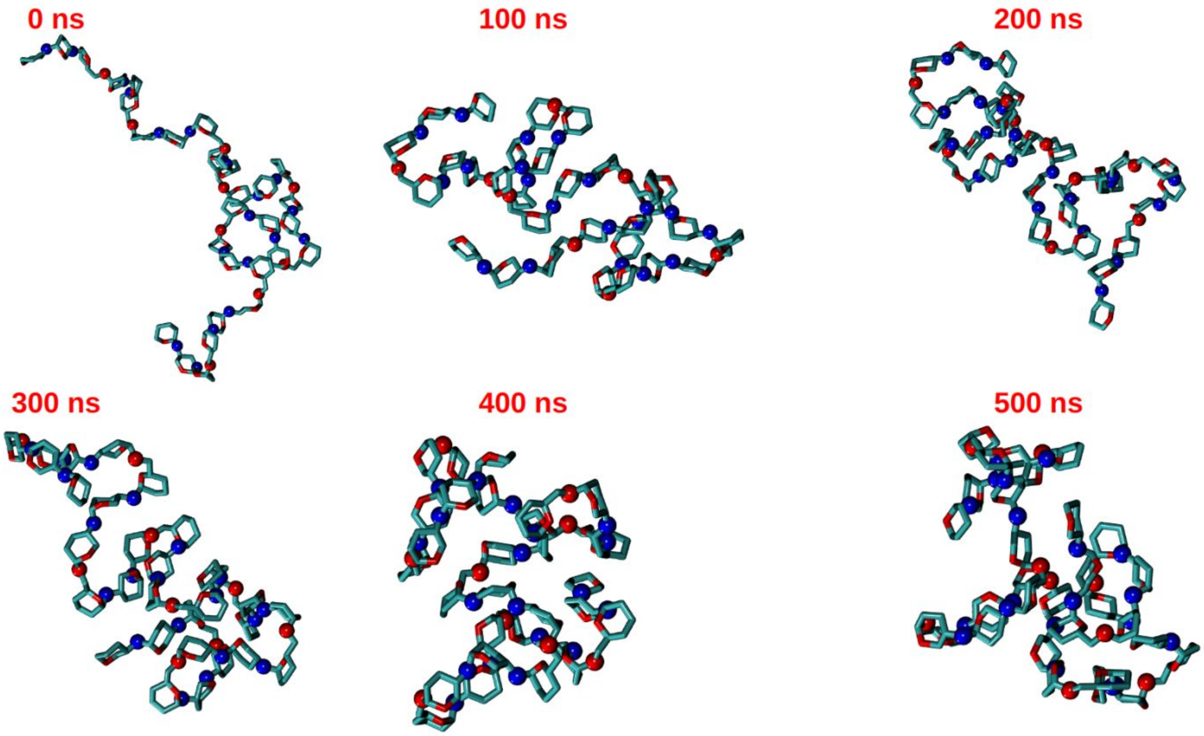

Figure S9: Simulation snapshots illustrating pullulan conformations at the end of different timesteps (shown in red) performed with OPLS FF. The carbon and oxygen atoms of the pyranose rings are shown in cyan and red, respectively, and glycosidic-linked O4 and O6 atoms are shown in blue and red, respectively, using the vdW style. Water and hydrogen atoms are not shown for clarity.

Section S1. Experimental R_h and R_g Determination

S1.1 Materials

Dextran 5 with a nominal molecular weight of 5,000 g/mol was purchased from Biosynth. Sodium chloride (NaCl) was obtained from Sigma-Aldrich, and deionized (DI) water from a Millipore Direct-Q 5 ultrapure water system.

S1.2 Sample Preparation

For dynamic light scattering (DLS) measurements, solutions were prepared by dissolving Dextran 5 in a 0.1 M NaCl solution to a final concentration of 0.5 mg/mL. The mixtures were stirred at 80°C overnight to ensure complete dissolution. After cooling, the solutions were filtered through a 0.22 μm PTFE syringe filter to remove particulates prior to the measurements. Note that NaCl used in the experimental setup was introduced primarily to suppress inter-chain interactions and aggregation at finite glucan concentrations.[1,2] In the MD simulations, a single isolated glucan chain was explicitly modeled, thereby inherently excluding inter-chain effects. Accordingly, pure water MD simulations provide a molecular-level representation that is conceptually consistent with the salt-stabilized single-chain conformations probed experimentally.

For small-angle X-ray scattering (SAXS) experiments, concentrated solutions of Dextran 5 were prepared by dissolving 100 mg/mL in a 0.1 M NaCl solution. These solutions were also stirred at 80°C overnight to ensure complete dissolution. After cooling, they were filtered through a 0.22 μm PTFE syringe filter and provided for SAXS analysis.

S1.3 R_h Measurements

The hydrodynamic radius (R_h) of Dextran 5 in solution was measured by DLS using a Malvern Panalytical Zetasizer Ultra. The instrument operated with a 633 nm laser, detecting scattered light at a 173° backscattering angle. Measurements were conducted at 25°C, and maintained using the Zetasizer's temperature control system. The refractive index and viscosity of water, pre-loaded into the software, were used as provided, with values of 1.33 and 0.8827 mPa·s, respectively. A refractive index of 1.331 and an absorption coefficient of 0.01 were chosen for the measurements.

Filtered Dextran 5 solutions (0.5 mg/mL), prepared as described above, were equilibrated at 25°C for 2 minutes prior to measurement. Each sample was measured three times, and the

results were averaged to determine the R_h. The averaged R_h values were reported along with standard deviations.

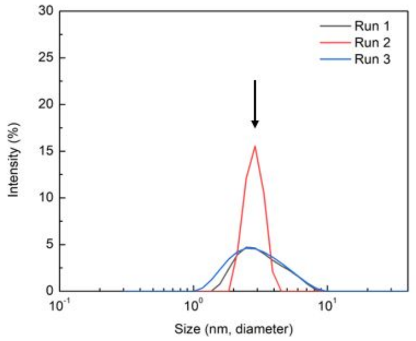

Figure S10: Experimental R_h distribution.

Table S2: The average size according to the peaks.

	Diameter (nm)		RH (nm)	
	**Mean**	**Std Dev**	**Mean**	**Std Dev**
Peak 1 Mean by Intensity ordered by size (nm)	2.62	0.23	1.31	0.12

S1.4 R_g Measurements

The radius of gyration (R_g) of dextran 5 was experimentally measured using small-angle and mid-angle X-ray scattering, SAXS, and MAXS, respectively. Concentrated solutions of dextran 5 were prepared by dissolving 100 mg/mL of dextran in a 0.1 M NaCl solution. These solutions were stirred at 80°C overnight to ensure complete dissolution. After cooling, the solutions were filtered through a 0.22 μm PTFE syringe and loaded into a quartz capillary (Hampton Research, California). SAXS and MAXS measurements were obtained using a capillary sample holder placed in an evacuated chamber in a Xenocs Xeuss 3.0 SAXS/WAXS system equipped with a GeniX 3D Cu HFVLF microfocus X-ray source utilizing Cu K-α radiation (λ =

0.154 nm). The sample-to-detector distances were 900 mm for SAXS and 370 mm for MAXS. The measurements were conducted using a high-resolution beam with exposure times of 2h for SAXS and 1h for MAXS. The two-dimensional patterns were obtained using a Dectris EIGER 4M detector, and the scattering vector (q) range was calibrated using silver behenate. The XSACT software was used to reduce the data to a one-dimensional form of azimuthally integrated intensity versus scattering vector (q). The data were normalized for background and absolute intensity, and the SAXS and MAXS profiles were merged into a single dataset. The software SasView was used for fitting the data into the monodisperse polymer gaussian coils model, described by the following equation:

$$I_{(q)} = scale \cdot I_0 \cdot P_{(q)} + background$$

Where,

$$I_0 = \varnothing_{poly} \cdot V \cdot (\rho_{poly} - \rho_{solv})^2$$

$$P_{(q)} = \frac{2[exp(-Z) + Z - 1}{Z^2}$$

$$Z = (qR_g)^2$$

$$V = M / N_A \delta$$

Where $\varnothing_{poly}$ is the volume fraction of polymer, V is the volume of a polymer coil, ρ_{poly} and ρ_{solv} are the scattering length densities of the polymer and the solvent, respectively, R_g is the radius of gyration, M is the molecular weight of the polymer, N_A is Avogadro's number, and δ is the bulk density of the polymer. The scaling factor and the background were fitted to the experimental data. SAXS and MAXS were used to obtain the experimental value of the radius of gyration for dextran 5. The monodisperse polymer Gaussian coils model was used as done in the

literature for polysaccharide solutions.[3] The radius of gyration obtained for Dextran 5 was 14.0 ± 1.0 Å.

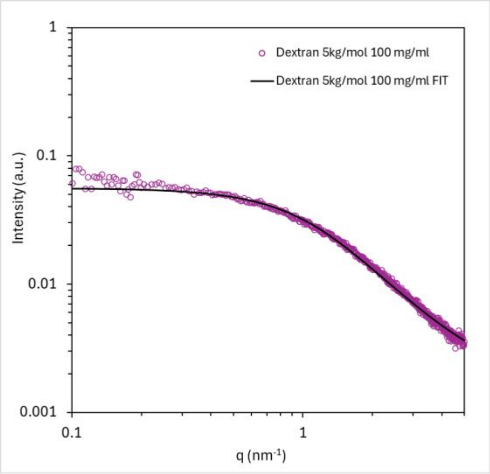

Figure S11: SAXS/MAXS scattering data and model fit for dextran 5.

Section S2. Structural Analysis of Glucans

As shown in **Eq. S1**, R_g is the square root of the sum of squared distances of the atoms from the center of mass (COM) of the polymer.

$$R_g^2 = \frac{1}{M}\sum_{i=1}^{N} m_i\,(r_i - R)^2 \text{...............}\textbf{Eq. S1}$$

Where M is the mass of the polymer chain, R is the COM of the polymer containing N atoms, and m_i and r_i are the mass and position of atom i, respectively.

R_h was calculated using the following **Eq. S2**.[4–6]

$$\frac{1}{R_h} = \frac{1}{N_{tot}(N_{tot}-1)} < \sum_{i\neq j} \frac{1}{r_{ij}} \text{...............}\textbf{Eq. S2}$$

Where N_{tot} is the total number of monomers in the glucan chain, and r_{ij} is the distance between monomer pairs i and j.

Molecular size and thermodynamic behavior can be calculated as a function of molecular weight (MW) for various macromolecules, including polymers, proteins, and glucans.[5,7] Generally, as the MW of a glucan increases, its radius of gyration (R_g) and the hydrodynamic radius (R_h) increase.[7–11] However, the quantitative correlation between MW and R_g or R_h depends on several factors, such as the techniques used for MW determination, the MW range studied, and branching in glycans. Studies have shown that by excluding anomalous data points, a power law relationship between MW and R_g or R_h for glucans and polymers can be established.[7,9,12] Here, a general power law relationship of the form presented by Fetters *et al.* [7] was used to determine R_g and R_h values using experimental data obtained from the literature. The R_g and R_h values obtained from MD simulations conducted with each FF and experimental data from the literature were compared. Note that the experimental data pertained to different MWs, and due to the lack of experimental data, we derived a general power law relationship that provides approximate R_g and R_h values for low molecular weight chains. Specifically, a general power law relationship of the form presented by Fetters *et al.* [7] was used:

$$P = C * MW^{v} \text{ ……………} \textbf{Eq. S3}$$

Here, P represents the property being probed as a function of MW, and C and v denote constants established empirically for the specific system, usually valid across several orders of magnitude in MW. Separate power laws to correlate the R_g and R_h of glucans to their respective MWs were generated. This correlation was used to predict R_g and R_h values for chains with 30-mers, which was the length of simulated glucans. Note that experimental data for the R_h of amylose were unavailable and are therefore not presented in this work.

S2.1 Relative Standard Error (RSE):

We have calculated the Relative Standard Error (RSE), which indicates how precise the ensemble mean estimate is, relative to its size.[13] The relative standard error (RSE) describes the

precision of the sample mean (from three independent runs) as an estimate of the population mean.[13,14] The RSE indicates how much the mean across independent runs fluctuates relative to its value, hence capturing sampling precision. RSE is calculated using the following equation,

$$RSE = \left(\frac{SE}{\mu}\right) \times 100\ \% \text{...............} \textbf{Eq. S4}$$

Where SE is the standard error, and μ is the ensemble mean. SE can be obtained using

$$SE = \frac{\sigma}{\sqrt{n}} \text{...............} \textbf{Eq. S5}$$

Where n = 3. We have calculated sample standard deviations from three runs (σ) and obtained SE. We obtained RSE for all temporal data, including R_g, R_h, SASA, the number of water molecules in the hydration shell, and interaction energies. 94.7% of the RSE values calculated fell within the 0-10% RSE range. 3.5% of the RSE were in the 10-20% range, and the remaining 1.8% of the data were within 20-25%. The low RSE values suggest that the samplings are relatively converged, and variability across independent runs did not dominate the mean estimates.

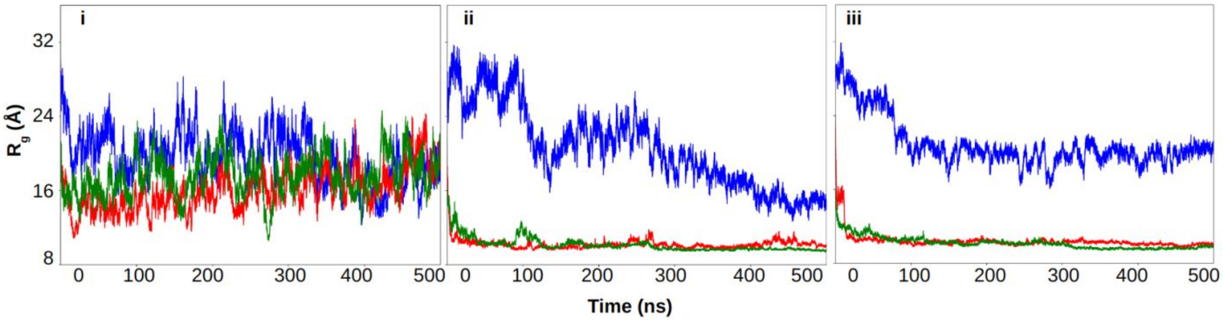

Figure S12: Time evolution of <R_g> of glucan chains amylose (blue), dextran (red), and pullulan (green) using **(i)** CHARMM, **(ii)** GLYCAM, and **(iii)** OPLS FFs.

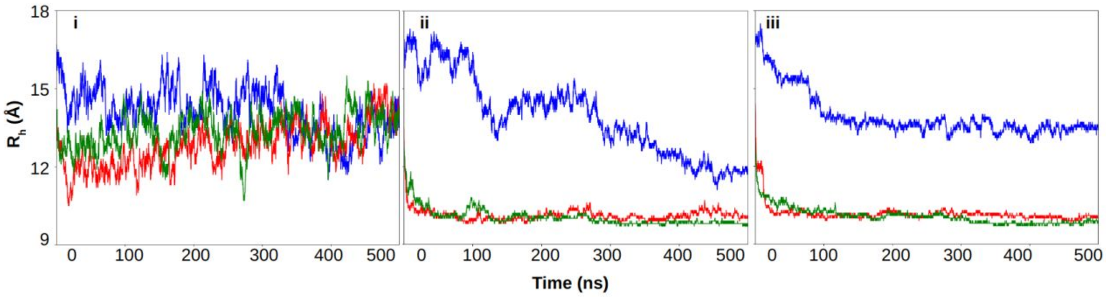

Figure S13: Time evolution of <R_h> of glucan chains amylose (blue), dextran (red), and pullulan (green) using **(i)** CHARMM, **(ii)** GLYCAM, and **(iii)** OPLS FFs.

Table S3: Ensemble-averaged values of the R_g at different timesteps (ns) for different glucan-water systems. The RSE is expressed as a percentage below the mean values.

		Ensemble-averaged R_g (Å) at different time steps (ns)			
**Force Field**	**Glucan**	**301-350**	**351-400**	**401-450**	**451-500**
CHARMM	Amylose	20.2±2.8	17.8±2.3	16.7±1.8	17.9±1.4
		8.03	7.38	6.08	4.46
	Dextran	16.9±1.6	17.1±1.8	16.0±1.9	19.6±0.4
		5.37	6.22	6.91	1.15
	Pullulan	18.6±3.2	17.1±0.6	18.4±1.8	18.8±2.2
		10.07	2.07	5.60	6.65
GLYCAM	Amylose	18.7±4.0	17.2±5.6	15.4±4.6	14.7±3.5
		12.32	18.85	17.17	13.75
	Dextran	10.0±0.1	9.9±0.3	10.4±0.5	10.3±0.3
		0.82	1.49	2.50	1.92
	Pullulan	9.6±0.4	9.7±0.5	9.7±0.4	9.6±0.4
		2.39	2.82	2.67	2.12
OPLS	Amylose	20.1±7.2	19.9±7.9	19.4±7.3	20.2±8.5
		20.78	22.88	21.75	24.16
	Dextran	10.4±0.1	10.2±0.1	10.1±0.2	10.0±0.2
		0.70	0.70	1.36	0.89
	Pullulan	9.7±0.1	9.6±0.2	9.6±0.3	9.7±0.1
		0.34	1.41	1.55	0.51

Table S4: Block averages of the R_g at different timesteps for different glucan-water systems at each run.

R_g (Å) at different time steps (ns)						
**Force Field**	**Glucan**	**Run**	**301-350**	**351-400**	**401-450**	**451-500**
CHARMM	Amylose	1	20.0±4.4	20.9±4.0	18.5±3.2	19.6±4.1
		2	23.7±3.6	15.6±4.6	14.3±1.7	16.2±2.6
		3	16.9±3.1	17.0±4.7	17.4±5.7	18.0±4.3
	Dxtran	1	17.0±2.4	14.9±2.5	15.5±3.2	20.2±2.9
		2	18.7±3.2	17.1±2.3	14.0±1.1	19.4±3.4
		3	14.9±2.6	19.4±3.1	18.6±2.9	19.3±3.2
	Pullulan	1	15.9±3.3	17.8±3.8	20.9±2.9	21.0±4.2
		2	16.8±3.6	17.1±2.5	17.1±2.3	15.9±2.0
		3	23.2±4.3	16.3±3.4	17.2±5.1	19.5±3.4
GLYCAM	Amylose	1	23.8±3.0	25.1±1.9	21.8±2.4	19.3±1.7
		2	14.0±0.3	13.7±0.6	12.7±0.7	13.7±0.7
		3	18.2±3.0	12.8±1.3	11.6±0.5	10.9±0.3
	Dextran	1	9.8±0.3	9.8±0.1	10.2±0.4	10.0±0.4
		2	10.1±0.2	9.7±0.2	10.0±0.3	10.1±0.2
		3	10.1±0.3	10.3±0.3	11.0±0.7	10.8±0.8
	Pullulan	1	9.1±0.1	9.0±0.2	9.1±0.1	9.1±0.1
		2	10.0±0.1	10.2±0.1	10.2±0.1	9.9±0.2
		3	9.8±01	9.8±0.1	9.8±0.1	9.8±0.1
OPLS	Amylose	1	17.6±0.5	17.1±0.8	16.3±0.6	16.3±0.3
		2	30.0±2.6	30.6±1.9	29.4±2.4	32.0±1.4
		3	12.7±0.4	11.9±0.4	12.4±0.6	12.4±0.6
	Dextran	1	10.3±0.3	10.1±0.3	9.8±0.2	10.1±0.2
		2	10.5±0.2	10.3±0.2	10.0±0.2	9.8±0.3
		3	10.3±0.2	10.3±0.2	10.4±0.2	10.1±0.1
	Pullulan	1	9.7±0.5	9.2±0.1	9.2±0.1	9.8±0.3
		2	9.8±0.2	9.8±0.1	9.8±0.2	9.6±0.1
		3	9.7±0.1	9.7±0.1	9.7±0.1	9.8±0.1

Table S5: 30-mer glucan R_g extrapolated from the respective power law, along with the MW range from reference literature. $\langle R_g \rangle$ over the last 200 ns of simulations, and the percentage error from three FFs is shown.

Glucan	Reference	MW range (kDa)	DP	General correlation	30-mer R_g using correlation [Theoretical Value] (Å)	$\langle R_g \rangle$ (Å) over last 200 ns (% error)		
						CHARMM	GLYCAM	OPLS
Amylose	[11]	80-350	500-2000	$0.0296MW^{0.530}$ (R^2=0.99)	26.7	18.1±1.1 (32.2)	16.4±4.3 (38.6)	19.9±7.7 (25.5)
	[15]	360-600	2000-3700					
Dextran	[8]	9-500	55-3100	$0.0299MW^{0.506}$ (R^2=0.88)	22.0	17.4±0.5 (20.9)	10.1±0.3 (54.1)	10.2±0.1(53.6)
	[8]	66-525	400-3200					
	[9]	80-400	500-2400					
Pullulan	[9]	300-510	1800-3000	$0.0171MW^{0.574}$ (R^2=0.98)	22.4	18.2±1.1 (18.8)	9.7±0.4 (56.7)	9.6±0.1 (57.1)
	[8]	100-980	600-6000					
	[10]	5-500	30-3000					
	[11,12]	47-833	290-5000					
	[16]	180-800	1000-5000					
Dextran[a]		~5	30		14.0±1.0	17.4±0.5 (24.3)	10.1±0.3 (27.9)	10.2±0.1(27.1)

[a] Experimental data from the current work

Table S6: Ensemble-averaged values of the R_h at different timesteps (ns) for different glucan-water systems. The RSE is expressed as a percentage below the mean values.

		Ensemble-averaged R_h (Å) at different time steps (ns)			
**Force Field**	**Glucan**	**301-350**	**351-400**	**401-450**	**451-500**
CHARMM	Amylose	16.5±1.3	15.2±1.3	14.7±0.9	15.5±0.8
		4.56	4.76	3.70	2.92
	Dextran	15.3±0.8	15.4±0.8	14.6±1.0	16.6±0.0
		2.97	3.11	3.81	0.16
	Pullulan	15.8±1.4	15.2±0.4	15.6±0.8	16.0±1.0
		5.28	1.47	3.05	3.57
GLYCAM	Amylose	15.1±2.0	14.4±2.5	13.6±2.0	13.2±1.4
		7.51	10.05	8.47	6.14
	Dextran	11.0±0.1	11.0±0.2	11.3±0.3	11.1±0.3
		0.50	0.86	1.69	1.36
	Pullulan	10.8±0.3	10.7±0.3	10.7±0.3	10.7±0.3
		1.54	1.77	1.76	1.55
OPLS	Amylose	15.6±3.4	15.3±3.6	15.3±3.4	15.5±3.9
		12.51	13.47	12.73	14.34
	Dextran	11.1±0.0	11.0±0.0	10.9±0.1	10.9±0.1
		0.00	0.25	0.66	0.66
	Pullulan	10.7±0.1	10.6±0.2	10.6±0.2	10.7±0.0
		0.44	1.18	1.18	0.26

Table S7: Block averages of the R_h at different timesteps for different glucan-water systems at each run.

			R_h (Å) at different time steps (ns)			
**Force Field**	**Glucan**	**Run**	**301-350**	**351-400**	**401-450**	**451-500**
CHARMM	Amylose	1	16.6±1.7	17.0±1.7	15.6±1.4	16.4±1.7
		2	18.1±1.1	14.2±2.1	13.4±0.7	14.5±1.3
		3	14.9±1.8	14.5±2.2	15.1±2.6	15.7±2.0
	Dxtran	1	15.1±1.1	14.3±1.3	14.3±1.4	16.6±1.2
		2	16.3±1.4	15.6±1.3	13.6±0.6	16.5±1.3
		3	14.4±1.3	16.3±1.4	15.9±1.3	16.6±1.2
	Pullulan	1	14.6±1.4	15.7±1.6	16.8±1.2	16.9±1.5
		2	14.9±1.7	15.0±1.3	15.0±1.1	14.6±0.9
		3	17.8±1.5	14.8±1.6	15.1±2.3	16.4±1.3
GLYCAM	Amylose	1	17.6±1.0	17.9±0.6	16.4±0.9	15.0±0.5
		2	12.8±0.1	12.7±0.2	12.5±0.5	12.9±0.3
		3	14.9±1.2	12.5±0.7	11.9±0.2	11.6±0.2
	Dextran	1	10.9±0.3	11.0±0.1	11.2±0.3	11.0±0.2
		2	10.9±0.1	10.8±0.1	10.9±0.2	10.9±0.1
		3	11.1±0.1	11.1±0.1	11.1±0.1	11.0±0.1
	Pullulan	1	10.4±0.1	10.3±0.2	10.3±0.1	10.3±0.1
		2	11.1±0.1	11.1±0.1	11.1±0.1	11.0±0.1
		3	10.8±0.1	10.8±0.1	10.7±0.2	10.7±0.1
OPLS	Amylose	1	14.6±0.3	14.1±0.4	13.9±0.3	13.7±0.1
		2	20.2±0.8	20.2±0.9	20.0±0.8	20.9±0.3
		3	12.1±0.2	11.7±0.2	12.1±0.3	12.0±0.3
	Dextran	1	11.1±0.1	10.9±0.2	10.8±0.1	10.9±0.1
		2	11.1±0.1	11.0±0.1	10.9±0.2	10.7±0.2
		3	11.1±0.1	11.0±0.1	11.1±0.1	11.0±0.1
	Pullulan	1	10.6±0.3	10.3±0.1	10.3±0.1	10.7±0.2
		2	10.8±0.1	10.8±0.1	10.8±0.1	10.6±0.1
		3	10.7±0.1	10.7±0.1	10.7±0.1	10.7±0.1

Table S8: 30-mer glucan R_h extrapolated from the respective power law, along with the MW range from reference literature. <R_h> over the last 200 ns of simulations, and the percentage error from three FFs is shown.

Glucan	References	MW range (kDa)	DP	General correlation	30-mer R_h using correlation [Theoretical Value] (Å)	<R_h> (Å) over last 200 ns (% error)		
						CHARMM	GLYCAM	OPLS
Dextran	[8]	9-500	55-3100	$0.0396MW^{0.478}$ (R2=0.92)	23.0	15.5±0.3 (32.6)	11.1±0.2 (51.7)	10.9±0.1 (52.6)
	[8]	66-520	400-3200					
	[9]	80-400	500-2400					
	Our experiments	5	30					
Pullulan	[9]	300-500	1800-3000	$0.0344MW^{0.474}$ (R2=0.99)	19.3	15.6±0.6 (19.2)	10.7±0.3 (44.6)	10.6±0.1 (45.1)
	[10]	5-500	30-3000					
Dextran[a]	Our experiments	~5	~30	-	13.1±1.2	15.5±0.3 (18.3)	11.1±0.2 (15.3)	10.9±0.1 (16.8)

[a] Experimental data from the current work

S2.2 Dihedral Angle Calculation

Gerbst *et. al.* have reported that the glycosidic linkage conformation is an important factor in determining the overall shape and flexibility of the carbohydrate molecules and their interactions with other biomolecules and solvents.[17] The relative orientation of two monosaccharides around

the glycosidic bond can be affected by the glycosidic linkage dihedral angles ϕ (O_5'-C_1'-O_6-C_6 for dextran and O_5'-C_1'-O_4-C_4 for amylose) and ψ (C_1'-O_6-C_6-C_5 for dextran and C_1'-O_4-C_4-C_3 for amylose), shown in **Figure S14**.[18] For monomers linked with α-(1→6) linkage similar to those in dextran, additional flexibility exists in the glucan chain due to the dihedral angle ω (O_6-C_6-C_5-O_5). All three dihedral angles can be found in pullulan due to the existence of α-(1→6) and α-(1→4) linkages. The ϕ angle is strongly influenced by the exo-anomeric effect, which is related to the stereo electronic impact caused by the polarity of the substituent and the lone electron pair on the linkage oxygen.[19] The ψ angle is affected by steric effects and hydrogen bonds between glucan and glucan and between glucan and water.[19]

Dihedral angle distributions were computed over the last 200 ns of each trajectory to ensure equilibrated sampling and to enable statistically robust comparison of conformational preferences across glucans and FFs (**Table S9**). We calculated the mean angle at each timestep across the three datasets, yielding an average dataset, which is shown in **Table 2**. The reported angle values represent the averaged response, along with standard deviations, from three independent trajectories. Using the angle data, we determined the relative populations of conformers by examining how much of the angle data falls into the ranges associated with each rotamer or conformation.[20] **Table 2** also shows the comparison of the most probable glycosidic-linkage dihedral angle values for the three glucans for three FFs with available experimental data. **Figure S15** shows the corresponding normalized probability distribution for the glycosidic dihedral angles ϕ, ψ, and ω. In general, the MD simulation results are in reasonable agreement with available experimental data from the literature.

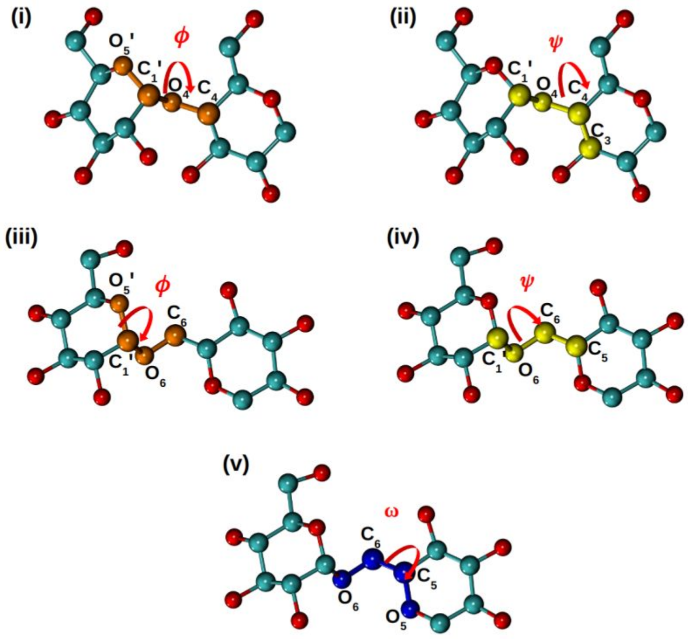

Figure S14: Dihedral angles (i), (iii) ϕ shown in orange and (ii), (iv) ψ shown in yellow for α-(1→4) linked and α-(1→6) D-glucans, respectively, and dihedral angle (v) ω shown in blue for α-(1→6) linked D-glucans.

Table S9: Most probable angle values of the glycosidic linkage torsion angles from each run over the last 200 ns. The percentage population of angles observed in the given range is shown in parentheses. Angles were obtained in the interval from -180° to 180°.

Force field	Glucan link	Run	ϕ		ψ			ω		
			ϕexo	anti-ϕ	ψ180	ψ90	ψ-90	gg	gt	tg
CHARMM	Amylose 1-4	1	89 (100)	-	-	91 (100)	-	-	-	-
		2	91 (100)	-	-	88 (100)	-	-	-	-
		3	89 (100)	-	-	87 (100)	-	-	-	-
	Dextran 1-6	1	69 (100)	-	-	92 (28)	-20 (72)	-7 (43)	22 (57)	-
		2	69 (100)	-	-	99 (21)	-18 (79)	-26, -13 (100)	-	-
		3	69 (100)	-	-	99 (28)	-16 (72)	-9 (74)	8 (26)	-
	Pullulan 1-4	1	90 (100)	-	-	86 (100)	-	-	-	-
		2	91 (100)	-	-	90 (100)	-	-	-	-
		3	90 (100)	-	-	94 (100)	-	-	-	-
	Pullulan 1-6	1	70 (100)	-	-169 (5)	17, 90 (34)	-94, -19 (61)	-8 (67)	19 (33)	-
		2	70 (100)	-	-168 (5)	17, 88 (32)	-94, -19 (63)	-20 (8)	6 (92)	-
		3	70 (100)	-	-168 (6)	18, 92 (31)	-93, -19 (63)	-8 (87)	5 (13)	-
GLYCAM	Amylose 1-4	1	78 (100)	-	-	86 (100)	-	-	-	-
		2	79 (100)	-	-	75, 86 (100)	-	-	-	-
		3	83 (100)	-	-	92 (100)	-	-	-	-
	Dextran 1-6	1	64 (100)	-	-	42 (4)	-83, -60 (96)	-	36 (100)	-
		2	64 (100)	-	-	44 (1)	-84, -60 (99)	-	29 (100)	-
		3	63 (100)	-	-	49 (3)	-88, -65 (97)	-	31 (100)	-
	Pullulan 1-4	1	77 (100)	-	-	75 (100)	-	-	-	-
		2	73 (100)	-	-	71 (100)	-	-	-	-
		3	78 (100)	-	-	78 (100)	-	-	-	-

	Pullulan 1-6	1	75 (100)	-	-150 (39)	-	-111, 73 (61)	-	69 (100)	-
		2	71 (100)	-	-162 (12)	-	-90, -54 (88)	-30 (3)	46 (97)	-
		3	74 (100)	-	-	20 (7)	-52 (93)	-	63 (100)	-
OPLS	Amylose 1-4	1	75 (100)	-	-	82 (100)	-	-	-	-
		2	75 (100)	-	-	89 (100)	-	-	-	-
		3	74, 86 (100)	-	-	91 (100)	-	-	-	-
	Dextran 1-6	1	71 (100)	-	-	-	-67, -45 (100)	-	45 (100)	-
		2	72 (100)	-	-	-	-70, -48 (100)	-	52 (100)	-
		3	72 (100)	-	-	-	-71, -46 (100)	-	44 (100)	-
	Pullulan 1-4	1	78 (100)	-	-	94 (100)	-	-	-	-
		2	76 (100)	-	-	82 (100)	-	-	-	-
		3	80 (100)	-	-	54, 71 (100)	-	-	-	-
	Pullulan 1-6	1	74 (100)	-	-150 (25)	-	-76, -50 (75)	-	89 (100)	-
		2	73 (100)	-	-157 (17)	56 (1)	-82, -45 (82)	-	70 (100)	-
		3	72 (100)	-	-	31 (15)	-82, -45 (85)	-	93 (100)	-

ϕ_{exo} : 0° < ϕ < 120°, ψ_{180}: 120° < ψ < 180° and -180° < ψ < -120°, ψ_{90}: 0° < ψ < 120°, ψ_{-90}: -120° < ψ < 0°, ω_{gg}: -120° < ω < 0°, ω_{gt}: 0° < ω < 120°, ω_{tg}: -180° < ω < -120° and 120° < ω < 180°

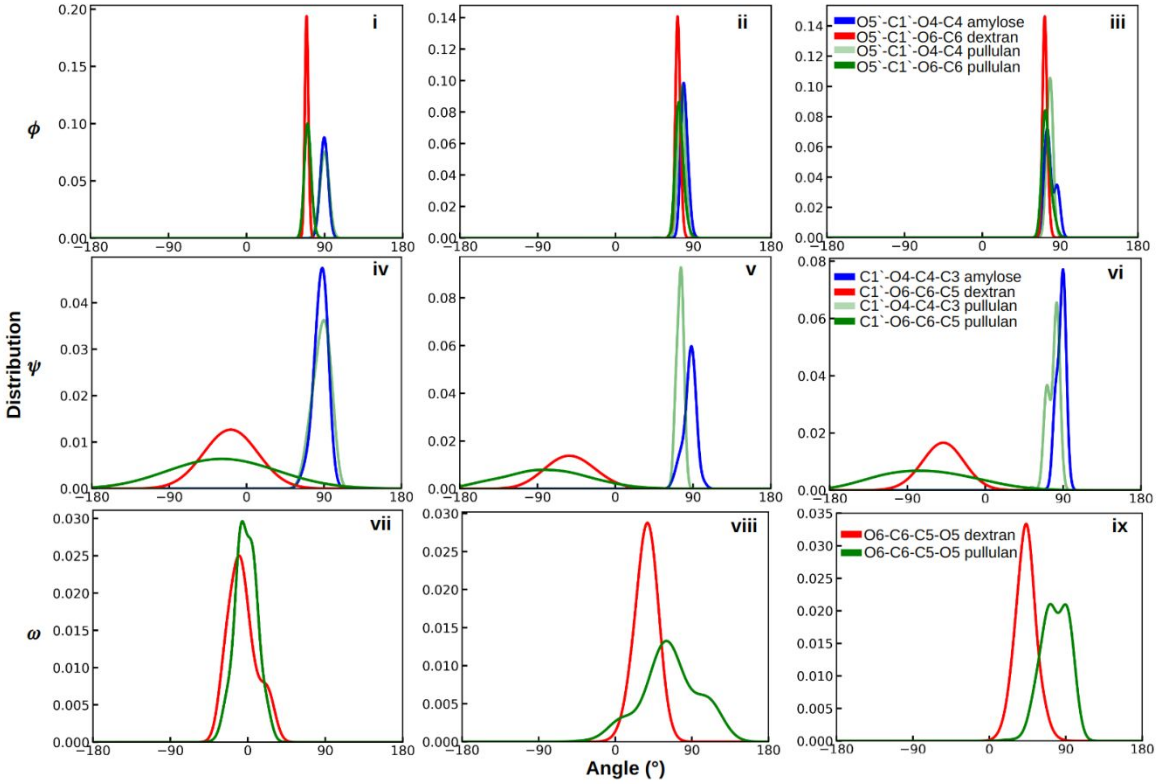

Figure S15: Dihedral angle distribution of glycosidic-linked atoms. Dihedral angles ϕ **(i)** CHARMM, **(ii)** GLYCAM, and **(iii)** OPLS. Dihedral angles ψ **(iv)** CHARMM, **(v)** GLYCAM, and **(vi)** OPLS. And dihedral angles ω **(vii)** CHARMM, **(viii)** GLYCAM, and **(ix)** OPLS.

S2.3 Vibrational Spectra Calculation

The vibrational spectrum derived from MD simulation trajectories can provide valuable insights into the molecular-level structures of glucans.[21,22] Here, we calculated the vibrational spectra of glycosidic carbons, C_1, C_4, and C_6, shown in **Figure 1**, to evaluate the ability of CHARMM, GLYCAM, and OPLS FFs to predict these spectra. Note, these were obtained from the extended 50 ps simulations conducted with non-rigid water. We calculated IR spectra for each run of a particular glucan-FF system, and observed that they showed consistent peak positions across all runs, indicating good reproducibility (**Figures S16** to **S18**). Therefore, we combined the spectra by calculating the mean intensity at each frequency across the three datasets, yielding

an averaged spectrum. The reported peak positions, which correspond to different frequencies, are listed in **Tables 3, 4,** and **5**. These represent the averaged response, along with standard deviations, from three independent trajectories.

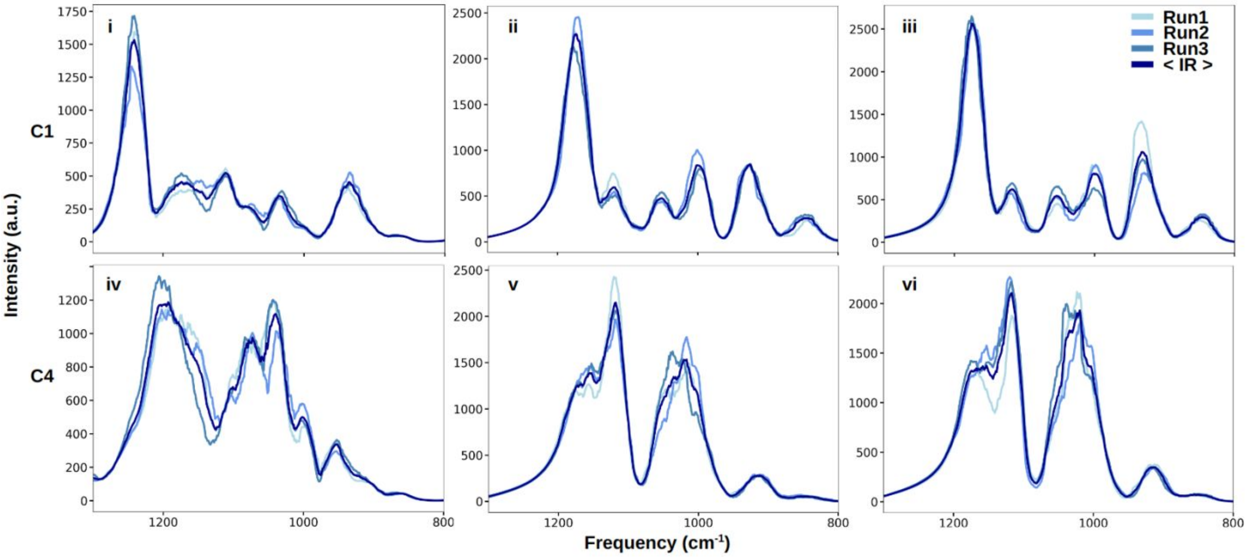

Figure S16: Vibrational spectra of C1 and C4 atoms of amylose using (i, iv) CHARMM, (ii, v) GLYCAM, and (iii, vi) OPLS FFs, showing 800-1300 cm^{-1} region. Individual runs are shown in increasingly darker shades, with ensemble average, <IR>, shown in the darkest shade.

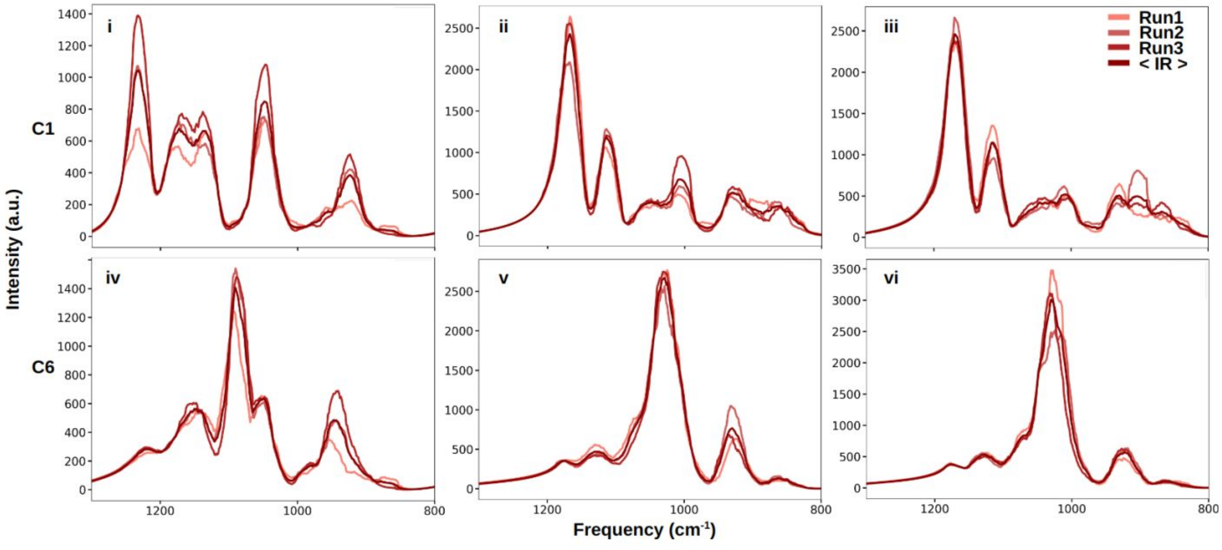

Figure S17: Vibrational spectra of C1 and C6 atoms of dextran using (i, iv) CHARMM, (ii, v) GLYCAM, and (iii, vi) OPLS FFs, showing 800-1300 cm^{-1} region. Individual runs are shown in increasingly darker shades, with ensemble average, <IR>, shown in the darkest shade.

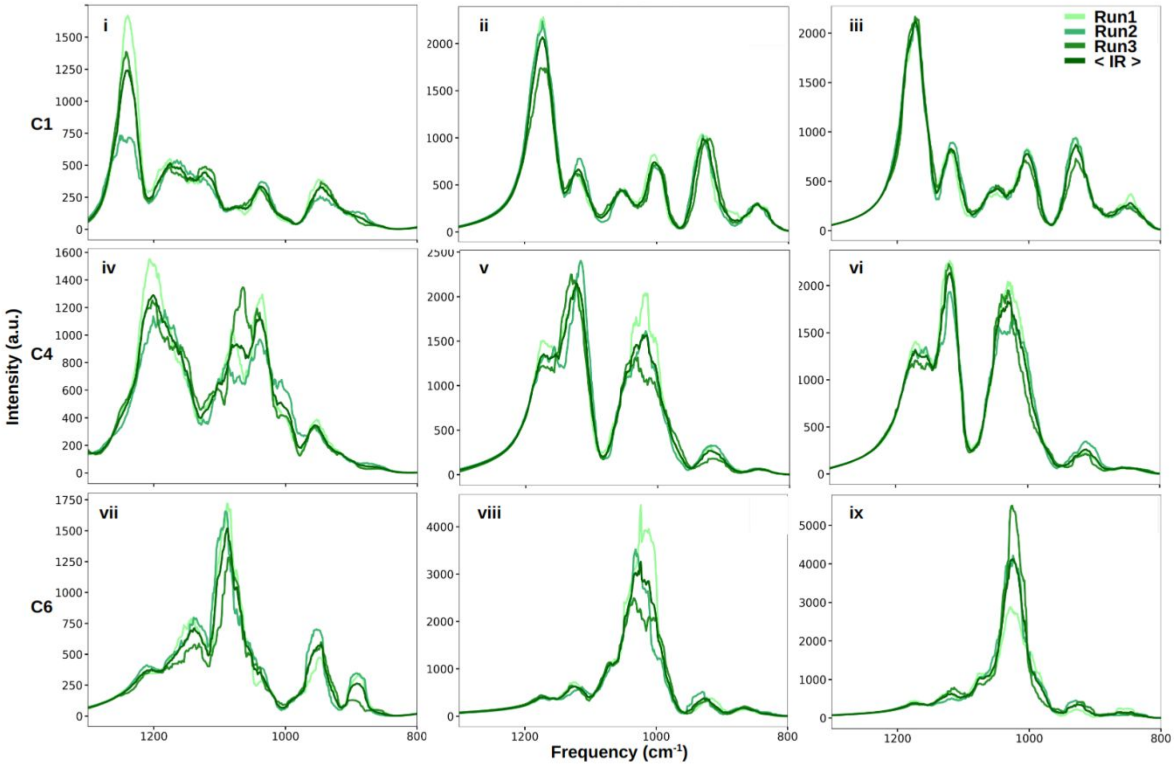

Figure S18: Vibrational spectra of C1, C4, and C6 atoms of pullulan using (i, iv, vii) CHARMM, (ii, v, viii) GLYCAM, and (iii, vi, ix) OPLS FFs, showing 800-1300 cm^{-1} region. Individual runs are shown in increasingly darker shades, with ensemble average, <IR>, shown in the darkest shade.

Section S3 Structure of Interfacial Solvent

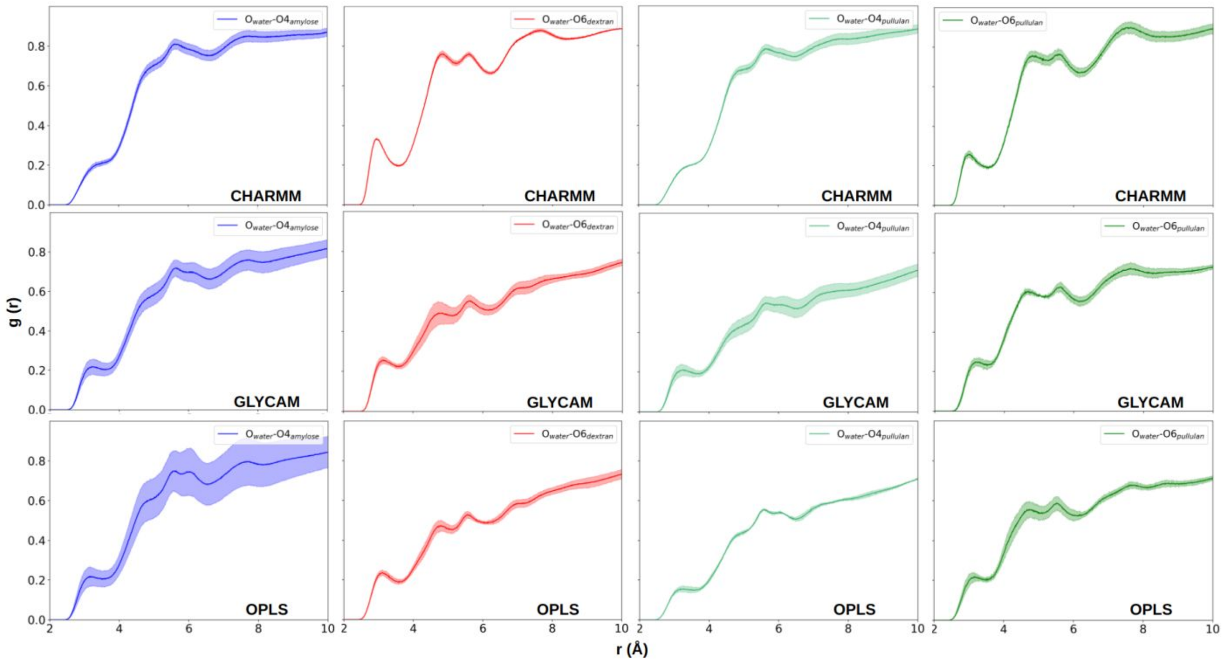

Figure S19: RDF between water-oxygens and glycosidic oxygens O_4 in α-(1➡4) linked amylose (blue), O_6 in α-(1➡6) linked dextran (red), O_4 in α-(1➡4) linked pullulan (light green), O_6 in α-(1➡6) linked pullulan (dark green) for CHARMM, GLYCAM, and OPLS FFs. The shaded region indicates standard deviation.

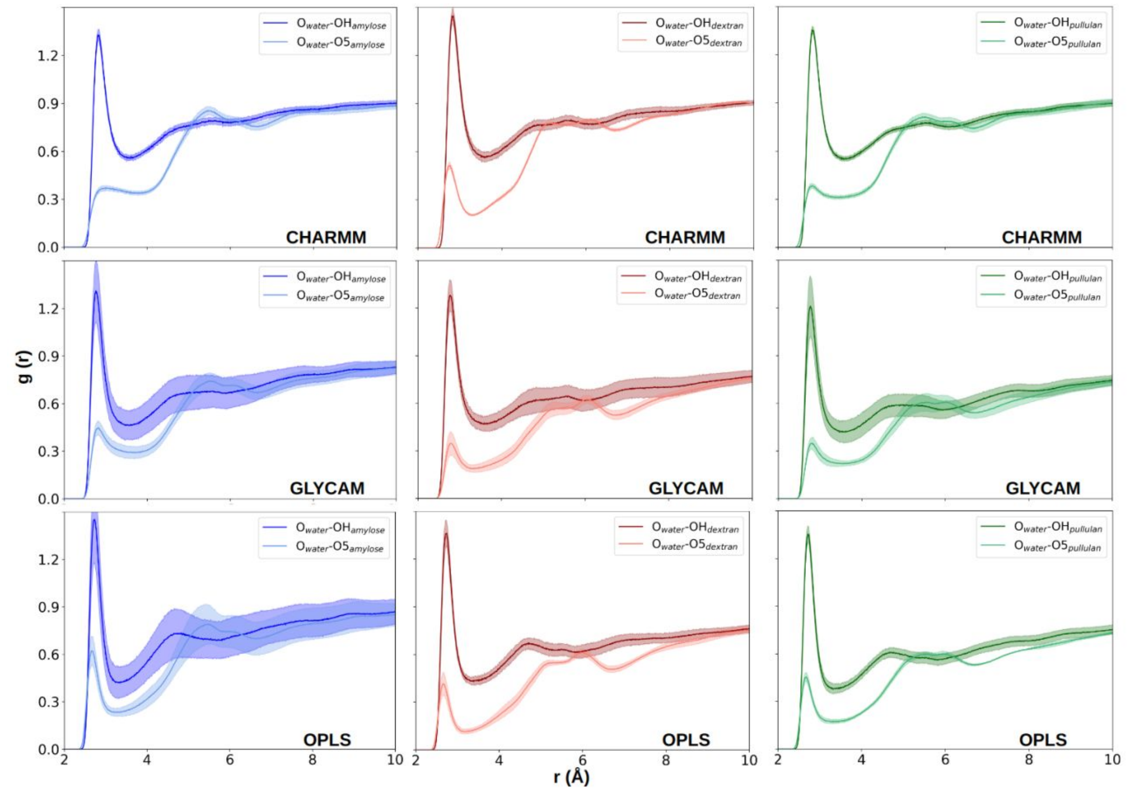

Figure S20: RDF between water-oxygen (OW) and -OH groups and ring-oxygen (O_5) for amylose (blue), dextran (red), and pullulan (green) for CHARMM, GLYCAM, and OPLS FFs. The shaded region indicates standard deviation.

S3.1 Solvent Accessible Surface Area

To characterize the glucan-water interface, we quantified the solvent accessible surface area (SASA) of each glucan chain. SASA provides a direct measure of the extent to which a polymer surface is exposed to surrounding solvent molecules.[23–25] SASA is a measure of the surface area of the molecule that is accessible by the solvent molecules and is measured by employing a probe, usually a sphere, to sketch out the molecular surface as a series of dots. Therefore, the more extended the glucan chain is, the higher the SASA, which can imply higher exposure of the glucan to water.[25] In MD simulations, this accessible surface is defined as the van der Waals covering of the glucan molecule plus the radius of the solvent sphere from each

solute atom center.[24] Following the double cubic lattice method (DCLM) algorithm[26] with a probe size of 1.4 Å, we calculated SASA for the three glucans. The total time evolution plots of SASA are shown in **Figure S21**. The ensemble average values of SASA at different time intervals and the block average SASA for each independent run are reported in **Table S10** and **Table S11**, respectively.

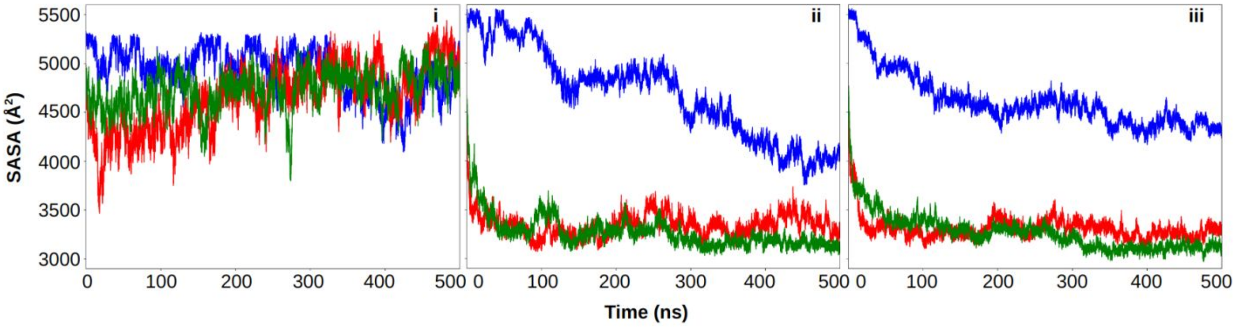

Figure S21: Time evolution of SASA of glucan chains amylose (blue), dextran (red), and pullulan (green) using (i) CHARMM, (ii) GLYCAM, and (iii) OPLS FFs.

Amylose exhibited higher SASA values than dextran and pullulan, indicating greater solvent exposure of the α-(1➞4)-linked glucan backbone. In GLYCAM and OPLS simulations, amylose showed average SASA values of ~3990 Å^2 and ~4353 Å^2, respectively, consistent with its more extended and conformationally constrained structure inferred from R_g and R_h analyses. In CHARMM, amylose displays both higher SASA (~4816 Å^2) and reduced temporal fluctuations, suggesting a more stable, stretched conformation with sustained exposure to water. In contrast, dextran and pullulan exhibited lower SASA values and a gradual reduction in solvent accessibility over time in GLYCAM and OPLS simulations. This behavior reflects how α-(1➞6) linkages can promote chain collapse and reduce exposure of the glucan surface to solvent. Overall, the SASA

analysis demonstrated that the structure of the proximal solvent depends strongly on glycosidic linkage topology.

Table S10: Ensemble-average values of the solvent accessible surface area (SASA) at different timesteps (ns) for different glucan-water systems. The RSE is expressed as a percentage below the mean values.

Ensemble-averaged SASA (Å^2) at different time steps (ns)					
**Force Field**	**Glucan**	**301-350**	**351-400**	**401-450**	**451-500**
CHARMM	Amylose	4976±225	4668±261	4572±235	4816±184
		2.62	3.23	2.97	2.20
	Dextran	4891±190	4906±170	4667±239	5111±64
		2.24	2.00	2.96	0.72
	Pullulan	4837±228	4775±93	4790±125	4909±170
		2.73	1.13	1.50	2.00
GLYCAM	Amylose	4503±459	4276±497	4092±416	3990±337
		5.88	6.71	5.87	4.88
	Dextran	3313±53	3292±132	3419±170	3352±183
		0.92	2.32	2.87	3.15
	Pullulan	3142±172	3176±213	3168±209	3145±197
		3.17	3.87	3.82	3.62
OPLS	Amylose	4531±705	4353±792	4398±742	4353±820
		8.98	10.51	9.74	10.88
	Dextran	3341±52	3240±34	3263±35	3261±24
		0.90	0.61	0.62	0.42
	Pullulan	3134±86	3093±118	3111±115	3121±35
		1.58	2.20	2.14	0.65

Table S11: Block averages of the solvent accessible surface area (SASA) at different timesteps for different glucan-water systems at each run.

			SASA (Å^2) at different time ranges (ns)			
**Force Field**	**Glucan**	**Run**	**301-350**	**351-400**	**401-450**	**451-500**
CHARMM	Amylose	1	5037±244	5036±316	4818±311	5012±247
		2	5217±101	4519±460	4255±188	4570±331
		3	4675±481	4450±501	4642±536	4865±376
	Dxtran	1	4742±274	4666±355	4515±315	5021±219
		2	5159±232	5030±272	4481±258	5147±219
		3	4771±362	5023±289	5004±295	5165±209
	Pullulan	1	4659±254	4903±273	4964±225	5033±198
		2	4694±339	4683±319	4677±267	4668±209
		3	5160±162	4739±302	4729±435	5025±202
GLYCAM	Amylose	1	4975±228	4974±154	4673±184	4396±117
		2	3882±64	3865±96	3881±225	4006±124
		3	4650±281	3987±232	3722±108	3570±114
	Dextran	1	3301±156	3246±82	3390±180	3354±149
		2	3256±97	3159±109	3227±127	3127±73
		3	3383±134	3473±172	3641±152	3576±229
	Pullulan	1	2909±95	2893±102	2904±78	2902±70
		2	3322±86	3406±98	3417±69	3385±94
		3	3195±70	3229±70	3183±92	3148±75
OPLS	Amylose	1	4414±234	4120±151	4048±147	3901±90
		2	5447±126	5419±144	5431±118	5504±59
		3	3732±133	3520±92	3717±147	3653±154
	Dextran	1	3414±112	3215±148	3229±100	3294±114
		2	3298±82	3215±69	3249±97	3239±89
		3	3310±78	3288±106	3312±81	3250±80
	Pullulan	1	3063±132	2953±68	2981±85	3169±96
		2	3255±95	3241±95	3261±107	3109±94
		3	3084±53	3083±69	3090±67	3084±52

S3.2 Evolution of Solvent Near Glucan Chains

To quantify the extent and temporal stability of glucan hydration, we calculated the number of water molecules residing in the immediate vicinity of glucans (first hydration shell (< ~3.8 Å) - a region covering the first trough of the RDF between oxygens on -OH of glucan and water-oxygen atoms). While SASA and RDF analyses characterize solvent exposure and spatial organization, respectively, the number of water molecules in the first hydration shell provides a direct, time-resolved measure of hydration strength at the glucan-water interface. This analysis, therefore, offers a dynamic perspective on the structure of the proximal solvent. **Table S12** shows the evolution of water molecules per monomer (which was calculated by dividing the total number of molecules by the number of monomers (*i.e.*, 30-mer)) at different simulation times, along with the RSE. The average number of water molecules for the entire glucan chain and their evolution during the entire 500 ns simulations are shown in **Table S13** and **Figure S22**, respectively. **Table S14** shows the results for each run.

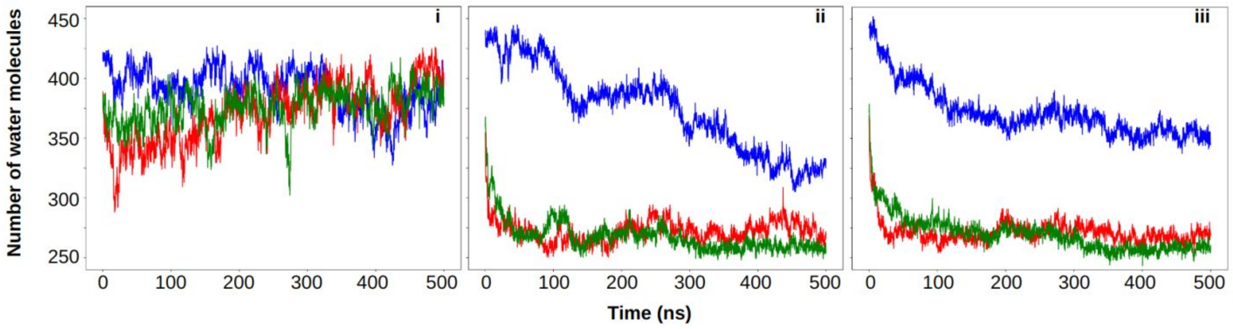

Figure S22: Evolution of the <number of water molecules> within the first hydration shell of different 30-mer glucans, amylose (blue), dextran (red), and pullulan (green) using (i) CHARMM, (ii) GLYCAM, and (iii) OPLS FF.

Table S12: Ensemble Average values of the number of water molecules per monomer in the first hydration shell about glucan chains at different timesteps. The RSE is expressed as a percentage below the mean values.

Force Field	Glucan	301-350	351-400	401-450	451-500
CHARMM	Amylose	13±1	12±1	12±1	13±0
		2.61	3.16	2.99	2.14
	Dextran	13±1	13±0	12±1	14±0
		2.24	1.88	2.83	0.57
	Pullulan	13±1	13±0	13±0	13±0
		2.73	1.17	1.55	1.96
GLYCAM	Amylose	12±1	11±1	11±1	11±1
		5.70	6.56	5.66	4.70
	Dextran	9±0	9±0	9±0	9±0
		0.70	1.90	2.47	2.85
	Pullulan	9±0	9±1	9±1	9±0
		2.99	3.69	3.37	3.19
OPLS	Amylose	12±2	12±2	12±2	12±2
		8.51	9.90	9.24	10.36
	Dextran	9±0	9±0	9±0	9±0
		0.85	0.54	0.51	0.79
	Pullulan	9±0	9±0	9±0	9±0
		1.68	2.49	2.47	0.53

In GLYCAM and OPLS simulations, amylose consistently exhibited a larger number of water molecules in its first hydration shell (~11 to 12 molecules per monomer) compared to dextran and pullulan (~9 molecules per monomer), reflecting its more extended and solvent-exposed conformation. In contrast, CHARMM predicts a similar level of hydration (~13 molecules per monomer) for all three glucans, indicating reduced differentiation in hydration behavior across linkage types. These trends are consistent with SASA results, which show enhanced solvent exposure and more structured hydration shells for amylose relative to dextran and pullulan in

GLYCAM and OPLS. Temporal fluctuations in the number of hydration-shell water molecules further mirror the conformational dynamics of the chains, with CHARMM exhibiting greater variability consistent with enhanced chain flexibility.

Overall, the evolution of water molecules in the first hydration shell provides a quantitative and dynamic description of glucan hydration, with amylose maintaining a more highly hydrated interface, and dextran and pullulan exhibiting reduced hydration due to increased intramolecular collapse. Together with SASA and RDF analyses, these results establish a coherent molecular picture of how glycosidic linkage chemistry governs the structure and dynamics of interfacial water.

Table S13: <Number of water molecules> in the first hydration shell about glucan chains at different timesteps.

Force Field	Glucan	301-350	351-400	401-450	451-500
CHARMM	Amylose	395±18	371±20	363±19	382±14
		2.61	3.16	2.99	2.14
	Dextran	389±15	390±13	371±18	406±4
		2.24	1.88	2.83	0.57
	Pullulan	384±18	379±8	381±10	390±13
		2.73	1.17	1.55	1.96
GLYCAM	Amylose	360±36	342±39	329±32	321±26
		5.70	6.56	5.66	4.70
	Dextran	270±3	269±9	279±12	273±13
		0.70	1.90	2.47	2.85
	Pullulan	258±13	259±17	259±15	259±14
		2.99	3.69	3.37	3.19
OPLS	Amylose	366±54	353±61	356±57	352±63
		8.51	9.90	9.24	10.36
	Dextran	274±4	267±2	268±2	268±4
		0.85	0.54	0.51	0.79
	Pullulan	260±8	256±11	257±11	258±2
		1.68	2.49	2.47	0.53

Table S14: Block averages of the number of water molecules in the first hydration shell about glucans at different timesteps at each run.

Force Field	Glucan	Run	301-350	351-400	401-450	451-500
CHARMM	Amylose	1	399±21	400±26	383±24	397±21
		2	414±12	359±37	338±17	363±27
		3	371±38	355±39	369±43	386±31
	Dxtran	1	378±22	372±28	360±25	400±19
		2	410±19	399±21	357±20	408±19
		3	378±28	399±23	397±23	409±18
	Pullulan	1	370±21	390±23	395±19	400±17
		2	373±27	372±26	372±21	371±17
		3	410±15	376±24	375±34	398±17
GLYCAM	Amylose	1	397±19	397±14	374±16	353±11
		2	312±8	310±9	313±18	321±11
		3	371±22	320±19	300±11	289±11
	Dextran	1	270±13	266±9	277±15	274±13
		2	266±10	260±9	265±11	256±8
		3	274±11	281±14	294±13	289±19
	Pullulan	1	240±9	237±9	240±9	242±8
		2	272±8	277±9	277±8	277±9
		3	262±8	263±8	261±10	258±8
OPLS	Amylose	1	356±18	335±14	328±13	318±9
		2	436±13	435±14	435±12	441±10
		3	305±11	290±9	304±13	298±13
	Dextran	1	280±10	266±13	266±9	272±10
		2	271±8	264±8	266±9	263±9
		3	272±8	270±10	271±8	268±8
	Pullulan	1	254±11	242±8	244±9	260±9
		2	271±9	269±9	271±10	260±8
		3	256±7	257±8	257±7	255±7

Section S4. Hydrogen Bond Analysis

Since hydrogen bonds (H-bonds) in MD simulations lack a distinct, fundamental identity, they are calculated post-processing the simulated trajectories using a set of geometric criteria.[27,28] In this work, the number of H-bonds was calculated using the commonly used criterion: a donor-acceptor distance cut-off of 3.5 Å and an angle cut-off of 30° for the hydrogen-donor-acceptor angle.[27,28] We analyzed intermolecular (water-glucan) and intramolecular (glucan-glucan) H-bond characteristics using H-bond autocorrelation. Specifically, using the average overall autocorrelation function (ACF) (**Eq. S6**) implemented in gmx hbond, we have analyzed the H-bond dynamics.[29] The ACF represents the decay of the H-bond correlation over time by analyzing how the relative positions of the oxygen atoms involved in the H-bonds change at different time intervals, which consequently indicates how stable these H-bonds are over time.[30]

$$C(\tau) = \langle s_i(t)\, s_i(t+\tau) \rangle$$ ……………**Eq. S6**

With $s_i(t)=\{0, 1\}$ for H-bond i at time t and $\langle\ \rangle$ denoting the average over all considered H-bonds. Afterward, multi-exponential fits as shown in **Eq. S7** was used to get a good estimate for the H-bond lifetime (τ_{HB}).

$$C(t) = \sum_{i-1}^{N} A_i \exp(-t/\tau_i)$$ ……………**Eq. S7**

Where τ_i is the time constant, A_i is the amplitude of the ith individual decay process, and *N* is the number of exponentials. We systematically identified the optimal N-value that yields $R^2 >$ 0.99 without overfitting. Note that we calculated the average H-bond ACF from three independent trajectories and obtained the amplitude and time constant from a multiexponentially fitted mean. The corresponding values of amplitude and time constant obtained by nonlinear fitting of the averaged ACF for all glucans and FFs are given in **Tables S15, S16,** and **S17**, which are

interpreted as A_i % of H-bonds break in τ_i ps.[31] **Tables S18-S20** show the data for individual runs, and **Figures S23-S25** show the ACF vs Time plots with standard deviations.

Table S15: Multiexponential nonlinear fits to time correlation functions for obtaining average lifetime (ps) of H-bonds formed between water and the glycosidic-linked oxygens of glucans.

Force field	-O- group	A1%	T1	A2%	T2	A3%	T3	A4%	T4
CHARMM	Amylose O4	80.91	0.31	12.98	3.57	6.11	17.63	-	-
	Dextran O6	78.01	0.34	14.16	7	7.83	30.38	-	-
	Pullulan O4	78.51	0.31	13.97	3.48	7.51	21.71	-	-
	Pullulan O6	79.16	0.28	11.53	5.16	9.31	23.6	-	-
GLYCAM	Amylose O4	84.7	0.36	12.46	8.53	2.84	68.4	-	-
	Dextran O6	80.31	0.4	16.43	10.42	3.26	91.02	-	-
	Pullulan O4	80.1	0.32	12.05	4.87	6.9	36.11	-	-
	Pullulan O6	82.51	0.36	13.26	11.03	4.22	169.41	-	-
OPLS	Amylose O4	73.83	0.34	15.67	4.57	8.44	40.71	1.67	474.97
	Dextran O6	65.62	0.4	22.57	7.19	2.78	477.86	9.02	65.76
	Pullulan O4	68.58	0.32	17.88	4.66	9.57	35.35	3.97	579.91
	Pullulan O6	70.08	0.31	14.65	3.76	12.69	26.39	2.57	290.82

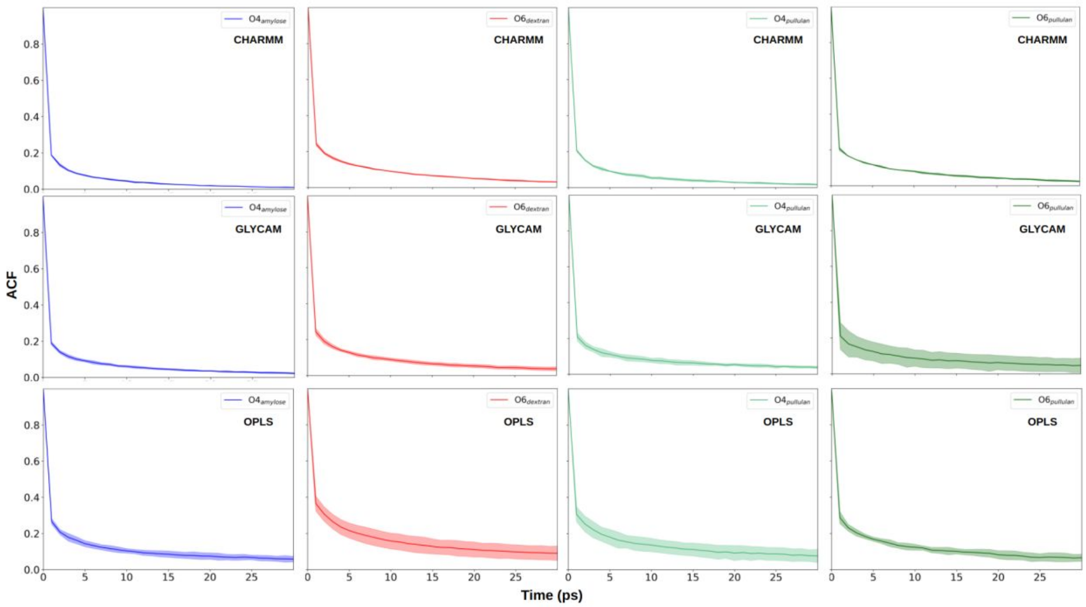

Figure S23: Autocorrelation functions for H-bonds between water and glycosidic oxygens O_4 in α-(1➞4) linked amylose (blue), O_6 in α-(1➞6) linked dextran (red), O_4 in α-(1➞4) linked pullulan (light green), O_6 in α-(1➞6) linked pullulan (dark green) using 3 FFs. The shaded region indicates standard deviation.

Table S16: Multiexponential nonlinear fits to time correlation functions for obtaining average lifetime (ps) of intermolecular H-bonds formed between water and -OH groups of glucans.

Force field	Glucan	A1%	T1	A2%	T2	A3%	T3	A4%	T4
CHARMM	Amylose	48.01	6.56	41.08	0.56	10.88	33.72	-	-
	Dextran	47.25	8.45	39.58	0.68	13.07	45.93	-	-
	Pullulan	47.41	7.37	41.25	0.61	11.27	41.15	-	-
GLYCAM	Amylose	50.46	12.48	41.31	0.97	7.71	104.74	-	-
	Dextran	43.11	8.65	32.37	0.64	20.70	44.96	3.75	370.59
	Pullulan	40.91	8.31	32.11	0.60	21.70	43.37	5.24	405.57

OPLS	Amylose	40.87	12.32	32.13	48.55	5.25	320.36	21.69	0.65
	Dextran	7.47	452.91	30.74	71.16	39.30	14.15	22.37	0.76
	Pullulan	22.74	0.72	9.11	450.02	29.81	65.45	38.23	14.14

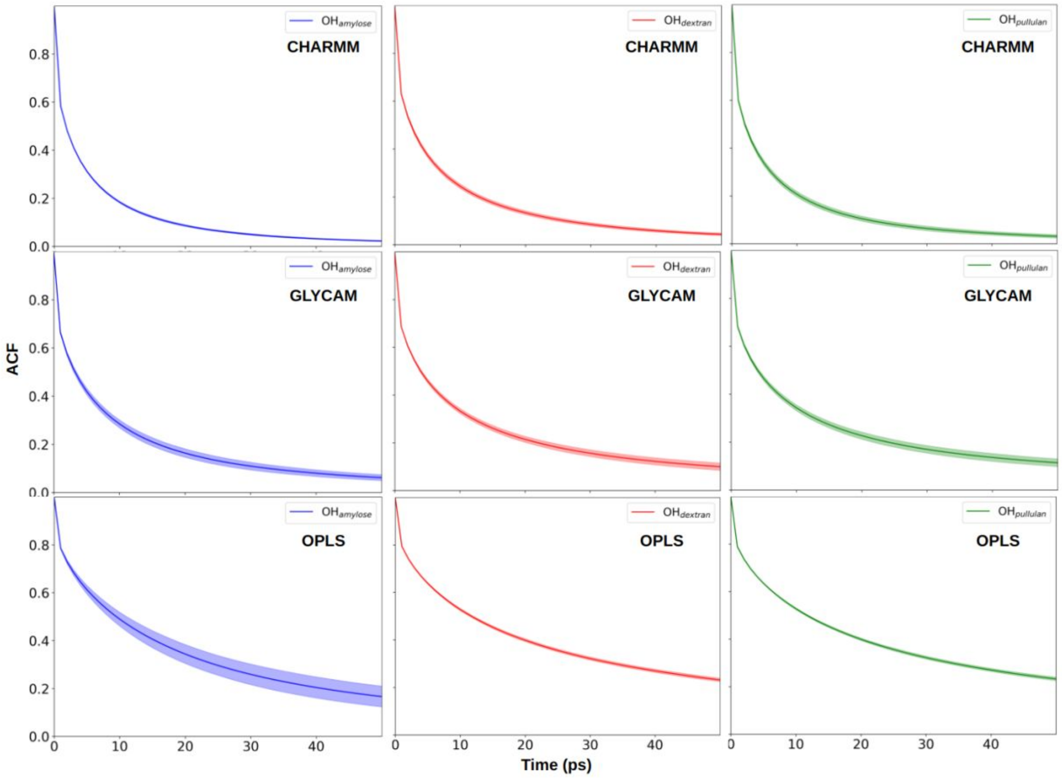

Figure S24: Autocorrelation functions for H-bonds between water and -OH groups of the glucans amylose (blue), dextran (red), and pullulan (green) 3 FFs. The shaded region indicates standard deviations.

Table S17: Multiexponential nonlinear fits to time correlation functions for obtaining average lifetime (ps) of intramolecular H-bonds.

Force field	Glucan	A1%	T1	A2%	T2	A3%	T3	A4%	T4	A5%	T5
CHARMM	Amylose	13.82	2284.23	9.48	19824.11	24.28	30.69	37.09	0.94	14.65	320.28
	Dextran	13.76	2514.50	8.90	21326.28	24.37	31.91	37.32	0.97	14.89	344.30
	Pullulan	14.62	1078.55	50.29	1.52	9.85	9007.65	23.93	66.70	-	-
GLYCAM	Amylose	18.00	2032.44	29.00	81.78	21.77	36068.49	18.70	20.12	12.25	235.54
	Dextran	13.92	1731.76	15.00	98.63	27.90	1.63	42.95	26905.84	-	-
	Pullulan	38.86	1.96	16.72	144.86	26.02	28868.38	17.53	2040.81	-	
OPLS	Amylose	30.56	0.97	23.37	25.71	15.53	2264.92	15.57	302.58	14.36	-25434.44
	Dextran	14.66	821.19	15.86	62.24	37.93	24910.28	30.89	0.91	-	-
	Pullulan	15.00	26.63	22.40	16676.83	16.45	1855.04	13.05	257.28	33.04	0.53

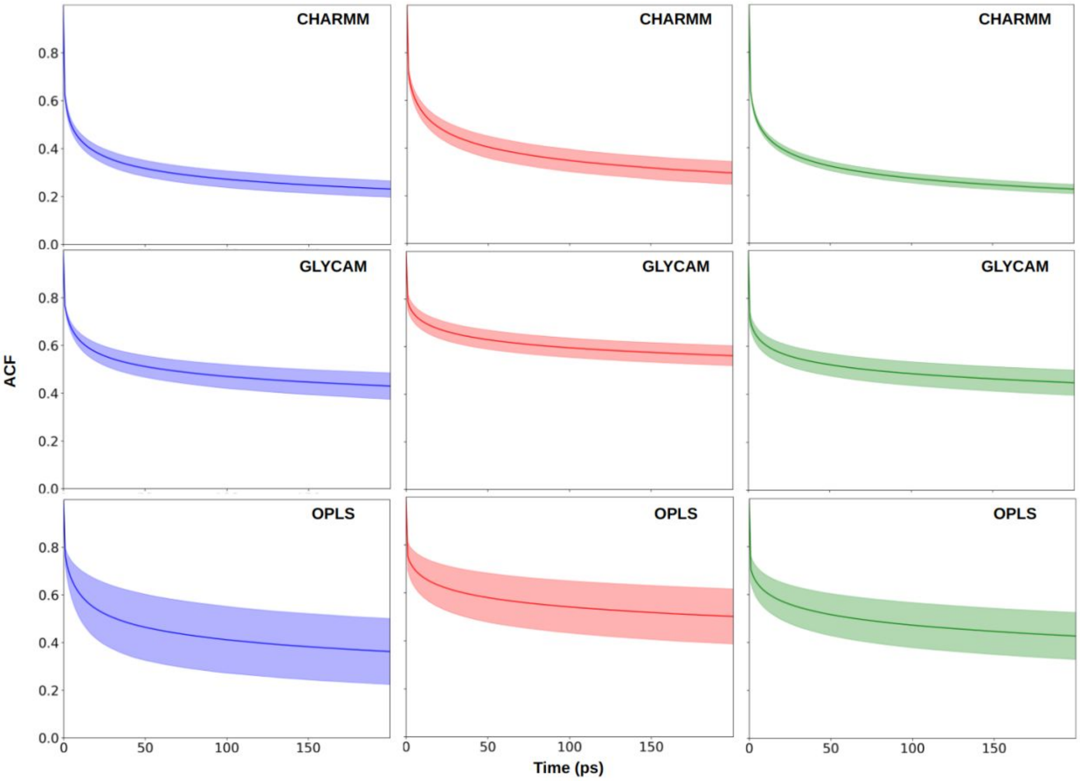

Figure 25: Autocorrelation functions for H-bonds formed within glucan chains amylose (blue), dextran (red), and pullulan (green), using 3 FFs. The shaded region indicates standard deviations.

Table S18: Multiexponential nonlinear fits to time correlation functions for obtaining lifetime (ps) of H-bonds formed between water and the glycosidic-linked oxygens of glucans for each run.

Force field	-O- group	Run	A1%	T1	A2%	T2	A3%	T3	A4%	T4
CHARMM	Amylose O4	1	78.35	0.26	15.49	3.10	6.15	18.56	-	-
		2	82.34	0.33	12.34	3.91	5.32	18.40	-	-
		3	81.22	0.32	11.38	3.47	7.40	15.49	-	-
	Dextran O6	1	75.24	0.32	14.11	4.80	10.65	23.69	-	-
		2	77.63	0.34	12.14	6.19	10.23	24.37	-	-
		3	80.07	0.36	15.19	9.42	4.74	49.58	-	-
	Pullulan O4	1	76.64	0.26	16.05	2.90	7.31	19.77	-	-
		2	75.42	0.27	14.39	2.41	10.18	17.31	-	-
		3	80.25	0.34	13.42	4.45	6.33	27.91	-	-
	Pullulan O6	1	77.92	0.19	11.30	18.72	10.78	3.93	-	-
		2	78.74	0.29	8.96	25.67	12.30	4.85	-	-
		3	80.66	0.33	7.37	28.41	11.97	7.36	-	-
GLYCAM	Amylose O4	1	84.93	0.37	12.03	8.83	3.03	80.46	-	-
		2	86.30	0.37	11.85	9.62	1.84	84.23	-	-
		3	82.79	0.34	13.17	7.26	4.04	42.77	-	-
	Dextran O6	1	80.45	0.39	15.09	11.32	4.44	99.09	-	-
		2	83.79	0.43	15.27	15.89	0.90	1356.50	-	-
		3	73.94	0.34	17.36	4.70	8.70	29.36	-	-
	Pullulan O4	1	82.71	0.39	13.31	13.50	3.90	84.96	-	-
		2	82.01	0.38	13.97	8.62	4.02	118.33	-	-
		3	87.52	0.39	10.92	15.65	1.55	491.85	-	-
	Pullulan O6	1	85.19	0.28	8.86	6.13	5.95	30.87	-	-
		2	85.61	0.34	12.16	10.41	2.23	127.08	-	-
		3	73.83	0.39	16.57	9.20	9.59	187.84	-	-
OPLS	Amylose O4	1	5.18	102.94	16.26	10.10	78.54	0.44	-	-
		2	76.11	0.34	15.01	4.71	8.87	45.57	-	-
		3	14.11	25.75	3.91	743.89	81.88	0.51	-	-

	Dextran O6	1	78.10	0.55	19.51	18.37	2.20	482.80	-	-
		2	12.74	24.71	26.93	3.56	4.41	280.57	55.92	0.26
		3	20.31	10.26	64.95	0.44	2.51	645.98	12.21	108.06
	Pullulan O4	1	4.84	628.11	73.00	0.31	8.10	38.51	14.05	4.46
		2	5.83	549.81	19.43	3.84	13.58	33.32	61.15	0.24
		3	21.05	6.30	1.18	560.81	5.74	43.08	72.03	0.38
	Pullulan O6	1	16.16	38.06	15.62	3.99	68.22	0.32	-	-
		2	13.75	2.63	70.17	0.12	10.29	2.11	5.79	334.60
		3	3.55	80.78	75.18	0.44	21.25	10.26	-	-

Table S19: Multiexponential nonlinear fits to time correlation functions for obtaining lifetime (ps) of intermolecular H-bonds formed between water and -OH groups of glucans for each run.

Force field	-OH group	Run	A1%	T1	A2%	T2	A3%	T3	A4%	T4
CHARMM	Amylose	1	47.75	6.82	41.75	0.58	10.46	36.90	-	-
		2	47.51	6.25	40.61	0.55	11.85	31.04	-	-
		3	48.68	6.58	40.78	0.56	10.51	33.18	-	-
	Dextran	1	46.82	7.78	39.04	0.64	14.06	39.26	-	-
		2	46.25	8.27	38.69	0.66	14.98	44.06	-	-
		3	48.31	9.38	41.14	0.75	10.37	57.42	-	-
	Pullulan	1	46.73	6.43	39.80	0.56	13.43	30.90	-	-
		2	47.84	8.76	43.54	0.71	8.48	61.98	-	-
		3	46.30	6.88	40.11	0.58	13.54	35.61	-	-
GLYCAM	Amylose	1	51.99	11.25	40.40	0.90	7.22	90.70	-	-
		2	50.93	12.39	40.69	0.93	7.92	98.13	-	-
		3	48.44	14.27	43.01	1.09	7.74	131.83	-	-
	Dextran	1	43.03	8.76	32.32	0.64	20.36	48.15	4.22	459.26
		2	4.37	446.49	44.12	9.54	33.40	0.68	18.01	52.31
		3	47.74	12.31	38.18	0.92	13.67	78.32	-	-
	Pullulan	1	7.68	450.66	24.61	36.64	37.79	7.20	29.90	0.52
		2	41.44	8.83	33.15	0.64	21.10	48.05	4.24	434.70
		3	19.71	45.44	4.10	290.77	43.06	8.78	33.07	0.62
OPLS	Amylose	1	14.91	150.47	30.21	1.65	54.15	24.91	-	-
		2	24.39	0.89	54.78	15.58	20.56	62.38	-	-
		3	7.90	403.35	41.16	13.58	28.64	55.86	22.21	0.69
	Dextran	1	6.24	519.39	30.12	73.58	22.77	0.77	40.73	14.75
		2	30.16	69.31	22.34	0.78	38.66	14.30	8.68	364.61
		3	32.65	65.81	8.42	461.97	21.46	0.68	37.39	12.82
	Pullulan	1	36.74	13.28	30.72	61.24	10.48	364.85	21.98	0.68
		2	38.00	14.11	22.99	0.70	9.37	518.99	29.54	67.37
		3	8.63	439.24	37.60	13.72	22.52	0.72	31.14	60.47

Table S20: Multiexponential nonlinear fits to time correlation functions for obtaining average lifetime (ps) of intramolecular H-bonds for each run.

Force field	Glucan	Run	A1%	T1	A2%	T2	A3%	T3	A4%	T4	A5%	T5
CHARMM	Amylose	1	12.19	543.67	11.30	4076.59	51.92	1.00	23.53	35.88	-	-
		2	6.74	34867.43	11.33	342.82	21.40	29.11	47.65	0.82	12.39	2257.55
		3	12.07	1570.73	2.26	50160.73	21.06	91.19	53.31	1.81	10.39	12149.44
	Dextran	1	24.11	46.23	17.55	660.78	19.18	18169.50	38.30	1.33	-	-
		2	6.37	26283.10	35.85	0.96	14.71	349.51	23.22	33.35	19.13	2811.03
		3	39.61	0.88	16.40	2971.21	16.14	290.84	26.16	26.50	1.12	63112.21
	Pullulan	1	52.60	1.41	8.61	8307.72	14.33	935.41	23.68	59.57	-	-
		2	15.17	986.36	22.09	66.82	11.93	9010.82	48.76	1.46	-	-
		3	12.54	664.35	10.95	4217.75	1.38	155256.40	49.76	1.14	23.81	45.33
GLYCAM	Amylose	1	17.65	4179.78	15.96	476.18	11.34	24434.71	25.20	22.25	29.84	0.01
		2	30.71	1.20	34.01	38550.22	18.37	974.63	15.77	40.27	-	-
		3	17.97	44698.23	14.63	272.26	18.42	2383.65	29.50	0.86	19.10	25.28
	Dextran	1	15.64	2043.25	26.65	0.47	35.45	32062.41	11.39	16.53	10.85	137.25
		2	13.83	62.02	27.14	1.08	13.07	1141.23	45.07	23011.41	-	-
		3	16.41	1857.38	23.88	6.47	11.52	161.01	46.80	26846.44	-	-
	Pullulan	1	35.25	31522.43	20.68	1001.54	35.75	0.01	8.54	0.01	-	-
		2	19.47	3336.66	15.34	31229.21	34.55	0.70	14.33	339.17	16.09	29.88
		3	26.09	25280.31	9.95	3016.54	13.03	340.91	14.54	24.40	36.30	0.58
OPLS	Amylose	1	9.97	1572.25	22.79	31752.41	24.08	108.40	32.75	2.08	9.61	1573.51
		2	17.89	589.49	8.47	3176.54	43.38	2.53	28.93	37.51	-	-

		3	18.50	6225.07	19.53	55.74	30.38	1.37	16.81	675.66	12.96	27629.45
	Dextran	1	24.01	0.66	49.17	22586.71	13.38	423.39	13.31	36.42	-	-
		2	13.58	865.17	8.79	12.15	44.88	27076.92	22.95	0.36	9.80	93.81
		3	15.80	14.19	17.41	1346.52	18.89	28066.44	14.93	127.30	32.97	0.42
	Pullulan	1	27.18	0.55	27.75	23106.14	19.87	3946.11	13.28	309.42	11.84	28.77
		2	23.56	1461.52	19.74	105.98	18.41	17162.32	37.52	1.41	-	-
		3	20.92	85.81	44.25	1.02	16.90	12422.81	17.55	1104.35	-	-

Section S5. Nonbonded Interaction Energies

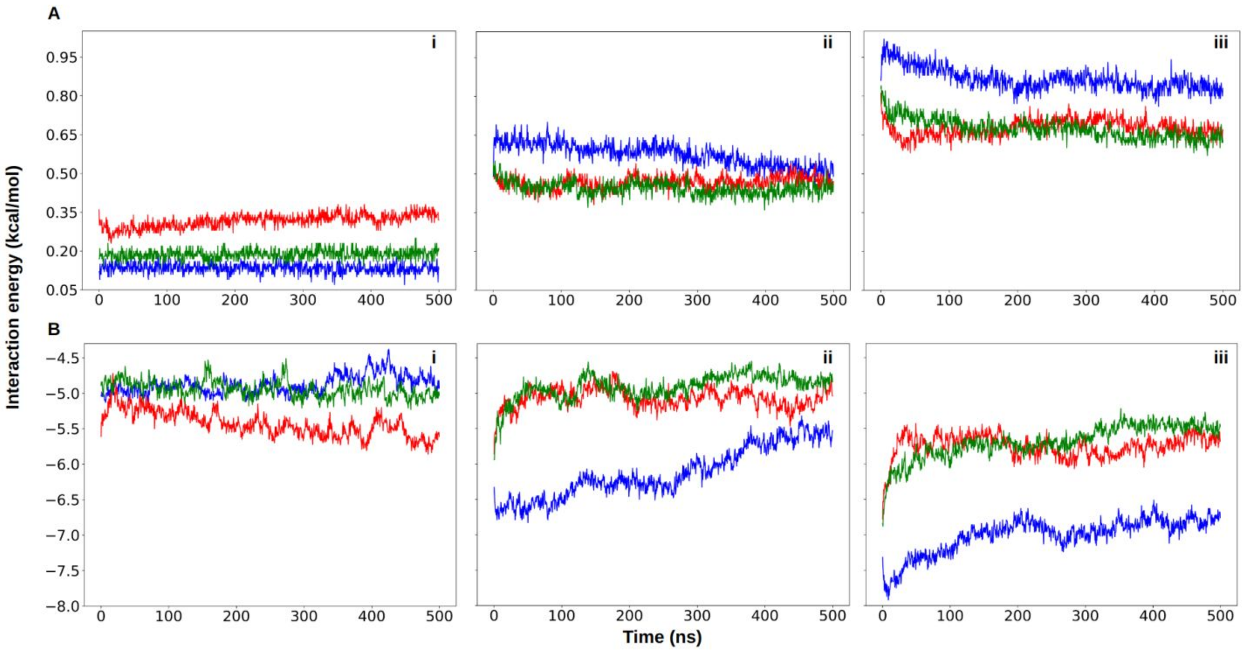

Figure S26: Normalized intermolecular nonbonded interaction energy for A) nonpolar-water and B) polar-water interactions for amylose (blue), dextran (red), and pullulan (green), using (i) CHARMM, (ii) GLYCAM, (iii) OPLS FFs.

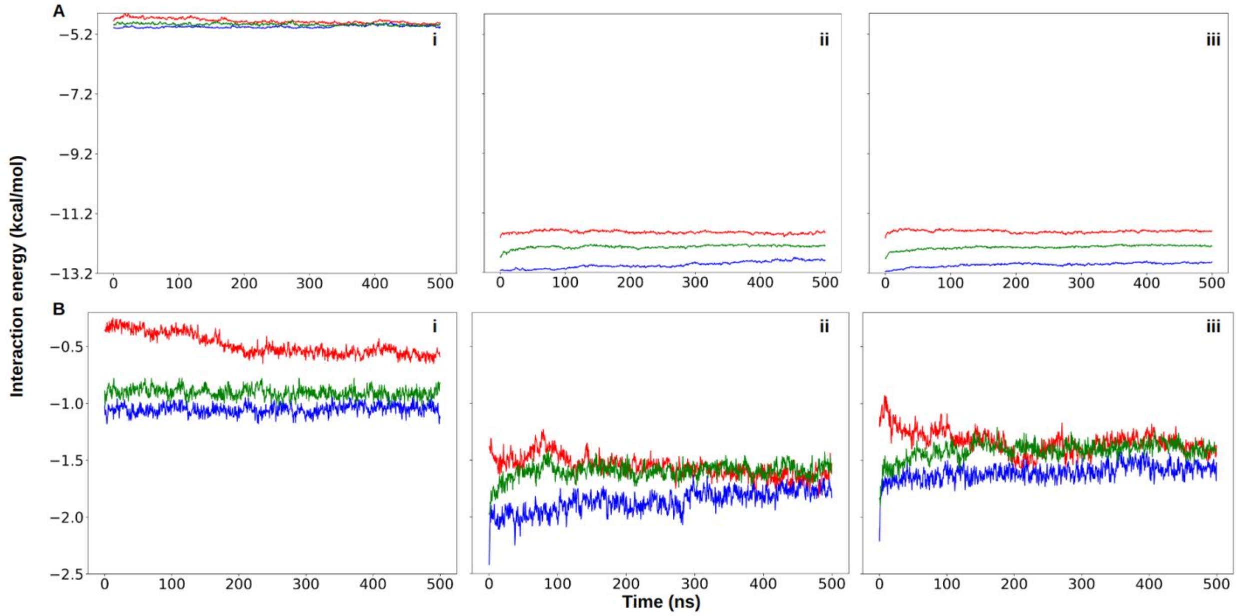

Figure S27: Normalized intramolecular non-bonded interaction energy for A) nonpolar-nonpolar and B) polar-polar interactions for amylose (blue), dextran (red), and pullulan (green), using (i) CHARMM, (ii) GLYCA, and (iii) OPLS FFs.

Table S21: Ensemble-averaged values of normalized nonbonded interaction energies between glucan atoms (glycosidic-linked oxygens) and water for each run. The data is normalized by dividing the total energy by the number of respective atoms.

Force field	Glucans	# of glucan atoms used for normalization	Run	Energy (kcal/mol) at different timesteps (ns)			
				301-350	351-400	401-450	451-500
CHARMM	Amylose O4-Water	29	1	-2.21±0.41	-2.24±0.38	-2.14±0.41	-2.17±0.38
			2	-2.21±0.41	-2.10±0.41	-2.00±0.34	-2.07±0.41
			3	-2.10±0.41	-2.03±0.41	-2.17±0.41	-2.21±0.41
	Dextran O6-Water	29	1	-4.31±0.52	-4.34±0.55	-4.24±0.52	-4.59±0.48
			2	-4.62±0.48	-4.59±0.48	-4.24±0.52	-4.55±0.48
			3	-4.34±0.48	-4.52±0.48	-4.52±0.52	-4.59±0.48
	Pullulan O4-Water	20	1	-2.35±0.50	-2.30±0.50	-2.30±0.45	-2.30±0.45
			2	-2.20±0.45	-2.10±0.45	-2.15±0.45	-2.25±0.50
			3	-2.35±0.50	-2.30±0.45	-2.20±0.45	-2.25±0.45
	Pullulan O6-Water	9	1	-3.89±0.78	-4.11±0.78	-4.11±0.78	-4.11±0.78
			2	-3.89±0.78	-3.78±0.78	-3.89±0.78	-3.78±0.78
			3	-4.22±0.78	-4.00±0.78	-4.00±0.78	-4.11±0.78
GLYCAM	Amylose O4-Water	29	1	-4.52±0.59	-4.59±0.55	-4.31±0.55	-4.28±0.48
			2	-3.83±0.45	-3.79±0.48	-3.97±0.48	-3.97±0.48
			3	-4.31±0.62	-3.76±0.52	-3.62±0.48	-3.59±0.48
	Dextran O6-Water	29	1	-4.62±0.55	-4.52±0.48	-4.52±0.55	-4.24±0.52
			2	-4.17±0.52	-4.17±0.52	-4.48±0.55	-4.21±0.48
			3	-4.52±0.52	-4.31±0.52	-4.62±0.59	-4.97±0.59
	Pullulan O4-Water	20	1	-3.00±0.55	-2.65±0.55	-2.85±0.60	-2.95±0.60
			2	-3.55±0.60	-3.50±0.60	-3.35±0.60	-3.45±0.60
			3	-3.25±0.55	-3.20±0.55	-3.25±0.55	-3.10±0.55
	Pullulan O6-Water	9	1	-4.11±0.89	-4.00±0.89	-4.00±0.89	-4.11±0.89
			2	-4.22±0.89	-4.22±0.89	-4.11±0.89	-4.33±0.78

			3	-4.56±0.89	-4.56±0.89	-4.78±0.89	-4.78±0.89
OPLS	Amylose O4-Water	29	1	-4.93±0.59	-4.59±0.59	-4.45±0.52	-4.38±0.52
			2	-5.55±0.55	-5.55±0.55	-5.55±0.59	-5.59±0.55
			3	-4.21±0.52	-4.07±0.48	-4.24±0.55	-4.10±0.45
	Dextran O6-Water	29	1	-5.14±0.52	-4.93±0.52	-4.90±0.48	-4.76±0.48
			2	-4.62±0.48	-4.66±0.52	-4.45±0.48	-4.24±0.48
			3	-5.03±0.52	-4.97±0.48	-5.00±0.52	-4.72±0.48
	Pullulan O4-Water	20	1	-3.30±0.55	-3.15±0.55	-3.10±0.50	-3.25±0.55
			2	-3.70±0.55	-3.65±0.55	-3.75±0.50	-3.60±0.55
			3	-3.55±0.50	-3.50±0.55	-3.50±0.55	-3.40±0.50
	Pullulan O6-Water	9	1	-4.78±1.00	-4.56±1.00	-4.67±1.00	-4.44±0.89
			2	-5.22±0.89	-5.00±0.89	-5.11±0.89	-5.11±1.00
			3	-4.78±0.89	-4.44±0.89	-4.56±0.89	-4.67±0.89

Table S22: Ensemble-averaged values of normalized nonbonded interaction energies between glucan nonpolar atoms and water for each run. The data is normalized by dividing the total energy by the number of respective atoms (390).

Force field	Glucans	Run	Energy (kcal/mol) at different timesteps (ns)			
			**301-350**	**351-400**	**401-450**	**451-500**
CHARMM	Amylose	1	0.13±0.06	0.14±0.06	0.13±0.06	0.13±0.06
		2	0.13±0.07	0.13±0.06	0.12±0.06	0.13±0.06
		3	0.13±0.06	0.13±0.06	0.14±0.07	0.14±0.06
	Dextran	1	0.32±0.06	0.32±0.07	0.32±0.06	0.34±0.06
		2	0.35±0.06	0.35±0.06	0.32±0.06	0.34±0.06
		3	0.32±0.06	0.34±0.06	0.34±0.06	0.35±0.06
	Pullulan	1	0.20±0.07	0.20±0.06	0.19±0.06	0.21±0.07
		2	0.19±0.06	0.18±0.06	0.18±0.06	0.18±0.06
		3	0.20±0.07	0.19±0.06	0.19±0.06	0.19±0.07
GLYCAM	Amylose	1	0.60±0.08	0.61±0.09	0.57±0.08	0.55±0.07
		2	0.52±0.08	0.51±0.08	0.53±0.08	0.52±0.08
		3	0.57±0.09	0.50±0.08	0.49±0.08	0.48±0.08
	Dextran	1	0.48±0.07	0.47±0.06	0.48±0.07	0.46±0.07
		2	0.44±0.07	0.45±0.07	0.48±0.07	0.45±0.07
		3	0.48±0.07	0.46±0.07	0.49±0.07	0.52±0.07
	Pullulan	1	0.38±0.07	0.37±0.06	0.39±0.07	0.41±0.07
		2	0.46±0.07	0.48±0.07	0.46±0.08	0.47±0.07
		3	0.44±0.07	0.43±0.07	0.45±0.07	0.44±0.07
OPLS	Amylose	1	0.86±0.09	0.82±0.08	0.80±0.08	0.77±0.08
		2	0.97±0.08	0.97±0.08	0.97±0.09	0.98±0.08
		3	0.74±0.08	0.72±0.07	0.75±0.08	0.73±0.07
	Dextran	1	0.73±0.07	0.68±0.07	0.68±0.07	0.68±0.07
		2	0.67±0.07	0.66±0.07	0.65±0.07	0.62±0.07
		3	0.73±0.07	0.71±0.07	0.70±0.07	0.69±0.07
	Pullulan	1	0.63±0.07	0.57±0.07	0.58±0.07	0.62±0.07

		2	0.71±0.07	0.70±0.07	0.71±0.07	0.67±0.07
		3	0.64±0.07	0.63±0.07	0.63±0.07	0.61±0.07

Table S23: Ensemble-averaged values of normalized nonbonded interaction energies between glucan polar atoms and water for each run. The data is normalized by dividing the total energy by the number of respective atoms (243).

Force field	Glucans	Run	time			
			301-350	351-400	401-450	451-500
CHARMM	Amylose	1	-4.93±0.23	-4.93±0.24	-4.84±0.25	-4.91±0.21
		2	-5.01±0.18	-4.66±0.33	-4.46±0.20	-4.66±0.25
		3	-4.74±0.35	-4.60±0.33	-4.76±0.33	-4.87±0.26
	Dextran	1	-5.47±0.22	-5.45±0.26	-5.31±0.25	-5.63±0.19
		2	-5.69±0.21	-5.65±0.22	-5.35±0.23	-5.69±0.21
		3	-5.51±0.25	-5.64±0.23	-5.62±0.23	-5.71±0.20
	Pullulan	1	-4.96±0.20	-5.06±0.23	-5.07±0.21	-5.12±0.21
		2	-4.91±0.24	-4.84±0.27	-4.89±0.24	-4.91±0.19
		3	-5.19±0.21	-4.97±0.23	-4.98±0.26	-5.10±0.20
GLYCAM	Amylose	1	-6.38±0.27	-6.37±0.25	-6.11±0.28	-5.89±0.22
		2	-5.51±0.21	-5.43±0.22	-5.58±0.28	-5.57±0.24
		3	-6.16±0.35	-5.50±0.33	-5.28±0.23	-5.18±0.24
	Dextran	1	-5.08±0.26	-4.99±0.20	-5.16±0.29	-5.12±0.26
		2	-5.00±0.25	-4.96±0.23	-5.09±0.25	-4.91±0.21
		3	-5.00±0.23	-5.10±0.23	-5.28±0.26	-5.21±0.32
	Pullulan	1	-4.55±0.19	-4.39±0.20	-4.60±0.25	-4.74±0.20
		2	-5.01±0.20	-5.07±0.21	-5.00±0.21	-5.09±0.22
		3	-4.83±0.21	-4.69±0.19	-4.76±0.21	-4.70±0.20
OPLS	Amylose	1	-7.00±0.32	-6.68±0.25	-6.56±0.25	-6.45±0.24
		2	-7.71±0.25	-7.74±0.24	-7.73±0.24	-7.81±0.23
		3	-6.12±0.24	-6.06±0.23	-6.21±0.28	-6.07±0.23

	Dextran	1	-6.12±0.23	-5.84±0.26	-5.74±0.22	-5.74±0.21
		2	-5.73±0.23	-5.67±0.22	-5.64±0.24	-5.42±0.22
		3	-5.76±0.21	-5.79±0.20	-5.80±0.20	-5.72±0.21
	Pullulan	1	-5.37±0.26	-4.97±0.24	-5.00±0.23	-5.35±0.21
		2	-5.81±0.21	-5.80±0.21	-5.84±0.22	-5.64±0.21
		3	-5.47±0.20	-5.55±0.20	-5.52±0.19	-5.43±0.20

Table S24: Ensemble-averaged values of normalized nonbonded intramolecular interaction energies between nonpolar-nonpolar atoms of glucans for each run. The data is normalized by dividing the total energy by the number of respective atoms (390).

**Force field**	**Glucans**	Run	**Energy (kcal/mol) at different timesteps (ns)**			
			**301-350**	**351-400**	**401-450**	**451-500**
CHARMM	Amylose	1	-4.97±0.04	-4.96±0.05	-4.94±0.05	-4.96±0.05
		2	-5.00±0.02	-4.87±0.09	-4.83±0.05	-4.87±0.07
		3	-4.90±0.10	-4.85±0.10	-4.89±0.10	-4.93±0.07
	Dextran	1	-4.77±0.06	-4.77±0.06	-4.73±0.06	-4.83±0.04
		2	-4.86±0.04	-4.85±0.05	-4.74±0.05	-4.85±0.04
		3	-4.79±0.06	-4.84±0.05	-4.84±0.05	-4.87±0.04
	Pullulan	1	-4.87±0.05	-4.91±0.04	-4.91±0.05	-4.92±0.04
		2	-4.88±0.05	-4.85±0.07	-4.87±0.05	-4.86±0.05
		3	-4.94±0.04	-4.87±0.06	-4.88±0.07	-4.92±0.04
GLYCAM	Amylose	1	-13.03±0.06	-13.03±0.04	-12.97±0.06	-12.88±0.04
		2	-12.74±0.03	-12.75±0.03	-12.79±0.07	-12.79±0.05
		3	-12.97±0.09	-12.77±0.08	-12.68±0.05	-12.65±0.06
	Dextran	1	-11.89±0.06	-11.90±0.04	-11.94±0.08	-11.89±0.07
		2	-11.81±0.05	-11.83±0.07	-11.87±0.07	-11.76±0.05
		3	-11.85±0.06	-11.85±0.06	-11.91±0.07	-12.00±0.09
	Pullulan	1	-12.22±0.05	-12.17±0.05	-12.23±0.06	-12.27±0.04
		2	-12.38±0.04	-12.42±0.04	-12.38±0.05	-12.41±0.05

		3	-12.31±0.04	-12.27±0.04	-12.30±0.04	-12.28±0.04
OPLS	Amylose	1	-12.91±0.06	-12.83±0.05	-12.79±0.05	-12.76±0.04
		2	-13.13±0.03	-13.12±0.03	-13.12±0.03	-13.13±0.02
		3	-12.69±0.05	-12.63±0.04	-12.71±0.05	-12.68±0.04
	Dextran	1	-11.89±0.06	-11.84±0.06	-11.85±0.05	-11.87±0.04
		2	-11.83±0.05	-11.80±0.05	-11.80±0.05	-11.76±0.05
		3	-11.80±0.05	-11.76±0.05	-11.75±0.05	-11.75±0.05
	Pullulan	1	-12.27±0.05	-12.15±0.05	-12.15±0.05	-12.25±0.04
		2	-12.36±0.04	-12.36±0.04	-12.36±0.03	-12.33±0.04
		3	-12.30±0.03	-12.31±0.04	-12.31±0.04	-12.29±0.03

Table S25: Ensemble-averaged values of normalized nonbonded intramolecular interaction energies between polar-polar atoms of glucans for each run. The data is normalized by dividing the total energy by the number of respective atoms (243).

**Force field**	**Glucans**	Run	**Energy (kcal/mol) at different timesteps (ns)**			
			**301-350**	**351-400**	**401-450**	**451-500**
CHARMM	Amylose	1	-1.07±0.14	-1.07±0.11	-1.07±0.12	-1.08±0.13
		2	-1.09±0.12	-1.02±0.14	-1.03±0.13	-1.05±0.12
		3	-1.04±0.13	-1.00±0.13	-1.00±0.12	-1.01±0.14
	Dextran	1	-0.51±0.11	-0.53±0.11	-0.50±0.11	-0.55±0.10
		2	-0.61±0.09	-0.58±0.11	-0.54±0.11	-0.58±0.10
		3	-0.54±0.10	-0.58±0.10	-0.57±0.11	-0.61±0.10
	Pullulan	1	-0.87±0.12	-0.92±0.13	-0.93±0.12	-0.91±0.12
		2	-0.94±0.12	-0.93±0.13	-0.92±0.13	-0.90±0.12
		3	-0.93±0.12	-0.88±0.12	-0.93±0.12	-0.94±0.12
GLYCAM	Amylose	1	-1.91±0.17	-1.91±0.16	-1.90±0.16	-1.82±0.16
		2	-1.62±0.14	-1.70±0.16	-1.76±0.16	-1.77±0.15
		3	-1.90±0.17	-1.79±0.18	-1.68±0.17	-1.64±0.18
	Dextran	1	-1.71±0.15	-1.79±0.13	-1.78±0.17	-1.63±0.17

		2	-1.47±0.15	-1.54±0.16	-1.58±0.16	-1.38±0.14
		3	-1.58±0.14	-1.53±0.16	-1.58±0.18	-1.91±0.15
	Pullulan	1	-1.54±0.14	-1.49±0.14	-1.52±0.13	-1.53±0.14
		2	-1.66±0.14	-1.71±0.15	-1.64±0.16	-1.69±0.15
		3	-1.52±0.15	-1.56±0.14	-1.55±0.15	-1.56±0.17
OPLS	Amylose	1	-1.54±0.15	-1.50±0.15	-1.53±0.16	-1.52±0.15
		2	-1.74±0.14	-1.71±0.15	-1.71±0.15	-1.70±0.14
		3	-1.51±0.16	-1.34±0.15	-1.49±0.16	-1.49±0.16
	Dextran	1	-1.37±0.18	-1.36±0.16	-1.48±0.16	-1.58±0.13
		2	-1.45±0.14	-1.43±0.14	-1.39±0.14	-1.40±0.14
		3	-1.31±0.14	-1.16±0.15	-1.12±0.14	-1.21±0.14
	Pullulan	1	-1.39±0.15	-1.31±0.14	-1.23±0.14	-1.39±0.15
		2	-1.36±0.14	-1.37±0.14	-1.36±0.14	-1.38±0.13
		3	-1.47±0.13	-1.47±0.15	-1.47±0.14	-1.48±0.14

References

(1) Zhu, G.; Li, H.; Li, Y.; Gu, L. ^{1}H NMR Elucidation of Observed Stable sugar-NaCl-water Complexes in Aqueous Solution. *Chem. Methods* **2023**, *3*.

(2) Boimirzaev, A. S.; Shomurotov, S.; Turaev, A. S. SECONDARY EFFECTS IN AQUEOUS SIZE-EXCLUSION CHROMATOGRAPHY OF POLYSACCHARIDES. *Химия растительного сырья* **2013**, 51–55.

(3) Antoniou, E.; Themistou, E.; Sarkar, B.; Tsianou, M.; Alexandridis, P. Structure and Dynamics of Dextran in Binary Mixtures of a Good and a Bad Solvent. *Colloid Polym. Sci.* **2010**, *288*, 1301–1312.

(4) Dünweg, B.; Kremer, K. Molecular Dynamics Simulation of a Polymer Chain in Solution. *J. Chem. Phys.* **1993**, *99*, 6983–6997.

(5) Nygaard, M.; Kragelund, B. B.; Papaleo, E.; Lindorff-Larsen, K. An Efficient Method for Estimating the Hydrodynamic Radius of Disordered Protein Conformations. *Biophys. J.* **2017**, *113*, 550–557.

(6) Doi, M.; Edwards, S. F.; Edwards, S. F. *The Theory of Polymer Dynamics*; Oxford University Press, 1988.

(7) Fetters, L. J.; Hadjichristidis, N.; Lindner, J. S.; Mays, J. W. Molecular Weight Dependence of Hydrodynamic and Thermodynamic Properties for Well-Defined Linear Polymers in Solution. *J. Phys. Chem. Ref. Data* **1994**, *23*, 619–640.

(8) Ioan, C. E.; Aberle, T.; Burchard, W. Structure Properties of Dextran. 2. Dilute Solution. *Macromolecules* **2000**, *33*, 5730–5739.

(9) Nordmeier, E. Static and Dynamic Light-Scattering Solution Behavior of Pullulan and Dextran in Comparison. *J. Phys. Chem.* **1993**, *97*, 5770–5785.

(10) Nishinari, K.; Kohyama, K.; Williams, P. A.; Phillips, G. O.; Burchard, W.; Ogino, K. Solution Properties of Pullulan. *Macromolecules* **1991**, *24*, 5590–5593.

(11) Roger, P.; Colonna, P. Molecular Weight Distribution of Amylose Fractions Obtained by Aqueous Leaching of Corn Starch. *Int. J. Biol. Macromol.* **1996**, *19*, 51–61.

(12) Roger, P.; Colonna, P. Evidence of the Presence of Large Aggregates Contaminating Amylose Solutions. *Carbohydr. Polym.* **1993**, *21*, 83–89.

(13) Sileshi, G. W. The Relative Standard Error as an Easy Index for Checking the Reliability of Regression Coefficients, 2015.

(14) Sedgwick, P. Standard Deviation or the Standard Error of the Mean. *BMJ* **2015**, *350*, h831.

(15) Ring, S. G.; L'Anson, K.; Morris, V. J. Static and Dynamic Light Scattering Studies of Amylose Solutions. *Macromolecules* **1985**, *18*, 182–188.

(16) Kato, T.; Katsuki, T.; Takahashi, A. Static and Dynamic Solution Properties of Pullulan in a Dilute Solution. *Macromolecules* **1984**, *17*, 1726–1730.

(17) Gerbst, A. G.; Grachev, A. A.; Shashkov, A. S.; Nifantiev, N. E. Computation Techniques in the Conformational Analysis of Carbohydrates. *Russ. J. Bioorganic Chem.* **2007**, *33*, 24–37.

(18) Pereira, C. S.; Kony, D.; Baron, R.; Müller, M.; van Gunsteren, W. F.; Hünenberger, P. H. Conformational and Dynamical Properties of Disaccharides in Water: A Molecular Dynamics Study. *Biophys J* **2006**, *90*, 4337–4344.

(19) Damager, I.; Engelsen, S. B.; Blennow, A.; Møller, B. L.; Motawia, M. S. First Principles Insight into the Alpha-Glucan Structures of Starch: Their Synthesis, Conformation, and Hydration. *Chem Rev* **2010**, *110*, 2049–2080.

(20) Patel, D. S.; Pendrill, R.; Mallajosyula, S. S.; Widmalm, G.; MacKerell, A. D., Jr. Conformational Properties of α- or β-(1→6)-Linked Oligosaccharides: Hamiltonian Replica Exchange MD Simulations and NMR Experiments. *J. Phys. Chem. B* **2014**, *118*, 2851–2871.

(21) Deshmukh, S. A.; Sankaranarayanan, S. K. R. S.; Mancini, D. C. Atomic Scale

Characterization of the Conformational Dynamics of a Thermo-Sensitive and a Non-Thermo-Sensitive Oligomer Using Vibrational Spectra Obtained from Molecular Dynamics. *Polymer* **2012**, *53*, 1306–1320.

(22) Deshmukh, S. A.; Sankaranarayanan, S. K. R. S.; Mancini, D. C. Vibrational Spectra of Proximal Water in a Thermo-Sensitive Polymer Undergoing Conformational Transition across the Lower Critical Solution Temperature. *J. Phys. Chem. B* **2012**, *116*, 5501–5515.

(23) Ali, S. A.; Hassan, M. I.; Islam, A.; Ahmad, F. A Review of Methods Available to Estimate Solvent-Accessible Surface Areas of Soluble Proteins in the Folded and Unfolded States. *Curr. Protein Pept. Sci.* **2014**, *15*, 456–476.

(24) Ortiz de Solorzano, I.; Bejagam, K. K.; An, Y.; Singh, S. K.; Deshmukh, S. A. Solvation Dynamics of N-Substituted Acrylamide Polymers and the Importance for Phase Transition Behavior. *Soft Matter* **2020**, *16*, 1582–1593.

(25) Joshi, S. Y.; Singh, S.; Deshmukh, S. A. Coarse-Grained Molecular Dynamics Integrated with Convolutional Neural Network for Comparing Shapes of Temperature Sensitive Bottlebrushes. *npj Computational Materials* **2022**, *8*, 1–12.

(26) Eisenhaber, F.; Lijnzaad, P.; Argos, P.; Sander, C.; Scharf, M. The Double Cubic Lattice Method: Efficient Approaches to Numerical Integration of Surface Area and Volume and to Dot Surface Contouring of Molecular Assemblies. *J. Comput. Chem.* **1995**, *16*, 273–284.

(27) Wohlert, M.; Benselfelt, T.; Wågberg, L.; Furó, I.; Berglund, L. A.; Wohlert, J. Cellulose and the Role of Hydrogen Bonds: Not in Charge of Everything. *Cellulose* **2022**, *29*, 1–23.

(28) Chen, P.; Nishiyama, Y.; Putaux, J.-L.; Mazeau, K. Diversity of Potential Hydrogen Bonds in Cellulose I Revealed by Molecular Dynamics Simulation. *Cellulose* **2014**, *21*, 897–908.

(29) Abraham, M. J.; Murtola, T.; Schulz, R.; Páll, S.; Smith, J. C.; Hess, B.; Lindahl, E. GROMACS: High Performance Molecular Simulations through Multi-Level Parallelism from Laptops to Supercomputers. *SoftwareX* **2015**, *1-2*, 19–25.

(30) Chandra, A. Effects of Ion Atmosphere on Hydrogen-Bond Dynamics in Aqueous Electrolyte Solutions. *Phys. Rev. Lett.* **2000**, *85*, 768–771.

(31) Róg, T.; Murzyn, K.; Milhaud, J.; Karttunen, M.; Pasenkiewicz-Gierula, M. Water Isotope Effect on the Phosphatidylcholine Bilayer Properties: A Molecular Dynamics Simulation Study. *J. Phys. Chem. B* **2009**, *113*, 2378–2387.

(32) Rukmani, S. J.; Kupgan, G.; Anstine, D. M.; Colina, C. M. A Molecular Dynamics Study of Water-Soluble Polymers: Analysis of Force Fields from Atomistic Simulations. Mol. Simul. 2019, 45, 310–321.
